# Development of the squamate naso-palatal complex: detailed 3D analysis of the vomeronasal organ and nasal cavity in the brown anole *Anolis sagrei* (Squamata: Iguania)

**DOI:** 10.1186/s12983-020-00369-7

**Published:** 2020-09-22

**Authors:** Paweł Kaczmarek, Katarzyna Janiszewska, Brian Metscher, Weronika Rupik

**Affiliations:** 1 Institute of Biology, Biotechnology and Environmental Protection, Faculty of Natural Sciences, University of Silesia in Katowice, Bankowa 9, 40-007 Katowice, Poland; 2 Institute of Paleobiology, Polish Academy of Sciences, Twarda 51/55, 00-818 Warsaw, Poland; 3 Department of Evolutionary Biology, University of Vienna, Althanstrasse 14, 1090 Vienna, Austria

**Keywords:** Nasal cavity, VNO, Jacobson’s organ, Palate, Facial prominences, Lizard embryos

## Abstract

**Background:**

Despite the diverse morphology of the adult squamate naso-palatal complex – consisting of the nasal cavity, vomeronasal organ (VNO), choanal groove, lacrimal duct and superficial palate – little is known about the embryology of these structures. Moreover, there are no comprehensive studies concerning development of the nasal cavity and VNO in relation to the superficial palate. In this investigation, we used X-ray microtomography and histological sections to describe embryonic development of the naso-palatal complex of iguanian lizard, the brown anole (*Anolis sagrei*). The purpose of the study was to describe the mechanism of formation of adult morphology in this species, which combines the peculiar anole features with typical iguanian conditions. Considering the uncertain phylogenetic position of the Iguania within Squamata, embryological data and future comparative studies may shed new light on the evolution of this large squamate clade.

**Results:**

Development of the naso-palatal complex was divided into three phases: early, middle and late. In the early developmental phase, the vomeronasal pit originates from medial outpocketing of the nasal pit, when the facial prominences are weakly developed. In the middle developmental phase, the following events can be noted: the formation of the frontonasal mass, separation of the vestibulum, appearance of the lacrimal duct, and formation of the choanal groove, which leads to separation of the VNO from the nasal cavity. In late development, the nasal cavity and the VNO attain their adult morphology. The lacrimal duct establishes an extensive connection with the choanal groove, which eventually becomes largely separated from the oral cavity.

**Conclusions:**

Unlike in other tetrapods, the primordium of the lacrimal duct in the brown anole develops largely beyond the nasolacrimal groove. In contrast to previous studies on squamates, the maxillary prominence is found to participate in the initial fusion with the frontonasal mass. Moreover, formation of the choanal groove occurs due to the fusion of the vomerine cushion to the subconchal fold, rather than to the choanal fold. The loss or significant reduction of the lateral nasal concha is secondary. Some features of anole adult morphology, such as the closure of the choanal groove, may constitute adaptations to vomeronasal chemoreception.

## Background

**Fig. 1:**
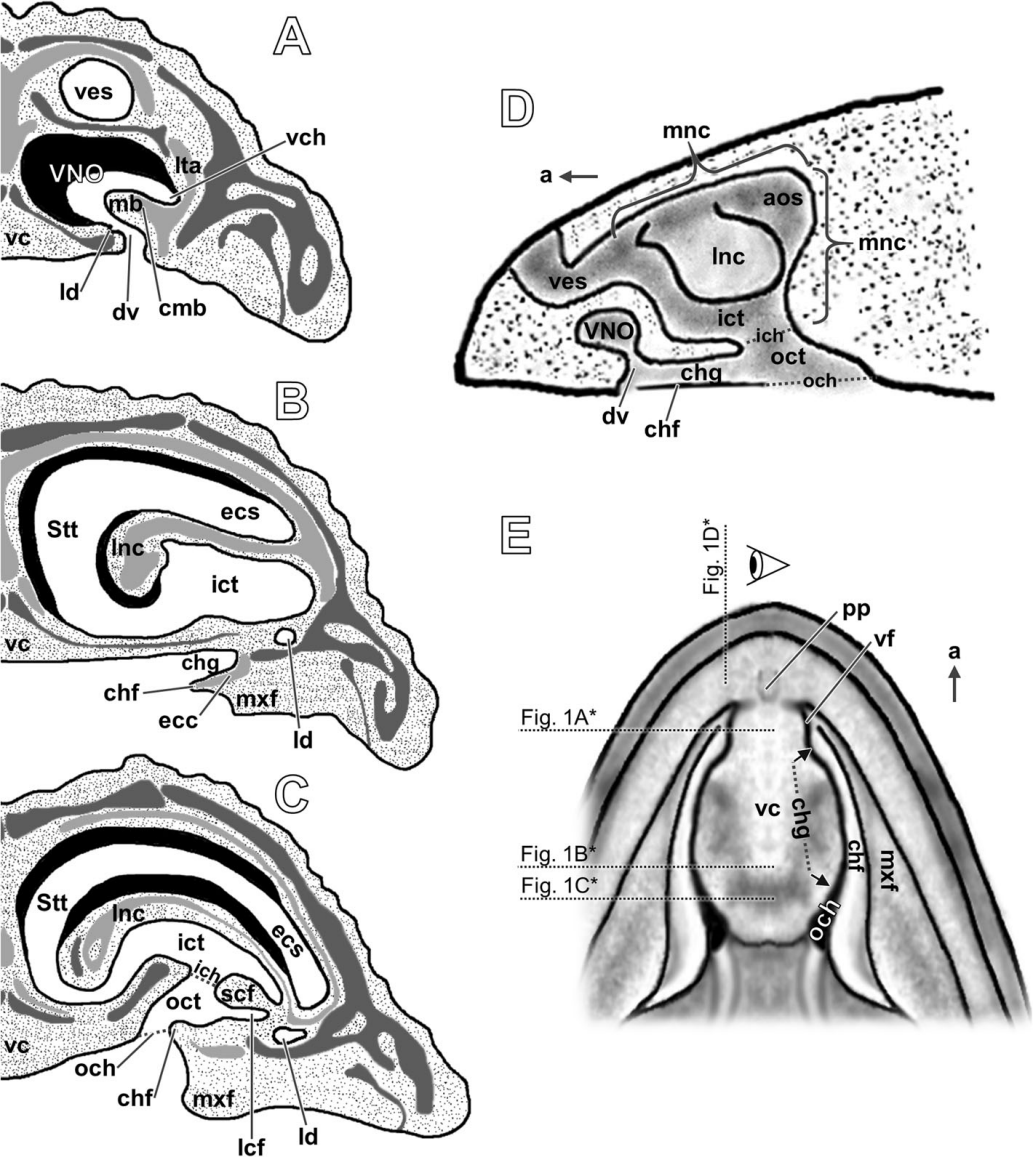
Diagrammatic illustration of the general morphology of the squamate naso-palatal complex. Transverse sections through the snout at the level of the VNO (**a**), the choanal groove (**b**) and the outer choana (**c**). Figs. **a** and **b**: *Phyllodactylus*, modified from [5]; **c**: *Hemidactylus*, modified from [13]. **d** Longitudinal cutaway through the snout of *Lacerta* at the level of the right choanal groove showing the nasal cavity from the medial side. **e** Ventral view of the palate of *Gekko gecko*. Figs. **d** and **e**: modified from [5]. * Note that drawings show different species. The levels of the sections (for **a**–**c**) and cutaway (for **d**) shown in **e** facilitate understanding a general squamate morphology, but may be inaccurate when considering the detailed anatomy of particular taxa. Abbreviations: *a* anterior, *aos* antorbital space, *chf* choanal fold, *chg* choanal groove, *cmb* cartilage of the mushroom body, *dv* duct of the VNO, *ecc* ectochoanal cartilage, *ecs* extraconchal space, *ich* inner choana, *ict* inner choanal tube, *lcf* lateral choanal fissure, *ld* lacrimal duct, *lnc* lateral nasal concha, *lta* lamina transversalis anterior, *mb* mushroom body, *mnc* main nasal cavity, *mxf* maxillary fold, *och* outer choana, *oct* outer choanal tube, *pp* premaxillary papilla, *scf* subconchal fold, *Stt* Stammteil, *vc* vomerine cushion, *vch* ventral channel, *ves* vestibulum, *vf* vomeronasal fenestra

Two anatomically separate olfactory systems operate in squamate reptiles. The main olfactory system is responsible for detection of airborne volatiles, while the accessory olfactory or vomeronasal system is also sensitive to high molecular-weight molecules [1]. The sensory olfactory epithelium of the nasal cavity and the vomeronasal sensory epithelium of the Jacobson’s or vomeronasal organ (VNO) constitute the peripheral parts of the main and accessory olfactory systems respectively [2, 3]. The VNO originates from ventromedial outpocketing of the nasal pit. Unlike in other tetrapods, in squamate embryos it loses its direct connection with the nasal cavity, and in adult forms paired VNOs enter the oral cavity exclusively on the anterior part of the palate [4]. However, in some lizards the duct of the VNO is also associated with the choanal groove, which in turn is confluent with the internal naris [5, 6]. Due to the connection to the mouth, the organ’s sensory function depends on chemicals collected by the tongue [7–10]. It has been suggested that volatile molecules detected by the main olfactory system may provoke tongue flicking behavior to stimulate the vomeronasal sensory system. The latter seems to provide more qualitative discrimination and allow for prey or mate trailing [8, 11, 12]. The main mass of the squamate VNO (Fig. 1a) constitutes the dorsal dome formed by the sensory epithelium [14–16]. The ventral concha, called the mushroom body, reduces the lumen of the VNO [15, 17, 18]. It is supported by the cartilage, which protrudes from the lamina transversalis anterior [17, 19]. The VNO lumen extends below the sensory epithelium to form the ventral (or spiral) channel, which is confluent with the duct of the organ [17, 20]. In adult squamates the nasal cavity consists of three major parts: the vestibulum, main nasal cavity and nasopharyngeal duct or part of the choanal tube homologous to it [6, 18], called here the outer choanal tube (Fig. 1a–d). The vestibulum (= anterior chamber, atrium) leads to the external naris anteriorly and to the main nasal cavity (= *cavum nasi proprium*, olfactory chamber, true nasal cavity) posteriorly.

In most squamates the largest part of the main nasal cavity is characterized by the presence of the single concha projecting into it from the lateral side [17, 21, 22]. Traditionally, this part of the main nasal cavity, called the conchal zone, may be subdivided into 3 zones: the extraconchal space (= *Sakter*), the Stammteil, and the choanal tube (= *Choanengang*) [5, 18, 22], called here the inner choanal tube (Figs. 1b, c). There are no clear anatomical borders between each mentioned part, and all of them may be distinguished mostly on the basis of their position relative to the lateral nasal concha. The antorbital space (= *Antorbitalraum*) is a part of the main nasal cavity located posterior to the conchal zone [5, 21] (Fig. 1d). In general, the olfactory sensory epithelium lines the dorsal and medial walls of the main nasal cavity and dorsal surface of the lateral nasal concha [15, 17, 21, 23].

The main nasal cavity passes through the inner choana to the outer choanal tube leading to the oral cavity through the outer choana (Figs. 1c, d). In the outer choanal tube the lateral or dorsolateral diverticulum may be distinguished: it is the lateral choanal fissure. Anteriorly, the lateral choanal fissure is confluent with the choanal groove (= *Choanenrinne*), which extends beyond the outer choanal tube and may be confluent, in some forms, with the duct of the VNO [22] (Fig. 1). In general, the choanal groove establishes more or less extensive connection with the anterior part of the lacrimal duct (= nasolacrimal duct, lachrymal duct), which in most non-ophidian squamates emerges from the conjunctival or sub-brillar space as two canaliculi. The anteriormost tip of the lacrimal duct is usually associated with the VNO duct [6, 24–26]. The medial border of the outer choana, as well the choanal groove, is formed by the lateral edge of the raised median palatal plate called the vomerine cushion (= vomerine pad, *Vomerpolster*) [5, 22, 27]. The choanal fold (= ectochoanal fold, *Choanenfalte*) forms the lateral border of the outer choana and the floor of the choanal groove. Fuchs [5] also recognized “*mediale Seitenfalten*” (called here the maxillary fold) located more ventrally to the choanal fold (Fig. 1b, c and e).

In this study, embryonic development of the VNO and associated structures in the brown anole (*Anolis sagrei*) was investigated. It is a representative of the family Dactyloidae (anoles) and the large squamate clade Iguania [28, 29]. Anoles may be assigned to various ecomorphotypes (or ecomodes) which reflect the lizards’ habitat preferences and which, in most cases, are associated with an arboreal lifestyle [30–33]. It has been suggested that the arboreal lifestyle is related to the loss of chemosensory function of VNO in anoles and the other arboreal forms [17]. The characteristic features of olfactory organs and associated structures in anoles are as follows: small VNO and limited area of sensory olfactory epithelium (which corresponds with small sizes of the main and accessory olfactory bulbs) [2], lack of the nasal concha [2, 17, 34], reduction [6] or lack of the mushroom body [17], closure of the choanal groove [6, 35, 36], short and well posteriorly located choana [22], and reduced size of the lateral nasal gland (external nasal gland) [17, 22, 34].

At least some of these morphological features may confirm the notion about weak chemosensory abilities in anoles. In fact, like the other members of the large clade Iguania, the brown anole is traditionally considered to be rather “visually oriented,” or at least not very specialized for olfactory/vomeronasal chemoreception, using a sit-and-wait strategy and tongue prehension to catch their prey [37, 38]. Like the other representatives of Iguania, the olfactory organs of the anole are characterized by the following features: connection of the choanal groove with the VNO duct, close association of the lacrimal duct and the choanal groove, connection of the lacrimal duct with the medial wall of the VNO duct [6] and lack of the well-developed bony enclosure of the VNO called the cupola Jacobsoni [39].

The review of the literature concerning the adult morphology of the olfactory organs and associated structures in squamates [6, 15–17, 24, 26, 34, 37, 40–43] reveals huge variation in the naso-palatal complex. Some studies indicate a great diversity within one superfamily, e.g. the morphology of the nasal cavity in Pleurodonta [34]. However, others suggest high conservatism at the family or larger clade level, e.g. concerning the relationships between the VNO duct, choanal groove, and lacrimal duct [6]. There is also some evidence for convergent evolution, e.g. reduction of the choanal groove in snakes and monitor lizards [37]. In contrast to adult morphology, the embryonic development of the squamate VNO or nasal cavity received only limited investigation. Still little is known about the mechanisms leading to the formation of the various adult conditions. Most of the embryological studies were performed on “morphological” scleroglossans (e.g. [5, 6, 20, 44, 45]). As far as we know, the embryonic development of such structures has never been studied in Dactyloidae. Only a few analyses have concerned the embryology of the other members of Iguania, providing general descriptions of developing olfactory organs in agamid lizards [46, 47] and the green iguana [48] or analyzing differentiation of the olfactory and associated epithelia in the green iguana [49].

The discordance between the morphological and molecular phylogeny of squamate reptiles and still unresolved phylogenetic position of Iguania [50] make the study of embryonic development even more important, and potentially useful for phylogenetic inference [51]. Moreover, there is a lack of substantial studies that consider development of the nasal cavity and VNO in relation to the superficial (soft-tissue) palate. The purpose of this study was to investigate the embryonic development of the naso-palatal complex (including the VNO, nasal cavity, choanal groove, lacrimal duct, and superficial palate) in the brown anole and to use these data to obtain a better description of general processes in squamate embryology and better understanding of the mechanism of formation of specific adult conditions of anoles and other Iguania. In the present investigation, both light microscopy and X-ray microtomography (XRM) were used. Histological serial sections were used to facilitate interpretation of tomographic data and to perform general histological description, and tomographic reconstructions were used to perform 3D visualizations of analyzed structures.

## Methods

### Animal care and embryo preparation

The eggs of the brown anole were obtained from animals kept in the breeding room of the Faculty of Natural Sciences of the University of Silesia in Katowice. One male and two females were housed in the terrarium of dimensions L 35 cm x W 35 cm x H 60 cm. The terrarium contained cork tubes, a structural background and live plants to allow climbing and provide hiding places. Moist coconut fiber substrate was used in the terrarium, which at the same time constituted the substrate for egg incubation. A 35 W spot bulb and a compact fluorescent UVB bulb (Exo Terra, REPTILE UVB100) were provided. The temperature in the basking place was approximately 31**–**33 °C, while in the cool area it was approximately 23–25 °C. During the night, the temperature in the entire terrarium was about 23–25 °C. Spraying the terrarium with water was performed usually twice a day to maintain proper humidity. Additionally, two water dishes were kept inside the terrarium. Anoles were fed with crickets two or three times a week. Each time insects were dusted with powder composed of Sera Reptimineral C vitamins and sepia calcium powder in a 1:3 volume ratio.

The eggs were collected 4 times a year from the terrarium substrate. Embryos for the present study were collected during a period of 3 years. During this time 43 embryos were sacrificed and used for the different research methods (Table S1). Prior to isolation the embryos inside the eggs were sedated by cooling in a refrigerator for about 20 min and then placed on ice (still in the refrigerator) for about 15**–**20 min (see: [52, 53]). Then, the embryos were isolated, decapitated and immediately transferred to the fixative (see below). The age of the embryos was calculated according to the developmental table for *Anolis*, based on external morphological characteristic [54].

### Light microscopy

For histological study, the embryos (31 specimens in total) were fixed in Bouin’s solution for 48 h. Embryos older than stage 10 were decalcified in 10% formic acid for 2 weeks, then rinsed in distilled water and, like younger embryos, dehydrated and embedded in paraffin. The heads were serially cut at 7–10 μm using a Leica rotary microtome (Leica RM2125RT). Paraffin sections were stained with H&E [55, 56]. The histological sections were sequentially photographed, using an OLYMPUS BX60 light microscope with an OLYMPUS DP12 digital camera. The images were saved as TIFF files using the Olympus cellSens Standard software. The images in the stack were aligned using TrakEM2 plug-in in ImageJ software [57]. The aligned stack slices were used for comparisons to the XRM data.

### X-ray microtomography and 3D reconstructions

Whole-sample 3D images were made using non-destructive contrast-enhanced XRM (microCT) [58–61]. The embryos (12 specimens in total) were fixed in a 1:1 mixture of 2.5% glutaraldehyde and 2% paraformaldehyde in 0.1 M phosphate buffer (pH 7.4) at 4 °C [62, 63]. After rinsing in phosphate buffer the samples were rinsed in distilled water and then stained in 3.75% w/v in Lugol solution for 48 h to enhance the X-ray contrast of soft tissues [58, 59]. After staining, the samples were rinsed in water and dehydrated to 70% ethanol for storage. To fill the gap in the developmental sequence, one sample (an embryo at stage 8) was obtained from material designated for light microscopy. After fixation in Bouin’s solution and dehydration to 80% ethanol, it was stained in 0.3% PTA in 70% ethanol for 72 h for contrast enhancement [59, 61]. Samples were scanned in 70% ethanol or in 0.5% w/v agarose LMT [60]. The scan parameters and XRM imaging systems for each sample are shown in Table S2.

Tomographic reconstructions were made with XMReconstructor (Zeiss-Xradia) and results were exported as TIFF images. Segmentations and visualizations of the analyzed structures were made in Drishti v.2.6.3 and v.2.6.4 [64].

### Structure interpretation and anatomical axes

The nasal cavity was defined in similar way as VNO and other structures: the epithelium + lumen, rather than lumen only (Fig. S1A). Segmentations included epithelium of analyzed structures and cellular plugs. The segmentations did not include lumens (the endocasts of the lumens are not displayed as colored spaces in 3D images) (Fig. S1A). The nasal capsule or bones were not considered to be an integral part of the nasal cavity. The antero-posterior axis of the embryonic head, at different developmental stages, was aligned according to the long axis of the maxillary prominences or maxilla (upper lip) (Fig. S1B). The segmented structures were rendered as volumes. Individual colormaps were used for each of analyzed structures. In some cases the rendering and transparency of the nasal cavities were adjusted to show the sensory olfactory epithelium (non-transparent green) and non-sensory (respiratory) epithelium (semi-transparent green) (see [59, 61]). Such adjusted rendering and transparency revealed also the nasal plug, which exhibited relatively high X-ray density after contrast staining (in stages 14 and 17 labelled as yellow green).

## Results

Development of the naso-palatal complex including the nasal cavity and VNO involves many different structures of the anterior part of the embryonic head. Therefore, for a better description of analyzed processes the results were divided into 3 developmental phases: early, middle, and late. The results of this study are summarized in Table 1.

**Table 1:** Main developmental events during embryonic development of the naso-palatal complex in the brown anole

Phase	Stage^a^	Facial prominences, superficial palate and choanal groove	Nasal cavity	VNO	Lacrimal duct
**Early development**	**2–3**	**facial prominences** weakly developed	**nasal pit** present; round **primitive naris** open on the ventrolateral surface of the forming snout	**vomeronasal pit** may appear in some specimen at stage 3 on medial wall of the **nasal pit**	
	**5–5/6**	paired primordium of the **vomerine cushion** present; **nasolacrimal groove**, between the lateral nasal prominence and the maxillary prominence, well visible	clearly elongated **primitive naris** open ventrally; **primitive choanal tube** and primordia of the: **Stammteil**, **extraconchal space** (stage 5/6) and **lateral nasal concha** (stage 5/6) distinguishable	**vomeronasal pit** well visible on medial wall of the **primitive choanal tube**	
**Middle development**	**6**	**facial prominences** well visible; paired primordia of the **vomerine cushion** and **anterior segment of the palate** present	anteriormost part of the **primitive choanal tube** filled with the **nasal plug**; posterior extension of the nasal pit (part of the primitive choanal tube) well visible	vomeronasal pit spherical in shape (**early VNO**)	
	**7**	**frontonasal mass** formed; **lateral nasal prominence** and the **frontonasal mass** in close apposition through their entire lengths *(a)*; **maxillary prominence** approaches to the **frontonasal mass**; **maxillary fold** present; paired primordium of the **anterior segment of the palate** almost obliterated	**early nasal cavity**: primordium of vestibulum (still part of the primitive choanal tube) relatively well distinguishable *(a)*; **presumptive external naris** narrowed *(a)*; the **extraconchal space** well visible	distance between the **early VNO** and the **primitive naris** increased	
	**8**	**lateral nasal prominence** and the **maxillary prominence** fused with the **frontonasal mass** *(b)*; paired primordium of the **anterior segment of the palate** well visible again	division of the **primitive naris** into the **external naris** and **primitive outer choana** *(b)*; **vestibulum** well defined *(b)*; small **antorbital space** present; **extraconchal space** well developed, but restricted approximately to the posterior half of the primordial main nasal cavity; **choanal diverticulum** formed	ellipsoidal shape of the **early VNO**; narrowed connection of the **early VNO** and the **primitive choanal tube**	primordium of the **lacrimal duct** present in deep part of the groove between the **lower eyelid** primordium and the **maxillary prominence**
	**9**	**upper lip** and the **anterior segment of the palate** well formed; **vomerine cushion** visible as a single structure; **choanal fold** visible	**vestibulum** of tubular form; vertical orientation of the **extraconchal space** and its restriction to the posterior third of the main nasal cavity primordium; **primitive inner choana**, **primitive inner choanal tube** and the **primitive outer choanal tube** distinguishable; **subconchal fold** visible	primordium of the **VNO duct** distinguishable from the anterior most part of the **primitive outer choanal tube** (early VNO can be considered as the VNO); small primordial **mushroom body** visible	both **lacrimal canaliculi** visible; anterior part of the lacrimal duct (anterior to the anterior lacrimal canaliculi) independent from the groove between the **lower eyelid** primordium and the **maxillary prominence**
	**9/10–10**	anterior part of the **primitive outer choana** transformed into opening of the **choanal groove** *(c)*	anterior part of the **primitive inner choana** closed by fusion of the **subconchal fold** and the **vomerine cushion** (formation of the **choanal groove**) *(c)*; initial bud of the **lateral nasal gland** well distinguishable	**VNO** separated from the nasal cavity *(c)*	**lacrimal duct** entirely detached from the groove between the **lower eyelid** and the **maxillary prominence**; **posterior** and **anterior lacrimal canaliculi** clearly in contact to the inner surface of the lower eyelid
**Late development**	**11–12**	anterior third of the **choanal groove** more widely open to the oral cavity in contrast to the rest part of this structure restricted by the **choanal fold** and the convex **vomerine cushion**	**lateral nasal gland** characterized by the main duct primordium and the spherical bud with few clefts; **extraconchal space** restricted to the posterior end of the main nasal cavity; **antorbital space** visible as a small vertical diverticulum	tubular-like **VNO duct**; pigment granules present in the central layer of the **vomeronasal sensory epithelium** (stage 12)	**rostral plate of the lacrimal duct** connected to the anterior half of the **choanal groove**
	**14**	posterior part of the **choanal groove** widely open to the oral cavity; opening of the anterior third of the **choanal groove** narrowed slightly by the vomerine cushion; borders of the **anterior segment of the palate** obliterated	**lateral nasal gland** composed of many tubulo-acinar units; dorsal part of the **extraconchal space** drawn out beyond the level of the **antorbital space**		triangular shape of the **lacrimal duct rostral plate** (its posterior expansion distinguishable); anteriormost part of the **lacrimal duct** at the border between the **VNO duct** and the **choanal groove**
	**17**	**choanal fold** well developed; **maxillary fold** less distinct	**vestibulum** almost horizontal; compound tubuloacinar **lateral nasal gland**; **extraconchal space** drawn out posteromedially and dilated (lack the **lateral nasal concha**, except its anterior extension between the Stammteil and inner choanal tube); **sensory olfactory epithelium** restricted to the anterodorsal part of the main nasal cavity; entire **outer choanal tube** dilated	**cartilage of the mushroom body** well distinguishable; two parts of the **VNO duct** well visible: just ventral to the VNO and anterolateral extension	anteriormost part of the **lacrimal duct** expanded more anteriorly along the medial wall of the **VNO duct**
	**18**	**choanal fold** apposed to lateral ridge of the **vomerine cushion** (opening of the **choanal groove** to the oral cavity reduced)	**nasal plug** usually reduced or absent; compound tubular **lateral nasal gland**; lumen of the main nasal cavity increased (dilatated): lateral edge of the **extraconchal space** almost completely obliterated	**ventral channel** more distinct; rhombus-like shape of the **VNO** in transverse section; **VNO lumen** large; **vomeronasal sensory epithelium**: numerous pigment granules, hyperchromatic nuclei of the cells of basal and central layers	posterior expansion of the **lacrimal duct rostral plate** almost reaching the level of the **outer choana**; **lacrimal duct**, including **lacrimal canaliculi**, usually patent
	**19**	choanal fold fused with vomerine cushion (closed **choanal groove** formed)	**nasal plug** absent		**lacrimal duct**, including **lacrimal canaliculi**, patent

*(a*, *b, c)* closely associated developmental events

^a^according to Sanger et al. [54]

### Early developmental phase (stages 2–5/6)

In this developmental phase the main events involve differentiation of the nasal and vomeronasal pits. The facial prominences (lateral nasal, medial nasal, and maxillary) are relatively weakly developed. The stage in which the nasal placode is present was not found in this study.

**Fig. 2:**
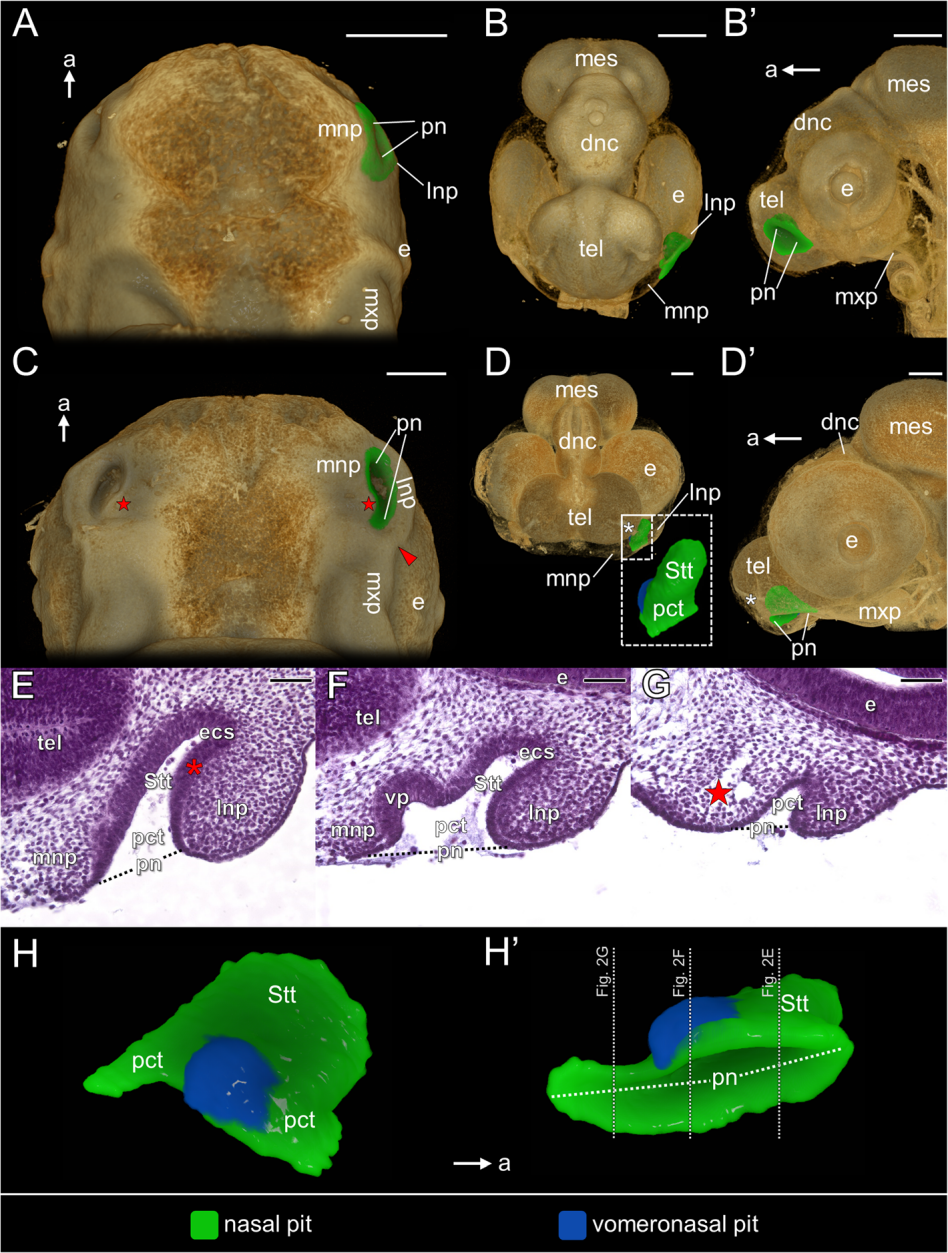
Early developmental phase of the naso-palatal complex of the brown anole based on 3D reconstructions (**A**–**D’** and **H**–**H’**) and transverse histological sections at levels shown in slightly younger embryo in Fig. H’ (**E**–**G**): ventral (**A**), anterior (semi-transparent) (**B**) and lateral (semi-transparent) view (**B’**) of developing head at stage 3; ventral (**C**), anterior (semi-transparent) (**D**) and lateral (semi-transparent) view (**D’**) of developing head at stage 5; sections through the nasal pit showing anterior (**E**) middle (**F**) and posterior part (**G**) of this structure at stage 5/6; medial (**H**) and ventromedial view (**H’**) of the nasal pit at stage 5. Abbreviations: *a* anterior, *dnc* diencephalon, *e* eye, *ecs* primordial extraconchal space, *lnp* lateral nasal prominence, *mes* mesencephalon, *mnp* medial nasal prominence, *mxp* maxillary prominence, *pct* primitive choanal tube, *pn* primitive naris, *Stt* primordial Stammteil¸ *tel* telencephalon, *vp* vomeronasal pit. *Red star* primordium of the vomerine cushion, *red asterisk* primordial lateral nasal concha; *white asterisk* olfactory nerve, *red arrowhead* nasolacrimal groove. Scale bars 200 μm (3D) and 50 μm (histological sections)

At the beginning of this phase (stages 2–3), shallow nasal pits are located lateral to the developing telencephalon and anteroventral to the primordia of the eyes (Figs. 2A–B’). Each of them is bounded medially by the medial nasal prominence and laterally by the lateral nasal prominence (Figs. 2A, B). Round primitive nares open ventrolaterally and each of them is surrounded by an epithelial band of the nasal pit (Figs. 2A, B’). The medial wall of the nasal pit closely approaches the developing telencephalon (Fig. 2B).

At the end of this developmental phase (stages 5–5/6) the primitive naris is clearly elongated and, due to the ventral growth of the nasal prominences (especially the lateral one), it opens on the ventral surface of the forming snout (Figs. 2C–G). The lateral border of the nasal pit is still formed by the lateral nasal prominence exclusively. The medial border of the nasal pit is formed by the medial nasal prominence and the primordium of the vomerine cushion (red stars in Fig. 2C). Both primordia of the vomerine cushion are separated from each other by a significant distance. The lateral nasal prominence and the anterior part of the maxillary prominence are separated by the nasolacrimal groove (red arrowhead in Fig. 2C). The maxillary prominences are relatively better developed at this time (Fig. 2C, D’). At these stages, the main mass of the nasal pit constitutes its anterior part, probably due to the pressure exerted by the developing eye (Fig. 2D’). This part of the nasal pit is mostly composed of thick epithelium (Fig. 2E, F), which, at least in the larger part, constitutes the differentiating sensory olfactory epithelium, since it is associated with the olfactory nerve (white asterisk in Fig. 2D and D’). The occurrence of this epithelium is restricted to the part of the nasal pit, which is now the primordium of the Stammteil (Fig. 2D–H’). The differentiating Stammteil passes into the extraconchal space primordium, which becomes visible at about stage 5/6, when it is slightly developed in the dorsolateral part of the nasal pit (Fig. 2E, F). The medial wall of the lateral nasal prominence forms the primordium of the lateral nasal concha (red asterisk in Fig. 2E). At developmental stages 5–5/6, the part of the nasal pit connecting the primordial Stammteil with the primitive naris forms the primitive choanal tube (Fig. 2E–H’). The differentiating sensory olfactory epithelium of the Stammteil decreases in thickness ventrally and posteriorly passes into the non-sensory epithelium of the primitive choanal tube. In the anterior-posterior axis the changes in the thickness of the nasal pit epithelium occurs more abruptly on the surface formed by the medial wall of the lateral nasal prominence (Fig. 2E–G). The primitive choanal tube runs posteriorly beyond the level of the primordial Stammteil to form the posterior extension of the nasal pit (Fig. 2C and G–H’). In this part of the nasal pit, the thickness of epithelium forming it is reduced in some parts to a single layer of cuboidal or cylindrical cells (Fig. 2G). The vomeronasal pit may appear at stage 3 on the medial wall of the nasal pit, but in two of three studied specimens at stage 4 this structure was absent. At stages 5–5/6, the vomeronasal pit is well distinguishable and it enters the primitive choanal tube (Figs. 2F, H and H’). The thickness of the vomeronasal sensory epithelium is equal to the thickness of the thickest part of the differentiating sensory olfactory epithelium (Fig. 2E, F).

### Middle development (developmental stages 6–10)

The differentiation of the nasal cavities and VNOs are strongly related to the growth and fusions of the facial prominences forming the entire snout and the superficial palate. Alongside that, the lacrimal duct develops, but at the end of this phase it constitutes a separate structure. Thus it was described separately.

#### Development of the superficial palate, nasal cavity and VNO

**Fig. 3:**
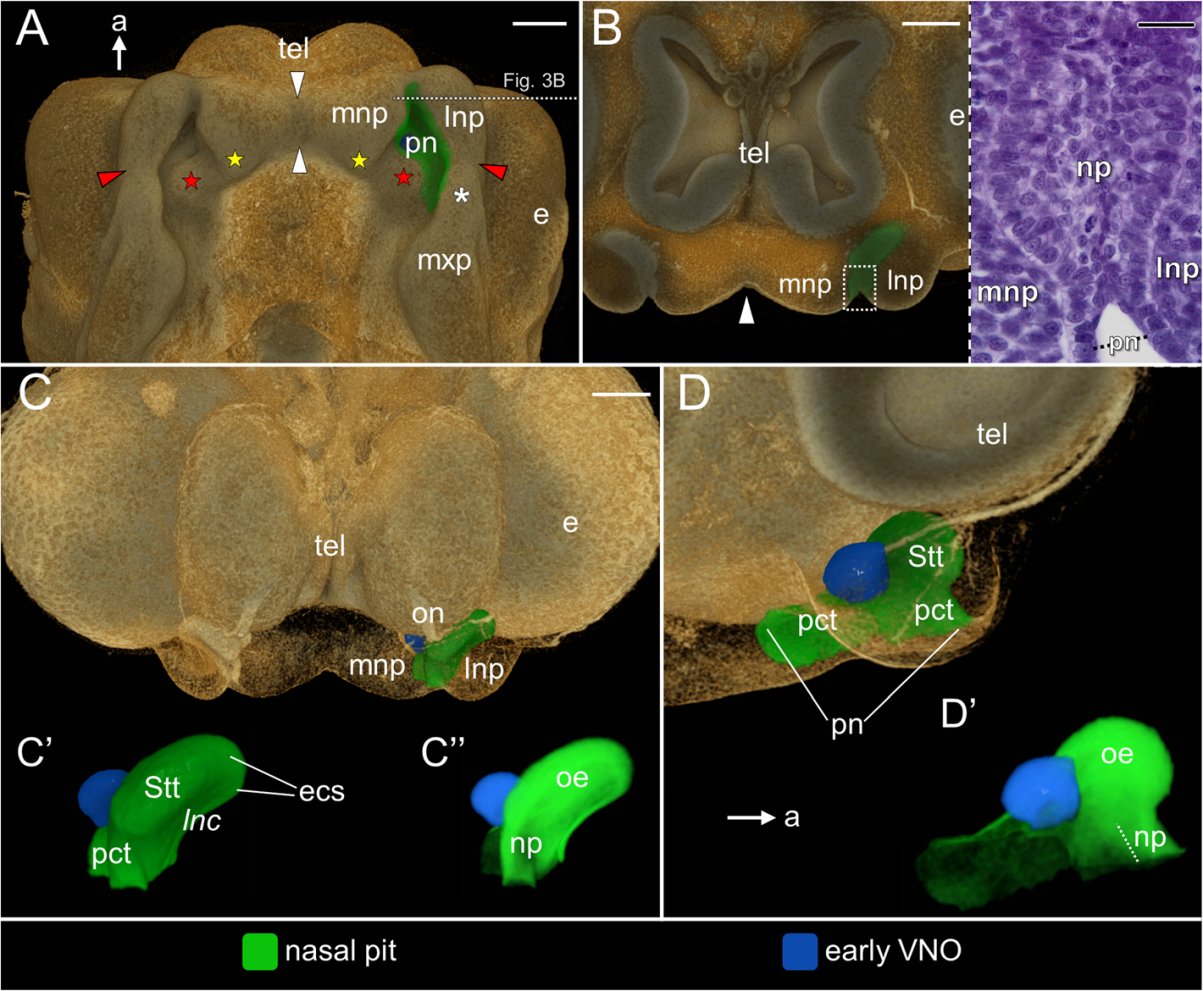
The naso-palatal complex in the brown anole at stage 6. **A** Ventral view of the palate. **B** Transverse cutaway through the snout at the level of the nasal plug (left) and transverse histological section through the nasal plug (right); the level of the section (cutaway) shown in Fig. **A**. **C** Anterior view of the semi-transparent snout. **C′** Zoomed anterior view of the left nasal pit. **C″** Zoomed anterior view of the left nasal pit rendered with partial opacity. **D** Sagittal cutaway of the semi-transparent snout showing the medial view of the left nasal pit. **D’** Zoomed medial view of the left nasal pit rendered with partial opacity. Abbreviations: *a* anterior, *e* eye, *ecs* primordial extraconchal space, *lnc* primordial lateral nasal concha, *lnp* lateral nasal prominence, *mnp* medial nasal prominence, *mxp* maxillary prominence, *np* nasal plug, *oe* sensory olfactory epithelium, *on* olfactory nerve, *pct* primitive choanal tube, *pn* primitive naris, *Stt* primordial Stammteil, *tel* telencephalon*. Red star* primordium of the vomerine cushion, *yellow star* primordium of the anterior segment of the palate, *white asterisk* anterior part of the maxillary prominence, *red arrowhead* nasolacrimal groove, *white arrowhead* furrow between the medial nasal prominences. Note: italicized label on 3D image (*lnc*) indicates the concavity in the nasal pit. Scale bars 200 μm (3D) and 20 μm (histological section)

At **stage 6** the facial prominences are easily visible (Fig. 3A). The medial nasal prominences are confluent dorsally, but ventrally there is a relatively deep median furrow separating them (white arrowheads in Figs. 3A and B). The posterior extensions of the medial nasal prominences, forming two primordia of the anterior segment of the palate, become visible (yellow stars in Fig. 3A). They are located anteromedial to the ipsilateral primordia of the vomerine cushion (red stars in Fig. 3A). The lateral nasal prominence is still separated from the maxillary prominence by the nasolacrimal groove (red arrowhead in Fig. 3A). The maxillary prominence is composed of a larger posterior part, swelled medially, and smaller anterior portion (white asterisk in Fig. 3A). Anterior parts of the medial and lateral nasal prominences approach each other, narrowing the anteriormost part of the primitive naris (Fig. 3A, B). Posterior to this region, the nasal prominences, medial and lateral, are still at a significant distance to each other, leaving the primitive naris widely open (Fig. 3A). The posteriormost part of the nasal pit, formed by the primitive choanal tube, extends beyond the level of the nasolacrimal groove (red arrowhead) and the primordium of the vomerine cushion (red star), achieving almost the level of the border between the anterior and posterior portion of the maxillary prominence (Fig. 3A). For this reason, the posterior part of the nasal pit is bounded laterally by the anterior portion of the maxillary prominence.

The vomeronasal pit may be called the early VNO since it takes a spherical shape (Fig. 3C–D’). It is still connected with the nasal pit through the primitive choanal tube (Fig. 3D). The primitive choanal tube is higher than at the previous stage, and its anterior part may be considered as a primordium of the vestibulum. At this time it starts to be filled by the nasal plug containing cells with spherical nuclei (Fig. 3B), but the cylindrical cells of epithelial edges of the nasal prominences are distinguishable (Fig. 3B). The distribution of the presumptive sensory olfactory epithelium seems to be restricted to the Stammteil and the anterior part of the extraconchal space. It correlates with the part of the nasal pit characterized by higher X-ray density, but the vestibular part of the primitive choanal tube, filled by the nasal plug, is also relatively dense at this stage (Fig. 3C”, D’).

**Fig. 4:**
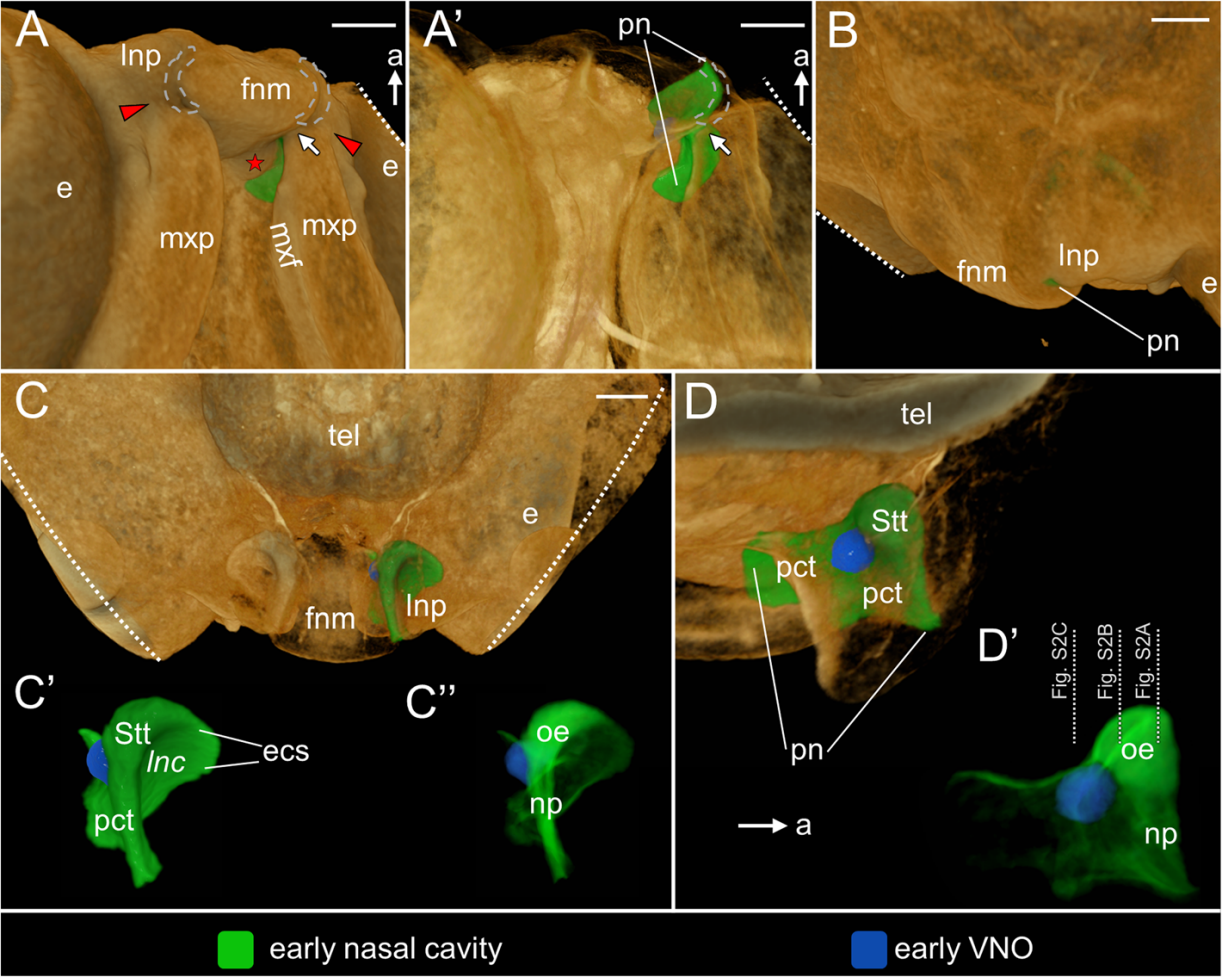
The naso-palatal complex in the brown anole at stage 7. **A** Ventrolateral view of the palate. **A’** Ventrolateral view of the palate (semi-transparent). **B** Anterolateral view of the snout. **C** Anterior view of the semi-transparent snout. **C′** Zoomed anterior view of the left early nasal cavity. **C”** Zoomed anterior view of the left early nasal cavity rendered with partial opacity. **D** Sagittal cutaway of the semi-transparent snout showing the medial view of the left early nasal cavity. **D’** Zoomed medial view of the left early nasal cavity rendered with partial opacity. Abbreviations: *a* anterior, *e* eye, *ecs* extraconchal space, *fnm* frontonasal mass, *lnc* lateral nasal concha, *lnp* lateral nasal prominence, *mxf* maxillary fold, *mxp* maxillary prominence, *np* nasal plug, *oe* olfactory sensory epithelium, *pct* primitive choanal tube, *pn* primitive naris, *Stt* Stammteil, *tel* telencephalon. *Red star* primordium of the vomerine cushion, *red arrowhead* nasolacrimal groove, *single white arrow* approaching of the maxillary prominence to the frontonasal mass, *grey dashed line* presumptive external naris. Note: italicized label on 3D image (*lnc*) indicates the concavity in the early nasal cavity. Scale bars 200 μm

At **stage 7**, the medial nasal prominences are merged and form a single median structure called the frontonasal mass (Fig. 4A). The posterior extensions of the medial nasal prominences, forming the paired primordium of the anterior segment of the palate, become almost obliterated. The lateral nasal prominence and the frontonasal mass approach closely to each other through their entire lengths, reducing the anterior part of the primitive naris to a narrow slit (grey dashed lines in Figs. 4A and A’). This part of the primitive naris marks the presumptive external naris, which is directed ventrally (Fig. 4B). Although fusion between the medial nasal prominence of the frontonasal mass and lateral nasal prominence does not occur, the anteriormost part of the primitive naris is non-patent, because it is filled by the nasal plug (Fig. S2A). Medial to the nasolacrimal groove (red arrowhead) and ventral to the primordium of the vomerine cushion, the maxillary prominence approaches closely the frontonasal mass (white arrow in Fig. 4A and A’). The posterior half of the primitive naris, located behind the approaching tips of the maxillary prominence and the frontonasal mass, widens gradually (Fig. 4A, A’). Posterior to the primitive naris, the maxillary fold, emerging from the lateral surface of the maxillary prominence, is visible (Fig. 4A).

The growth of the facial prominences causes that the primordium of the vestibulum and the remaining part of the primitive choanal tube become deeper and more distinct than at stage 6 (Fig. 4C–D’). Thus, the early VNO increases the distance to the primitive naris (Fig. 4D). At this developmental stage the extraconchal space is much more distinct (Fig. 4C, C’ and see Fig. S2B). Due to the significant changes in the shape of the nasal pit at stage 7 it may be considered as an early nasal cavity. There are no significant changes in the distribution of the presumptive sensory olfactory epithelium (Fig. 4C”, D’). In the sensory olfactory and vomeronasal sensory epithelium, the division into the basal cells (L1) with spherical nuclei and central layer (L2) of cells with slightly more darkly stained and elongated nuclei is relatively weakly visible (Fig. S2A–C). The most apical part of the epithelium (L3) is very thin and the brightest layer. It contains only a few nuclei, usually undergoing mitosis (Fig. S2A–C).

**Fig. 5:**
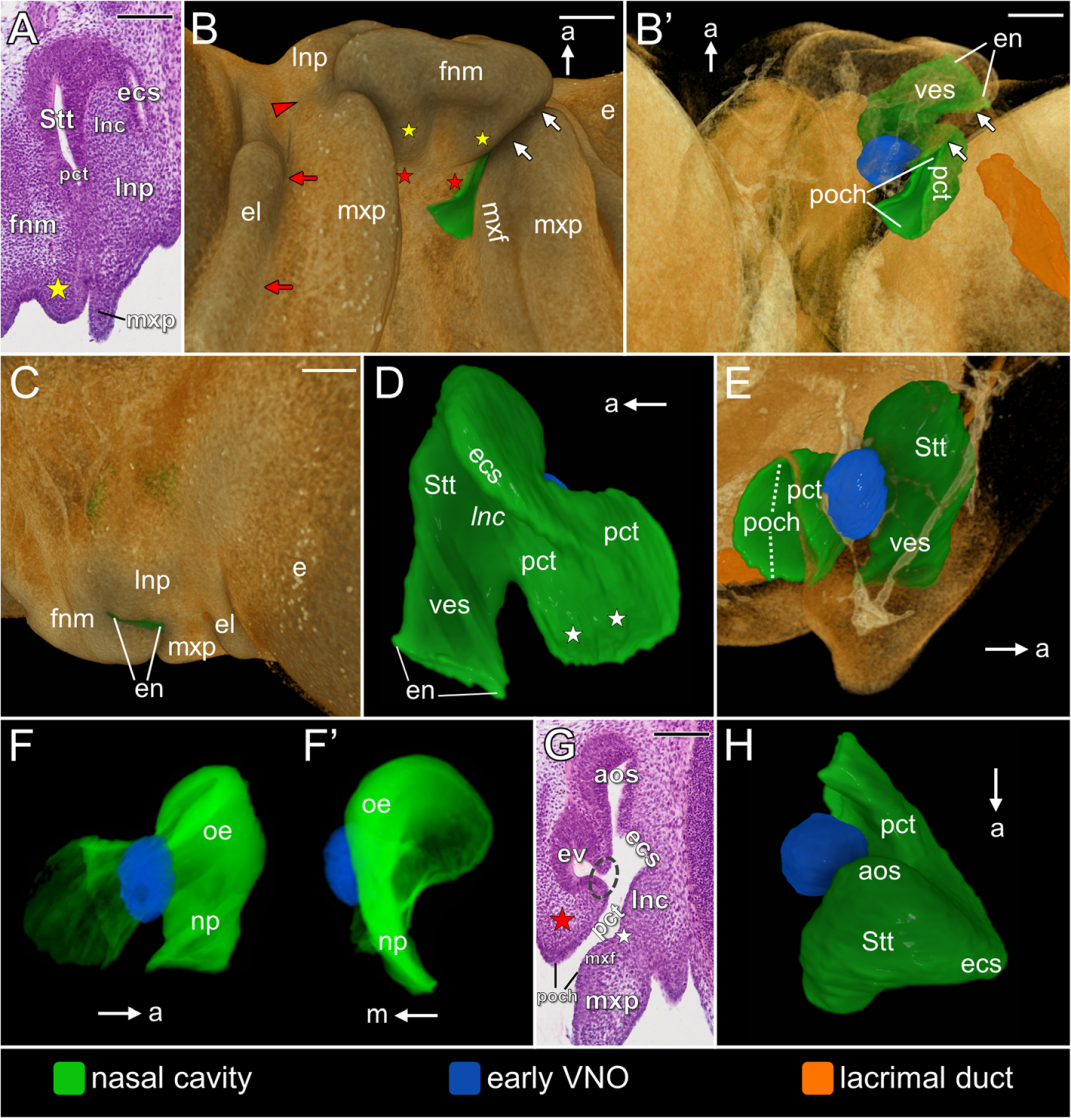
The naso-palatal complex in the brown anole at stages 8 based on the transverse histological sections (**A**, **G**) and 3D reconstructions (**B**–**F′** and **H**). **A** Section just posterior to the vestibulum. **B** Ventrolateral view of the palate. **B′** Ventrolateral view of palate (semi-transparent). **C** Anterolateral view of the snout. **D** Lateral view of the left nasal cavity. **E** Sagittal cutaway of the semi-transparent snout showing the medial view of the left nasal cavity. **F**, **F′** Nasal cavity rendered with partial opacity: medial (**F**) and anterior (**F′**) views. **G** Section at the level of the early VNO. **H** Dorsal view of the left nasal cavity. Abbreviations: *a* anterior, *aos* antorbital space, *e* eye, *ecs* extraconchal space, *el* lower eyelid, *en* external naris, *ev* early VNO, *fnm* frontonasal mass, *lnc* lateral nasal concha, *lnp* lateral nasal prominence, *m* medial, *mxf* maxillary fold, *mxp* maxillary prominence, *np* nasal plug, *oe* sensory olfactory epithelium, *pct* primitive choanal tube, *poch* primitive outer choana, *Stt* Stammteil, *ves* vestibulum. *Red star* primordium of the vomerine cushion, *yellow star* primordium of the anterior segment of the palate, *white star* choanal diverticulum, *red arrowhead* nasolacrimal groove, *red arrows* groove between the lower eyelid and the maxillary prominence, *two white arrows* fusion between the maxillary and lateral nasal prominence with the frontonasal mass, *dashed ellipse* entrance of the early VNO to the primitive choanal tube. Note: italicized label on 3D image (*lnc*) indicates the concavity in the nasal cavity. Scale bars 200 μm (3D) and 100 μm (histological sections)

At **stage 8** the fusion of the maxillary prominence and lateral nasal prominence with the frontonasal mass (including a part of the primordial anterior segment of the palate) divides the primitive naris into the external naris and the primitive outer choana (Fig. 5A–B’ and Movie S1: 12 s–16 s). Below the fusion, a relatively deep groove between fused prominences can be observed (two white arrows in Fig. 5B and B’). The elongated external naris is bounded by the lateral nasal prominence and frontonasal mass (Fig. 5C). Due to the significant ventral growth of the frontonasal mass, the external naris takes a lateral position in comparison to the presumptive external naris, which is directed ventrally at the previous stage (Fig. 5C and compare with Fig. 4B). At the same time the lateral nasal prominence takes a dorsal rather than a lateral position in relation to the external naris (Fig. 5C). The nasolacrimal groove (red arrowhead) passes into the much longer groove existing between the developing lower eyelid and the maxillary prominence (red arrows in Fig. 5B). The paired primordium of the anterior segment of the palate is visible again (yellow stars in Fig. 5B). Between the ventrolateral ridge of the vomerine cushion primordium and the maxillary fold, the primitive outer choana is located (Fig. 5B, B’). It is a relatively wide opening, but it gradually narrows anteriorly (Fig. 5B’).

The vestibulum is well defined and separated from the posterior part of the primitive choanal tube (Fig. 5D, E) due to the mentioned fusion of the maxillary prominence and lateral nasal prominence with the frontonasal mass. The external naris as the entire vestibulum is still sealed by the nasal plug (Fig. 5F, F’).

The part of the primitive choanal tube, located just dorsal to the fusion becomes incorporated into the primordium of the main nasal cavity (Fig. 5A). The main nasal cavity primordium passes into the posterior part of the primitive choanal tube (Fig. 5D, E). From this stage, the primordium of the main nasal cavity is well defined (Fig. 5D–F), but the extraconchal space is restricted approximately to the posterior half of this part of the nasal cavity (Fig. 5D). The extraconchal space is strongly developed there and extends well ventrally, demarcating the lateral border of the lateral nasal concha, which emerges from the lateral nasal prominence (Fig. 5A). A small antorbital space may be distinguished in the primordial main nasal cavity, and it is located just posterior to the lateral nasal concha (Fig. 5G, H).

The sensory olfactory epithelium seems to be restricted to the Stammteil and the medial wall of the antorbital space (Fig. 5F–G). The connection between the ellipsoidal early VNO and the primitive choanal tube is narrowed (dashed ellipse in Fig. 5G). Ventrolateral to the early VNO a relatively shallow choanal diverticulum of the primitive choanal tube may be distinguished. It is located between the maxillary fold and the base of the lateral nasal concha (white star in Fig. 5D and G).

**Fig. 6:**
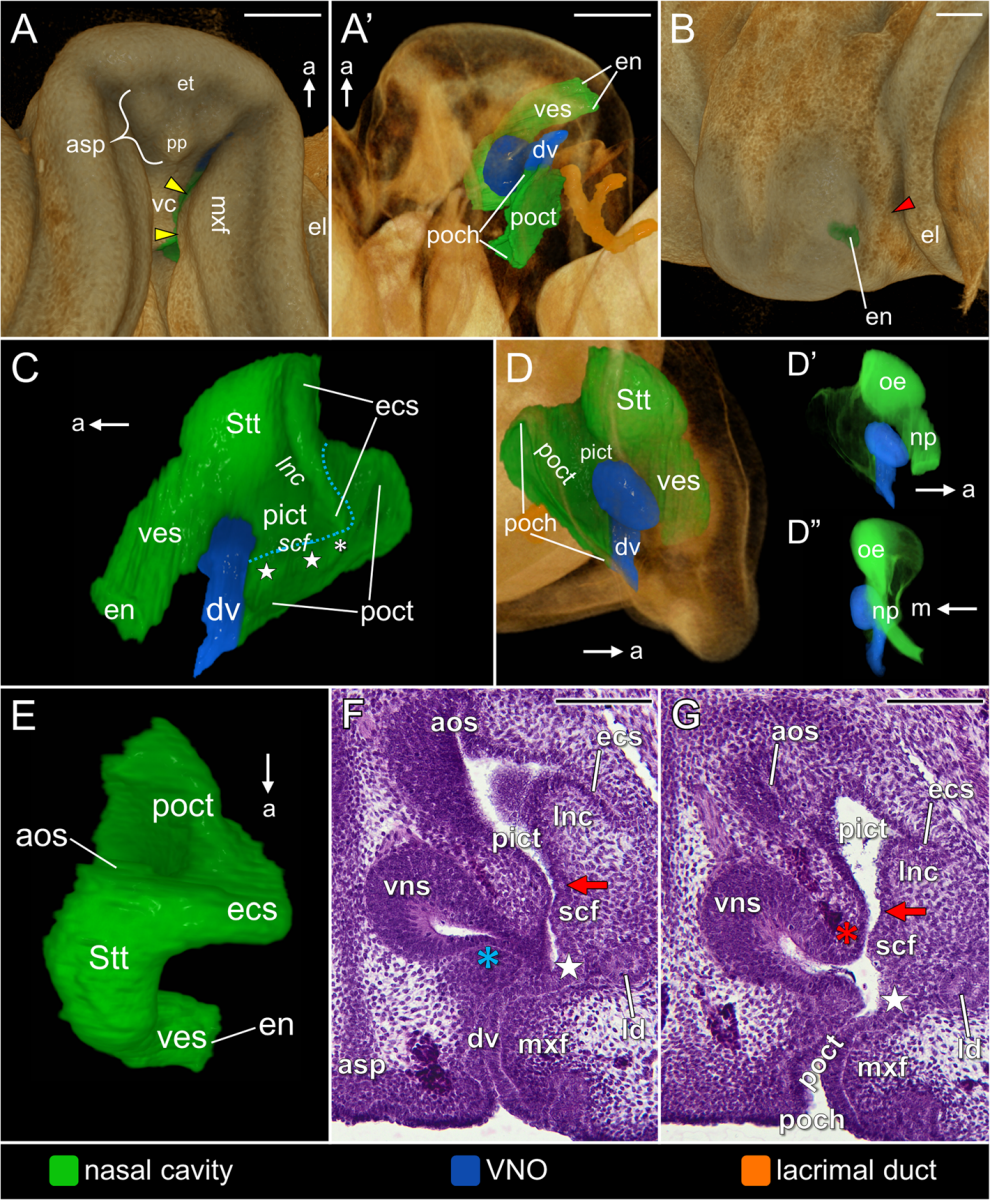
The naso-palatal complex in the brown anole at stage 9 based on the 3D reconstructions (**A**–**E**) and transverse histological sections (**F**, **G**). **A** Ventrolateral view of the palate. **A’** Ventrolateral view of the palate (semi-transparent). **B** Anterolateral view of the snout. **C** Lateral view of the left nasal cavity. **D** Sagittal cutaway of the semi-transparent snout showing the medial view of the left nasal cavity. **D’**, **D’** Nasal cavity rendered with partial opacity to show the sensory olfactory epithelium: medial (**D’**) and anterior (**D”**) views. **E** Dorsal view of the left nasal cavity. **F** Section at the level of the primordial VNO duct. **G** Section at the level of the primitive outer choana. Note that sections **F** and **G** runs slightly obliquely from antorbital space to the VNO duct. Abbreviations: *a* anterior, *aos* antorbital space, *asp* anterior segment of the palate, *dv* duct of the VNO, *ecs* extraconchal space, *el* lower eyelid, *en* external naris, *et* egg tooth, *ld* lacrimal duct, *lnc* lateral nasal concha, *m* medial, *mxf* maxillary fold, *np* nasal plug, *oe* sensory olfactory epithelium, *pict* primitive inner choanal tube*, poch* primitive outer choana, *poct* primitive outer choanal tube, *pp* premaxillary papilla, *scf* subconchal fold, *Stt* Stammteil, *vc* vomerine cushion, *ves* vestibulum, *vns* vomeronasal sensory epithelium. *Red arrowhead* nasolacrimal groove, *yellow arrowheads* choanal fold, *red arrow* lateral expansion of the primitive inner choanal tube, *white star* choanal diverticulum, *red asterisk* ridge of the vomerine cushion, *white asterisk* connection of the choanal fold with the lateral border of the extraconchal space, *blue asterisk* primordial mushroom body, *blue dashed line* localization of the primitive inner choana*.* Note: italicized labels on 3D images indicate the concavities in rendered structures. Scale bars 200 μm (3D) and 100 μm (histological sections)

At developmental **stage 9** the maxillary prominences and the frontonasal mass are almost completely fused and form the upper lip (Fig. 6A). The well-developed anterior segment of the palate is formed by the merging of both components of its paired primordium. The premaxillary papilla is visible at the posterior edge of the segment (Fig. 6A). The maxillary fold closely approaches the anterior segment of the palate and together they form the walls of the non-patent primordial duct of the VNO (Fig. 6A, A’). Posterior to the primordial VNO duct, the primitive outer choanae of both sides demarcate the lateral borders of the elongated vomerine cushion (Fig. 6A). At this stage, the choanal fold (yellow arrowheads) emerges from the maxillary fold (Fig. 6A). It narrows the anterior part of the primitive outer choana, but its posteriormost part remains widely open to the oral cavity (Fig. 6A, A’). The external naris is well established on the lateral side of the snout (Fig. 6B). The nasolacrimal groove is hardly distinguishable (red arrowhead in Fig. 6B).

The vestibulum takes a more tubular form, but it is still oriented more vertically rather than horizontally (Fig. 6C, D) and it remains sealed by the nasal plug (Fig. 6D’, D”). In some specimens of this stage the small primordium of the lateral nasal gland was present, emerging from the vestibulum as an initial bud, just anterior to the anterior end of the primitive inner choanal tube.

The extraconchal space attains a clearly vertical orientation in relation to the antero-posterior axis of the snout and becomes restricted to the posterior third of the main nasal cavity primordium (Fig. 6C, E). The ridge formed by the lateral border of the extraconchal space is confluent ventrally with the posterior part of the choanal diverticulum (white asterisks in Fig. 6C). The antorbital space becomes less distinct (Fig. 6E). The distribution of the sensory olfactory epithelium seems to be the same as previously (Fig. 6D’, D” and F, G). The subconchal fold (= “*Falte*” [13] or “*Lippe am Choanengang”* [65]) becomes distinguishable from the lateral nasal concha. It is restricted ventrally by the choanal diverticulum (white star) and dorsally by the lateral expansion of the primitive inner choanal tube (red arrow in Fig. 6F and G; and see below).

The primitive choanal tube is significantly drawn out posteriorly beyond the level of the antorbital and extraconchal space (Fig. 6D, E). The posterior extension of the primitive choanal tube together with the ventral part of this structure located below the subconchal fold can now be called the primitive outer choanal tube (Fig. 6C). The rest of the primitive choanal tube, which is a ventral part of the primordial main nasal cavity, can now be called the primitive inner choanal tube. The primitive inner choana is located between the primordial main nasal cavity and the primitive outer choanal tube. The anterior and, at the same time, more horizontal part of the primitive inner choana (more horizontal part of blue dashed line in Fig. 6C) is defined by the subconchal fold and the lateral ridge of the vomerine cushion (red asterisks in Fig. 6G). The posterior part of the primitive inner choana takes a vertical orientation. It is located behind the extraconchal space (more vertical part of blue dashed line in Fig. 6C).

At this developmental stage, the VNO is still connected to the nasal cavity. It is open on the medial wall of the primitive outer choanal tube just ventral to the primitive inner choana and medial to the choanal diverticulum (Fig. 6F and G). The primordial VNO duct can be distinguished from the anteriormost part of the primitive outer choanal tube (Fig. 6C–D” and F). At this time the close apposition of the maxillary fold and the anterior segment of the palate reduces the lumen of the primordial VNO duct, which is filled by a narrow cellular plug (Fig. 6F). In the anterior part of the VNO, the small anlage of the mushroom body is observed (blue asterisks in Fig. 6F). It emerges from the floor of the VNO lumen. It is covered by much thinner epithelium than the sensory epithelium of the dorsal dome (Fig. 6F).

**Fig. 7:**
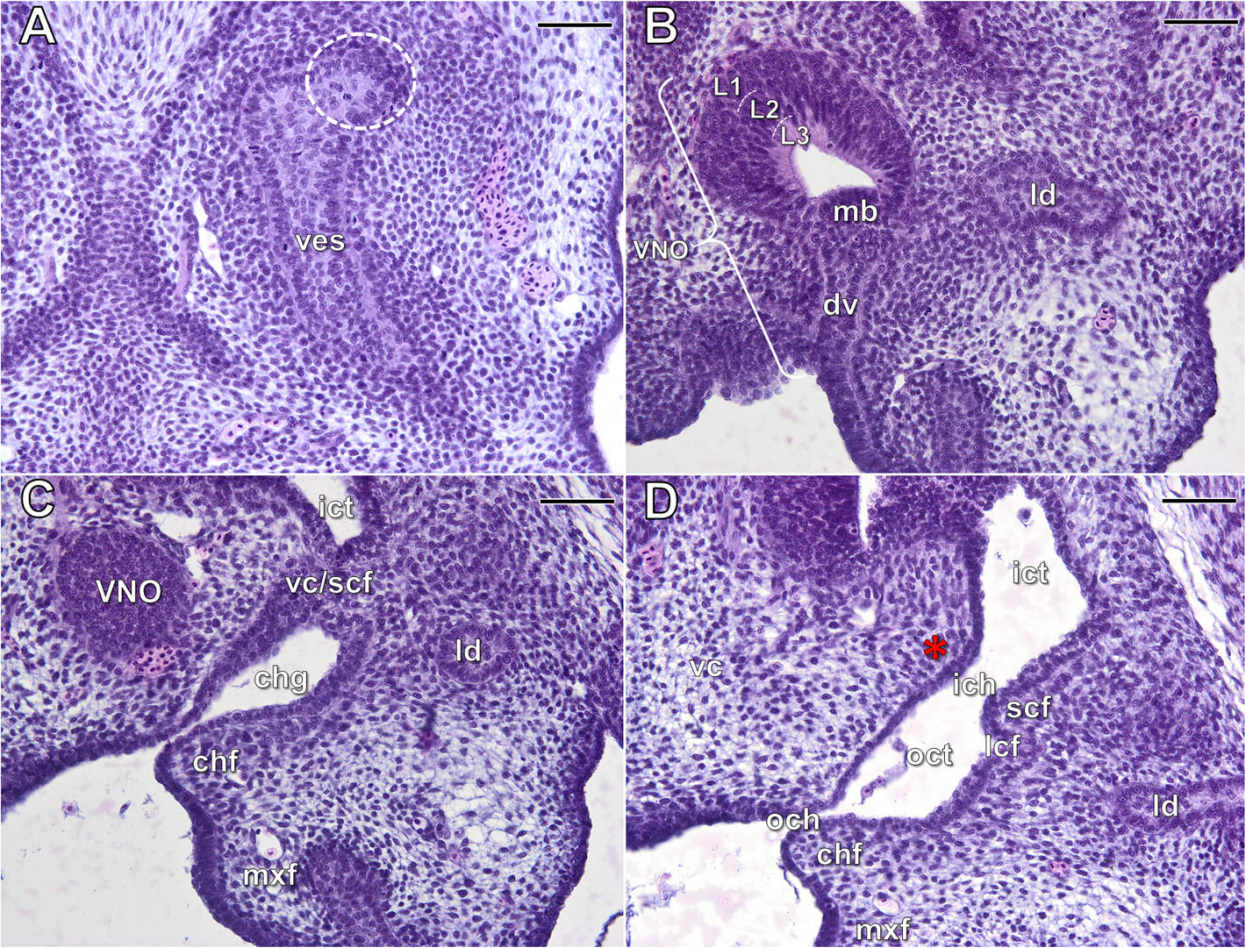
The naso-palatal complex of the brown anole at stage 9/10 based on transverse histological sections through the: vestibulum (**a**), VNO (**b**), choanal groove (**c**) and the outer choana (**d**). Abbreviations: *chf* choanal fold, *chg* choanal groove, *dv* duct of the VNO, *ich* inner choana, *ict* inner choanal tube, *L1***–***L3* layers of the vomeronasal sensory epithelium, *lcf* lateral choanal fissure, *ld* lacrimal duct, *mb* mushroom body, *mxf* maxillary fold, *och* outer choana, *oct* outer choanal tube, *scf* subconchal fold, *vc* vomerine cushion, *vc/scf* fusion of the vomerine cushion and the subconchal fold, *ves* vestibulum. *Dashed circle* primordium of the lateral nasal gland, *red asterisk* ridge of the vomerine cushion. Scale bars 50 μm

At **stages 9/10** and **10** the bud of the lateral nasal gland, emerging from the posterior part of the vestibulum, increases in size and thus becomes more distinct (dashed circle in Fig. 7a). The entire nasal cavity takes a form similar to that observed at the beginning of the next developmental phase. The differentiating mushroom body, reducing the lumen of the VNO, is well visible at these stages, but it is not strongly developed (Fig. 7b).

At stage 9/10 the fusion of the subconchal fold with the vomerine cushion (Fig. 7c) separates the duct of the VNO (and thus the entire VNO) from the nasal cavity, but an indirect connection is still present through the choanal groove (Fig. 7b–d). The choanal groove represents the anterior remnant of the primitive outer choanal tube, which is located below the fusion and now separated dorsally from the main nasal cavity. The choanal diverticulum located below the fusion becomes incorporated into this groove (Fig. 7c). Due to the fusion, the primitive inner choana becomes closed anteriorly. Posteriorly, the remnant of the primitive inner choana (the inner choana) remains open and the subconchal fold approaching the well-developed ridge of vomerine cushion (red asterisk) is visible (Fig. 7d). At the end of this developmental phase (stages 9/10 and 10) the choanal groove is very short. It is bounded medially by the vomerine cushion and laterally by the maxillary part of the palate. The floor of the choanal groove is formed by the choanal fold (Fig. 7c). The posterior part of the choanal groove passes into the outer choanal tube in which the choanal diverticulum is still distinguishable. From this stage it is called at that point a lateral choanal fissure (Fig. 7d). As a result of formation of the choanal groove the primitive outer choana becomes shortened. It is composed of the slit-like anterior part and the posterior part, which is widely open to the oral cavity.

The histology of the sensory epithelia of the nasal cavity and VNO does not exhibit significant changes from that observed at development stage 7 (Fig. 7b).

#### Origin of the lacrimal duct

**Fig. 8:**
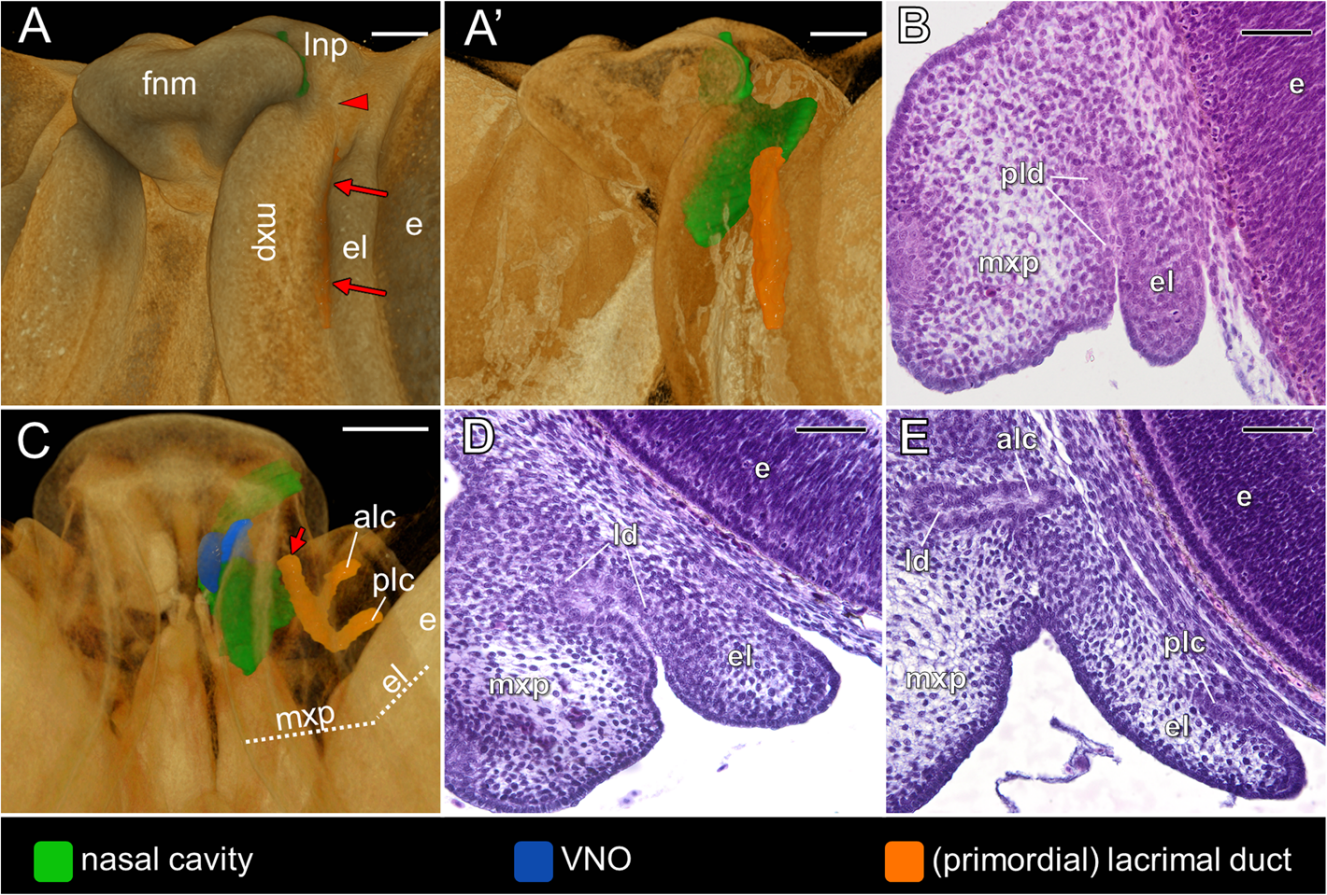
Origin and early differentiation of the lacrimal duct in the brown anole based on 3D reconstructions (**A**, **A’** and **C**) and transverse histological sections (**B**, **D** and **E**). Stage 8: ventrolateral view of the snout (**A**), semi-transparent snout in the same view (**A’**) and section at the level of the primordial lacrimal duct (**B**). Stage 9: ventral view of the semi-transparent palate (**C**) and section at the level of the lacrimal duct “between” the lacrimal canaliculi (**D**). Stage 9/10: section at the level of the lacrimal canaliculi (**E**). Abbreviations: *alc* anterior lacrimal canaliculi, *e* eye, *el* lower eyelid, *fnm* frontonasal mass, *ld* lacrimal duct, *lnp* lateral nasal prominence, *mxp* maxillary prominence, *plc* posterior canaliculi, *pld* primordial lacrimal duct. *Red arrowhead* nasolacrimal groove, *two long red arrows* groove between the lower eyelid and the maxillary prominence, *single short red arrow* freed anterior end of the lacrimal duct. Scale bars 200 μm (3D) and 50 μm (histological sections)

The primordium of the lacrimal duct is first visible at **stage 8**, when the groove between the developing lower eyelid and the maxillary prominence is relatively well distinguishable (two long red arrows in Fig. 8A). The lacrimal duct primordium arises from the deep non-patent part of this groove (Fig. 8A’, B), which is confluent anteriorly with the nasolacrimal groove (red arrowhead in Fig. 8A). At least the posterior part of the nasolacrimal groove seems to also give rise the lacrimal duct (Fig. 8A). In further development (**stage 9**), the primordium of the lacrimal duct attains the form shown in Fig. 8C. Two lacrimal canaliculi depart from it and grow into the tissue of the primordial lower eyelid towards its inner surface. Posteriorly, the lacrimal duct located between the lacrimal canaliculi is still a part of the groove between the developing lower eyelid and the maxillary prominence (Fig. 8C, D). At the level of the connection with anterior canaliculus the lacrimal duct dives into the underlying mesoderm. The freed anterior part of the lacrimal duct (single short red arrow in Fig. 8C) runs anteromedially towards the anteriormost part of the choanal diverticulum, located just posterior to the VNO duct (see Fig. 6F and G). Despite the close apposition of the anterior tip of the primordial lacrimal duct to the choanal diverticulum at stage 9, there is no connection between the lacrimal duct primordium and the choanal groove even at the end of this developmental phase (**stage 10**). The primordium of the lacrimal duct and lacrimal canaliculi are composed of non-patent cellular cord in which a single layer of external cells may be distinguished (Fig. 8E). From stage 9/10 the lacrimal duct becomes completely separated from the groove between the eyelid and the maxillary part of the palate (Fig. 8E). Moreover, the posterior (Fig. 8E) and anterior lacrimal canaliculi clearly reach the inner surface of the eyelid.

At the end of the middle phase of development all structures considered in this study are present, but they do not attain their adult morphology and topographical relationships.

### Late developmental phase (developmental stages 11–19)

#### Main changes at the beginning of the late developmental phase

**Fig. 9:**
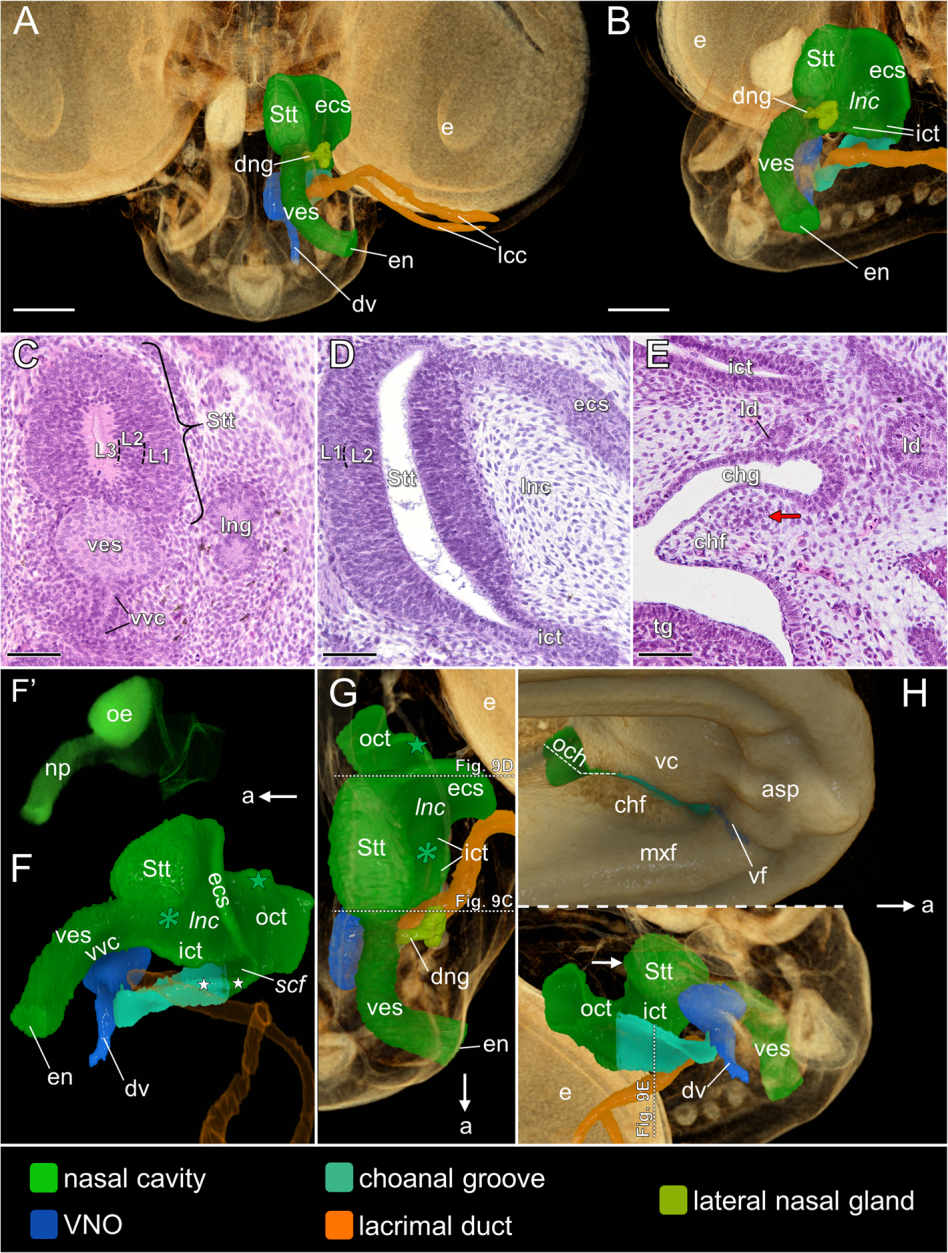
The naso-palatal complex of the brown anole at stages 11 and 12 based on the 3D reconstructions (**A**–**B** and **F**–**H**) and transverse histological sections at levels shown in Figs. G and H (**C**–**E**). **A** Anterodorsal view of the semi-transparent snout; stage 12. **B** Anterodorsolateral view of the semi-transparent snout; stage 12. **C** Section at the level of the connection of the vestibulum with the main nasal cavity; stage 12. **D** Section at the level of the lateral nasal concha; stage 12. **E** Section through the choanal groove; stage 11. **F** Lateral view of the nasal cavity (shown without the lateral nasal gland); stage 12. **F′** Lateral view of the nasal cavity rendered with partial opacity; stage 12. **G** Dorsal view of the nasal cavity; stage 12. **H** Ventrolateral views of the palate; stage 12. Abbreviations: *a* anterior, *asp* anterior segment of the palate, *chf* choanal fold, *chg* choanal groove, *dng* main duct of the lateral nasal gland, *dv* duct of the VNO, *e* eye, *ecs* extraconchal space, *en* external naris, *ict* inner choanal tube, *L1***–***L3* layers of the sensory olfactory epithelium, *lcc* lacrimal canaliculi, *ld* lacrimal duct, *lnc* lateral nasal concha, *lng* lateral nasal gland, *mxf* maxillary fold, *np* nasal plug, *och* outer choana, *oct* outer choanal tube, *oe* sensory olfactory epithelium, *scf* subconchal fold, *Stt* Stammteil, *tg* tongue, *vc* vomerine cushion, *ves* vestibulum, *vf* vomeronasal fenestra, *vvc* vestibular ventral channel. *Green star* narrowed part of the outer choanal tube, *white stars* derivatives of the choanal diverticulum, *green asterisk* anterior extension of the lateral nasal concha, *white arrow* antorbital space, *red arrow* formation of the ectochoanal cartilage. Note: italicized labels on 3D images indicate the concavities in rendered structures. Scale bars 250 μm (**A**, **B**) and 50 μm (**C–E**)

At the beginning of the late developmental phase (stages 11–12) the nasal cavity, lacrimal duct and the choanal groove are well developed (Fig. 9A, B). The vestibulum is elongated and the vestibular ventral channel is well visible (Fig. 9A–C). The other significant change from the end of the previous developmental phase is the presence of a lateral nasal gland in which the primordial main duct may be distinguished (Fig. 9A, B). The contrast in staining between the basal (L1) and central (L2) layers of the olfactory sensory epithelium becomes more visible at stage 12 (Fig. 9C, D). The lacrimal canaliculi extend from two different levels of the lower eyelid and then run medially to form a single lacrimal duct (Fig. 9A). It runs anteromedially from the orbital region towards the VNO, establishing connection with the choanal groove (Fig. 9A, B and E). The VNO is located ventromedial to the posterior part of the vestibulum and only indirectly connected to the nasal cavity through the relatively long choanal groove (Fig. 9B, F).

For better presentation of the changes occurring during the late developmental phase of naso-palatal complex in the brown anole embryos, this section was divided into the following subsections: development of the nasal cavity and the choanal groove, development of the lacrimal duct, and development of the VNO.

#### Nasal and choanal groove, Stages 11–12

*Vestibulum*: At developmental stages 11***–***12, the vestibulum takes a more horizontal orientation than at the previous stage, but its posterior part is still located clearly dorsally in relation to the anterior one (Fig. 9F). The anterior part of the vestibulum turns laterally to achieve the lateral surface of the snout (Fig. 9G). The ventral vestibular channel is restricted to the posterior portion of the vestibulum, and posteriorly it is confluent with the inner choanal tube (Fig. 9F). The lateral nasal gland is composed of the non-patent primordium of the main duct, which is directly connected to the posterior end of the vestibulum, and the spherical bud divided by a few shallow clefts (Fig. 9G).

*Main nasal cavity*:The extraconchal space is restricted to the posterior end of the main nasal cavity (Fig. 9F, G). In this region, the lateral nasal concha is well developed (Fig. 9D). Anterior to the remnant of the extraconchal space, only the anterior extension of the lateral nasal concha can be noted between the Stammteil and the inner choanal tube (green asterisk in Fig. 9F, G).

The inner choanal tube forms distinct lateral expansion. It runs parallel to the diverticulum of the choanal groove and the lateral choanal fissure both originated from the choanal diverticulum (white asterisks in Fig. 9F). The antorbital space is visible as a vertical diverticulum of the main nasal cavity (white arrow in Fig. 9H). The sensory olfactory epithelium is restricted to the Stammteil (Fig. 9C, D and F’) and medial wall of the antorbital space.

*Outer choanal tube and choanal groove*

The posterior half of the outer choanal tube is widely open to the oral cavity through the outer choana (Fig. 9H). The dorsal part of the outer choanal tube is narrowed (green star in Fig. 9F, G). The anterior, slit-like part of the outer choana pass into the slit-like opening of the choanal groove restricted by the well-developed choanal fold and convex vomerine cushion (Fig. 9H). However, the anterior third of the choanal groove, directly confluent with the VNO duct, is more widely open to the oral cavity, due to the fact that the choanal fold does not reach this region (Fig. 9H). On histological sections, there is no sign of well developed cartilaginous support for the choanal fold at this time, but condensation of the mesenchymal cells in the region of future ectochoanal cartilage is visible (red arrow in Fig. 9E).

#### Nasal and choanal groove, Stage 14 (for details see Table 1).

At developmental stage 14, the general morphology of the nasal cavity is very similar to that observed at stage 12, but the lateral nasal gland is better developed (Fig. S3A, B; Table 1). The choanal fold and arched vomerine cushion open widely the anterior portion of the outer choana and the posterior two thirds of the choanal groove to the oral cavity (Fig. S3C, C′). The opening of the anterior third of the choanal groove, located anterior to the choanal fold, becomes narrowed slightly by the vomerine cushion (Fig. S3C, C′).

#### Nasal and choanal groove, Stage 17

**Fig. 10:**
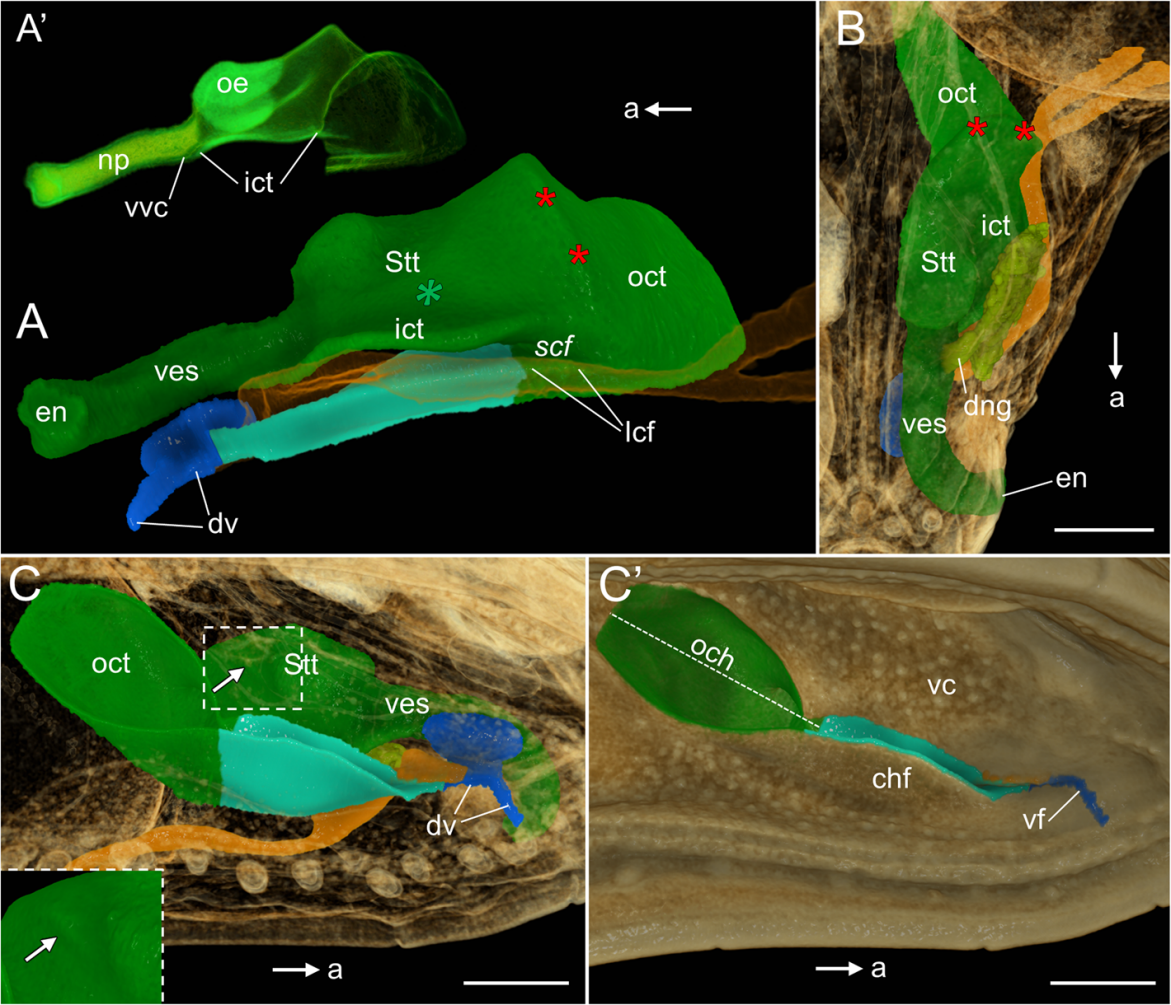
The naso-palatal complex in the brown anole at stage 17. **A** Lateral view of the nasal cavity (shown without the lateral nasal gland). **A’** Lateral view of the nasal cavity rendered with partial opacity. **B** Dorsal view of the nasal cavity. **C** Ventrolateral view of the palate (semi-transparent). **C′** Ventrolateral view of the palate (opaque). Abbreviations: *a* anterior, *chf* choanal fold, *dng* main duct of the lateral nasal gland, *dv* duct of the VNO, *en* external naris, *ict* inner choanal tube, *lcf* lateral choanal fissure, *np* nasal plug, *och* outer choana, *oct* outer choanal tube, *oe* sensory olfactory epithelium, *scf* subconchal fold, *Stt* Stammteil, *vc* vomerine cushion, *ves* vestibulum, *vf* vomeronasal fenestra, *vvc* vestibular ventral channel. *Red asterisks* lateral ridge of the extraconchal space, *green asterisk* anterior extension of the lateral nasal concha, *white arrow* antorbital space. Note: italicized labels on 3D images indicate the concavities in rendered structures. Colours of 3D structures as in Fig. 9. Scale bars 250 μm

*Vestibulum*. At developmental stage 17, the anterior end of the vestibulum is located slightly ventral to the posterior one and thus the vestibulum takes a more vertical orientation than at stage 14 (Fig. 10A). The extension of the ventral vestibular channel becomes reduced to the small posterior remnant just anterior to the inner choanal tube (Fig. 10A’). The nasal plug still fills the entire lumen of the vestibulum (Fig. 10A’). The lateral nasal gland is well developed and according to its shape it may be classified as a compound tubulo-acinar gland (Fig. 10B).

*Main nasal cavity*. The posterior part of the main nasal cavity becomes significantly elongated. The extraconchal space is drawn out posteromedially and dilated (Fig. 10A, B). For this reason, it becomes incorporated to the Stammteil and almost obliterated. Only the “external” ridge on the lateral wall of the main nasal cavity, representing its lateral border, is visible (red asterisks in Fig. 10A, B). Due to the changes in the posterior part of the main nasal cavity, the lateral nasal concha is obliterated in this region. Only the anterior extension of the lateral nasal concha remains as a shallow depression located ventromedial to the lateral nasal gland (green asterisk in Figs. 10A and S4A). The antorbital space forms shallow and elongated diverticulum (white arrow in Fig. 10C). The sensory olfactory epithelium is only present in the anterodorsal part of the main nasal cavity, which constitutes the anterior half of the enlarged Stammteil (including incorporated extraconchal space) or approximately the entire “original” Stammteil (Fig. 10A’).

*Outer choanal tube and choanal groove. *At developmental stage 17 the choanal folds are strongly developed and the choanal groove is still well communicated with the oral cavity (Fig. 10C, C’). The maxillary fold seems to be less distinguishable from the rest of the maxillary part of the palate (Figs. 10C’ and S4A). The outer choanal tube is strongly developed and widely open to the oral cavity (Fig. 10C, C’). The dorsal part of the outer choanal tube, previously narrowed, is now dilated. For this reason, it is visible from the ventral side as a large pit in the palate (Figs. 10C, C’). On the histological sections the well-developed ectochoanal cartilage is visible. It supports the choanal fold through almost all the entire length (Fig. S4A, B), except the region of the lateral choanal fissure.

#### Nasal cavity and choanal groove, Adult-like and adult conditions (stages 18 and 19)

**Fig. 11:**
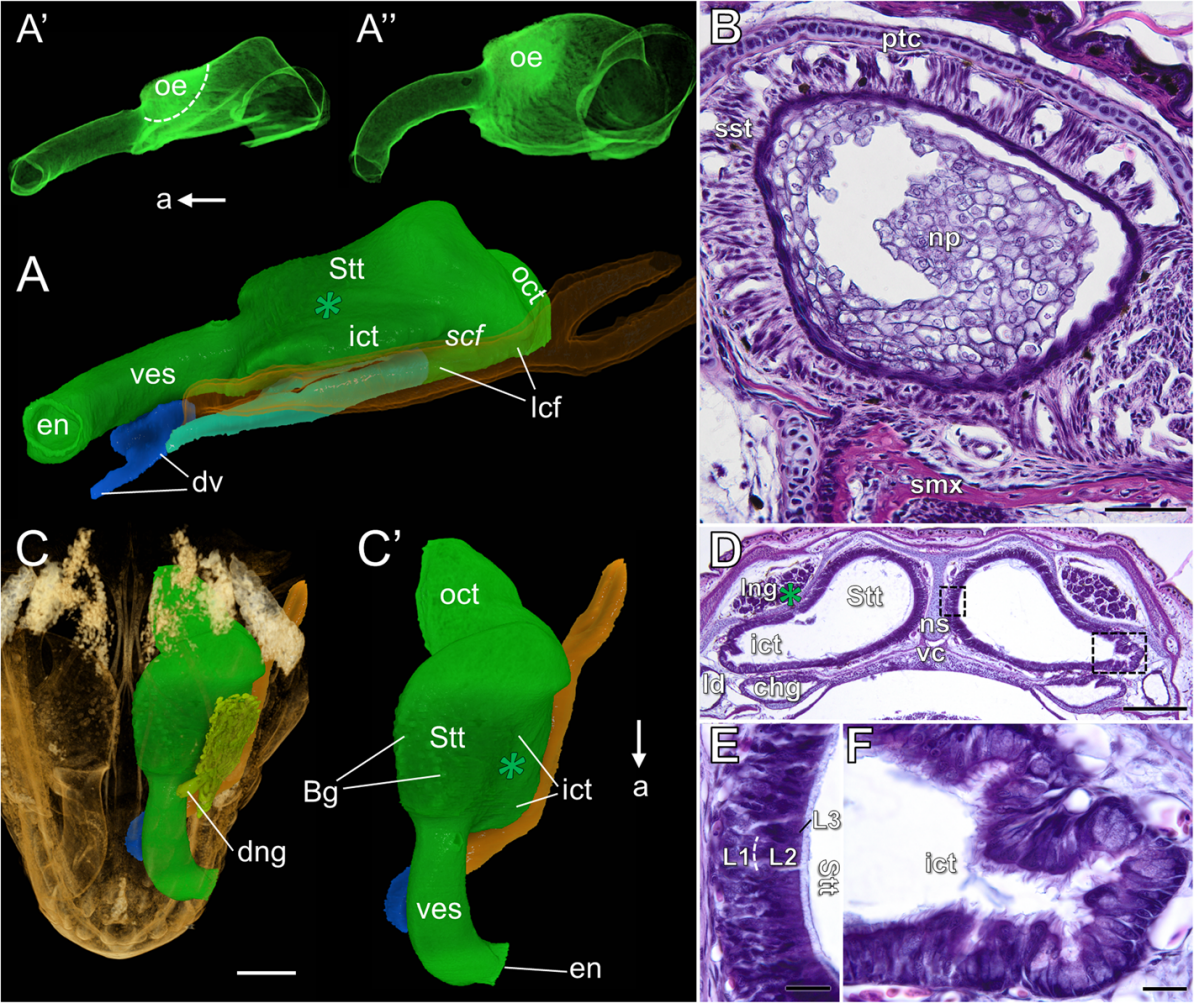
The naso-palatal complex of the brown anole at stages 18 and 19 based on 3D reconstructions (**A**–**A”** and **C**, **C′**) and histological transverse sections (**B** and **D**–**F**). **A** Lateral view of the nasal cavity (shown without the lateral nasal gland); stage 18. **A’**, **A”** Lateral (**A**) and laterodorsal (**A”**) views of the nasal cavity rendered with partial opacity; stage 18. **B** Section through the vestibulum; stage 18. **C** Dorsal view of the snout (semi-transparent); stage 18. **C′** Dorsal view of the nasal cavity (shown without the lateral nasal gland); stage 18. **D** Section at the level of the closed choanal groove; stage 19. **E** Higher magnification of the smaller box from **D**. **F** Higher magnification of the larger box from **D**. Abbreviations: *a* anterior, *Bg* Bowman’s glands, *chg* choanal groove, *dng* main duct of the lateral nasal gland, *dv* duct of the VNO, *en* external naris, *ict* inner choanal tube, *L1***–***L3* layers of the sensory olfactory epithelium, *lcf* lateral choanal fissure, *ld* lacrimal duct, *lng* lateral nasal gland, *np* nasal plug, *ns* nasal septum, *oct* outer choanal tube, *oe* olfactory epithelium, *ptc* parietotectal cartilage, *scf* subconchal fold, *smx* septomaxilla, *sst* spongy sinusoidal tissue, *Stt* Stammteil, *vc* vomerine cushion, *ves* vestibulum. *Green asterisk* anterior extension of the lateral nasal concha. Note: italicized labels on 3D images indicate the concavities in rendered structures. Colours of 3D structures as in Fig. 9. Scale bars 50 μm (**B**), 350 μm (**C**), 200 μm (**D**), 20 μm (**E** and **F**)

*Vestibulum*. The vestibular ventral channel is almost indistinguishable at stage 18 (Fig. 11A, A’), but it is completely absent at stage 19, similarly as the nasal plug. The vestibulum may be completely patent at stage 18 (Fig. 11A’, A”). However, the nasal plug can still be well developed at this stage or only its remnants may be visible (Fig. 11B). At these stages the vestibulum is surrounded by a “sleeve” containing a well-developed sinusoidal capillary network associated with smooth muscle fibers (spongy sinusoidal tissue) (Fig. 11B). The lateral nasal gland can be classified as compound tubular (Fig. 11C).

*Main nasal cavity*. The lumen of the main nasal cavity increases its volume in such a manner that the entire nasal cavity seems to be dilated in comparison to the previous stage (Fig. 11A and C–D). The lateral edge of the extraconchal space is almost obliterated (Fig. 11A). The antorbital space is no longer visible. The location of the olfactory sensory epithelium is the same as at stage 17. It is marked by relatively high X-ray density (Fig. 11A’, A”) and the Bowman’s glands (Fig. 11C’). The respiratory epithelium also exhibits relatively high X-ray density (Fig. 11A’, A”), which seems to be linked with the presence of well-developed goblet cells. The olfactory sensory epithelium becomes thinner than at previous stages and it is now reduced to a few rows of cells (Fig. 11D, E and compare with Figs. S4A and 9D). The division into the basal layer (L1) containing cells with spherical nuclei and the central layer (L2) containing cells with elongated and slightly more darkly stained nuclei is retained (Fig. 11E). The non-sensory epithelium of the inner choanal tube is ciliated. Moreover, the numerous goblet cells and crypts are visible there (Fig. 11F).

**Fig. 12:**
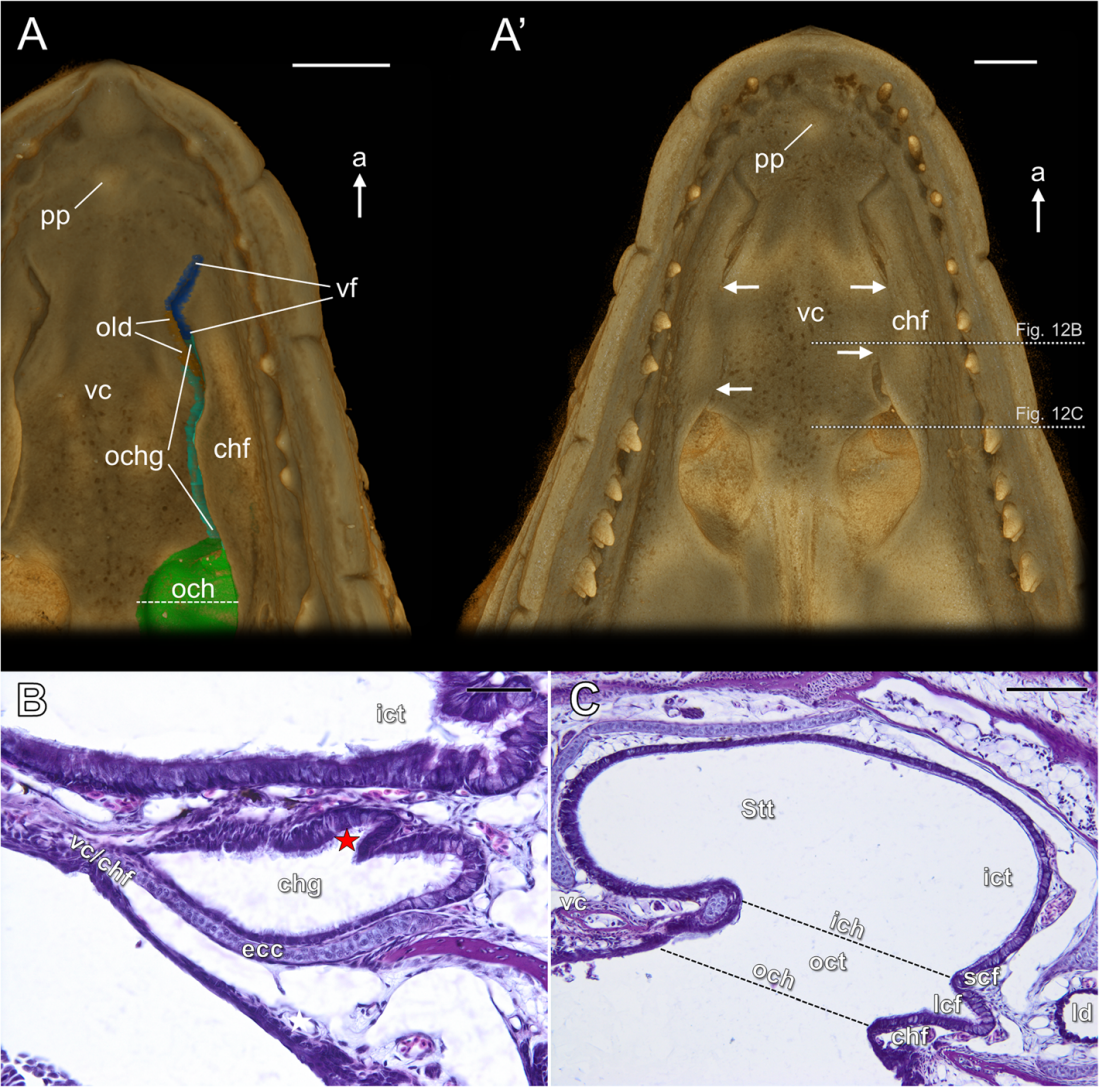
The outer choanal tube and choanal groove at stages 18 and 19 of the brown anole based on 3D reconstructions (**A**, **A’**) and transverse histological sections at the approximated levels shown in Fig. 12A’ (**B**, **C**). **A** Ventral view of the palate; stage 18. **A’** Ventral view of the palate; stage 19. **B** Section through the closed choanal groove; stage 19. **C** Section at the level of the outer choana; stage 19. Abbreviations: *a* anterior, *chf* choanal fold, *chg* choanal groove, *ecc* ectochoanal cartilage, *ich* inner choana, *ict* inner choanal tube*, lcf* lateral choanal fissure, *ld* lacrimal duct, *och* outer choana, *ochg* opening of the choanal groove, *oct* outer choanal tube, *old* opening of the lacrimal duct on the palate, *pp* premaxillary papilla, *scf* subconchal fold, *Stt* Stammteil, *vc* vomerine cushion, *vc/chf* fusion of the vomerine cushion with the choanal fold, *vf* vomeronasal fenestra. *Red star* posterior expansion of the lacrimal duct rostral plate, *white arrows* range of the closed choanal groove. Colours of 3D structures as in Fig. 9. Scale bars 350 μm (**A**, **A’**), 50 μm (**B**), 100 μm (**C**)

*Outer choanal tube and choanal groove*. In all pre-hatched embryos which were studied (stage 18) the choanal groove was open to the oral cavity across its entire length. The opening of this structure was reduced by the choanal fold, which at that time approaches the lateral ridge of the vomerine cushion (Fig. 12A). At developmental stage 19 (newly hatched animal) the choanal groove becomes closed, forming a kind of the duct or channel (white arrows in Fig. 12A’ and B). The fusion of the choanal fold with the vomerine cushion involves at this time most of the length of the choanal groove. It remains open anteriorly, just behind the VNO duct, and posteriorly, just in front of the outer choana, but some asymmetry can be noted (Fig. 12A’). The histological sections revealed that the choanal groove is lined with ciliated epithelium (Fig. 12B), except the part at the entrance to the oral cavity, covered by stratified squamous epithelium. The epithelium which covers the lateral part of the choanal groove contains goblet cells (Fig. 12B). Ciliated non-sensory epithelium containing numerous goblet cells also covers the lateral choanal fissure and the outer choanal tube (Fig. 12C).

#### Development of the lacrimal duct

**Fig. 13:**
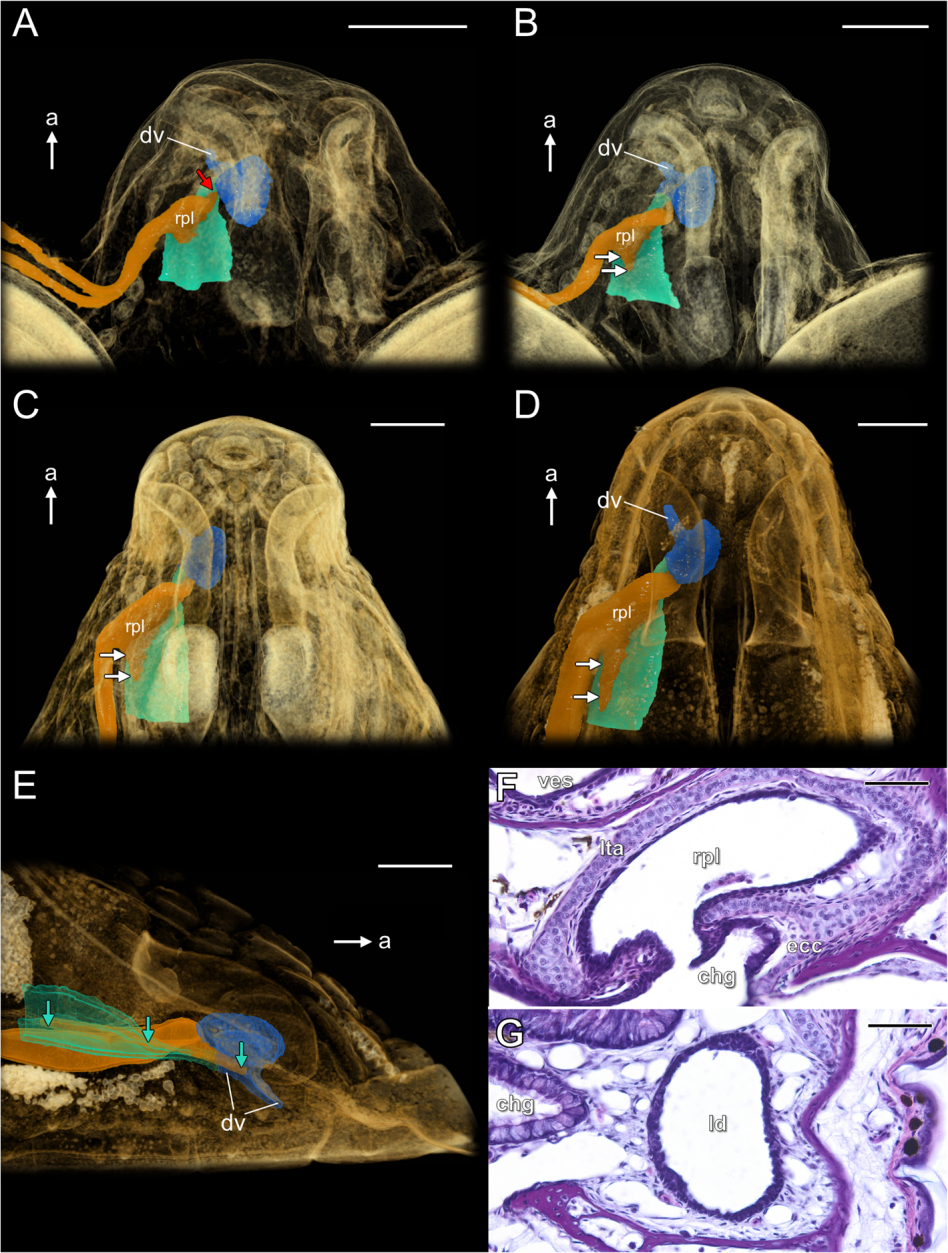
Development of the lacrimal duct at the late developmental phase of the naso-palatal complex in the brown anole based on 3D reconstructions (**a–e**) and transverse histological sections (**f**, **g**): dorsolateral views of the semi-transparent snout at stage 12 (**a**) and 14 (**b**); dorsal view of the semi-transparent snout at stage 17 (**c**) and 18 (**d**); opening of the lacrimal duct to the choanal groove and the VNO duct at stage 18 (**e**); section through the choanal groove, just posterior to the VNO duct; stage 19 (**f**); section through the lacrimal duct at the level of the posterior opening of the choanal groove to the mouth; stage 19 (**g**). Abbreviations: *a* anterior, *chg* choanal groove, *dv* duct of the VNO, *ecc* ectochoanal cartilage, *ld* lacrimal duct, *lta* lamina transversalis anterior, *rpl* rostral plate of the lacrimal duct. *Red arrow* anterior tip of the lacrimal duct, *two white arrows* posterior expansion of the rostral plate of the lacrimal duct, *turquoise arrows* range of the opening of the lacrimal duct to the choanal groove and VNO duct. Colours of 3D structures as in Fig. 9. Scale bars 250 μm (**a–c** and **e**), 350 μm (**d**), 50 μm (**f**, **g**)

At the beginning of the late developmental phase the anterior part of the lacrimal duct forms a plate-like extension, which is connected to the choanal groove in its anterior half (Fig. 13a). The posterior part of this rostral plate is connected to the dorsal part of the choanal groove. Anteriorly, it passes on the medial side of the choanal groove and the rostral tip of the lacrimal duct terminates at a short distance posterior to the VNO duct (red arrow in Fig. 13a). At developmental stage 14, the rostral plate of the lacrimal duct is visible as a triangular-like structure (Fig. 13b). It connects to the anterior two thirds of the choanal groove. The posterior part of the rostral plate of the lacrimal duct forms a characteristic posterior expansion growing towards the inner choana (two white arrows in Fig. 13b). The anterior tip of the lacrimal duct (red arrow in Fig. S3c) terminates at the border between the choanal groove and the VNO duct. At stage 17 the lacrimal duct elongates with the elongation of the entire snout (Fig. 13c). The anteriormost part of the lacrimal duct is clearly connected to the posterior part of the medial wall of the VNO duct and the part of the palate adjacent to it (see Fig. 10C, C’). At developmental stage 18, the connection of the lacrimal duct to the choanal groove becomes more extensive. The posterior expansion of the lacrimal duct rostral plate runs backward toward the choana, but does not reach it (Fig. 13d). Alternatively, at least some considered part of the lacrimal duct expansion (white arrows in Fig. 13d) may constitute the dorsal diverticulum of the choanal groove, since it is covered by ciliated epithelium containing goblet cells (red star in Fig. 12b). The anterior tip of the lacrimal duct is moved more anteriorly along the ventral part of the medial wall of the VNO duct (Fig. 13e). In all but one studied specimens that are at stage 18, the lacrimal duct as well the lacrimal canaliculi were completely patent through their entire length (Fig. 13f, g). Thus a long slit-like opening extends along the contact region between the lacrimal duct and the choanal groove and anteriorly continues as a slit between the lacrimal duct and the VNO duct (turquoise arrows in Fig. 13e).

#### Development of the VNO

**Fig. 14:**
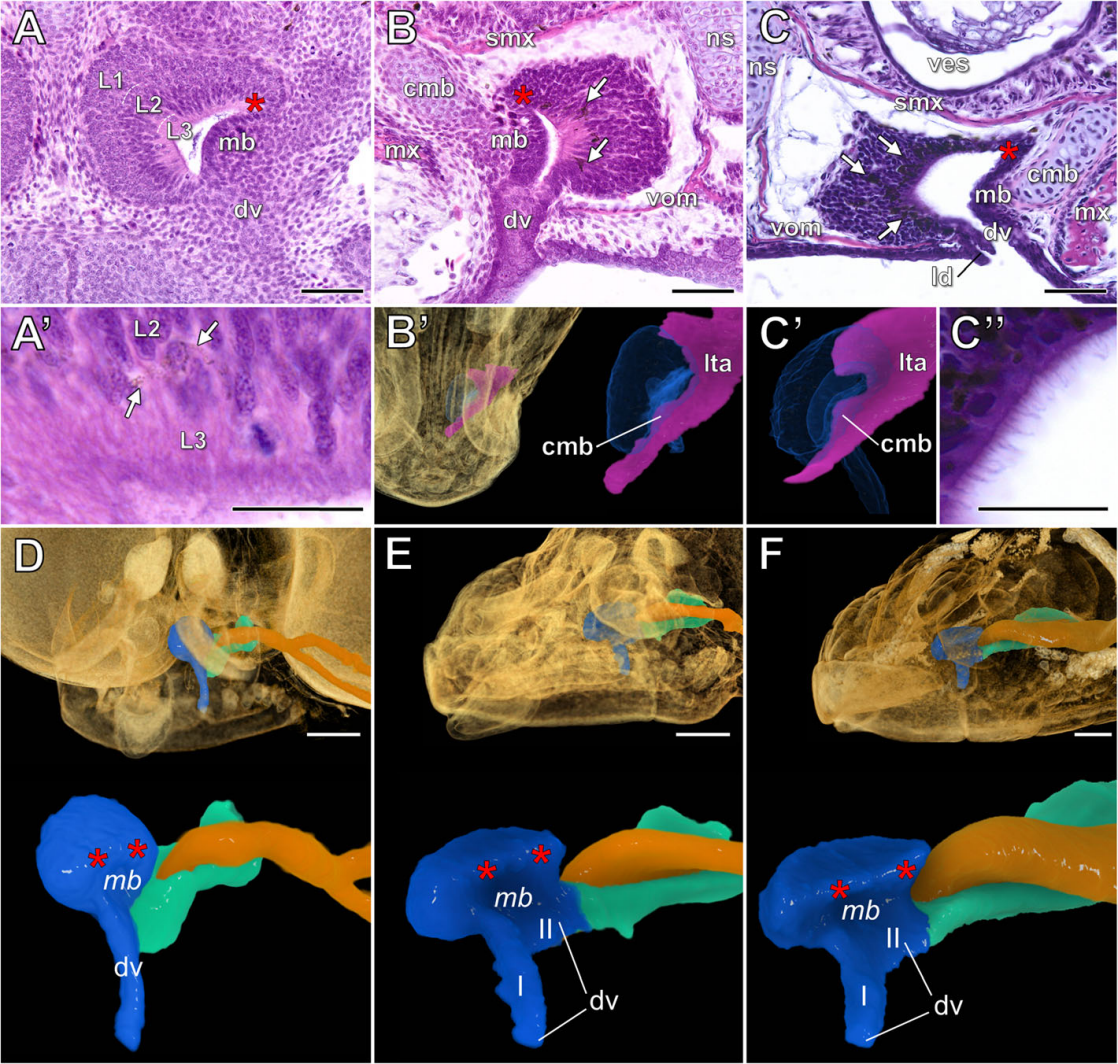
The VNO at the late developmental phase of the naso-palatal complex of the brown anole based on the transverse histological sections (**A–B**, **C** and **C”**) and 3D reconstructions (**B′**, **C′** and **D–F**). **A** Section through the VNO at stage 12. **A’** Section through the vomeronasal sensory epithelium at stage 12. **B** Section through the right VNO at stage 17. **B′** Cartilage of the mushroom body at stage 17. **C** Section through the VNO at stage 18. **C′** Cartilage of the mushroom body at stage 18. **C”** Higher magnification of the apical part of the vomeronasal sensory epithelium, stage 18. **D–F** Anterolateral views of the VNO at stages: 12 (**D**), 17 (**E**) and 18 (**F**). Abbreviations: *cmb* cartilage of the mushroom body, *dv* duct of the VNO, *L1***–***L3* layers of the vomeronasal sensory epithelium, *ld* lacrimal duct, *lta* lamina transversalis anterior, *mb* mushroom body, *mx* maxilla, *ns* nasal septum, *smx* septomaxilla, *ves* vestibulum, *vom* vomer. *White arrows* pigment granules, *red asterisk* ventral channel of the VNO, *I* and *II* parts of the VNO duct. Note: italicized label (*mb*) on 3D images indicates the concavities in VNOs. Violet in **B′** and **C′** is a cartilage and the remaining colours of 3D structures as in Fig. 9. Scale bars 50 μm (**A**, **B**, **C**), 20 μm (**A’** and **C”**) and 250 μm (**D–F**)

At the beginning of the late developmental phase (stages 11–12) the VNO is located ventromedial to the vestibulum of the nasal cavity at the level of its posterior part (Fig. S5A). Later in development (from about stage 17) it is located ventromedial to the middle of the length of the vestibulum (Figs. S5B, C). The main mass of the VNO is formed by thick sensory epithelium, which becomes gradually shifted medially to the VNO duct (Fig. 14A, B and C). Despite the fact that the entire nasal cavity is much larger than the VNO, the difference in size between the sensory part of the nasal cavity and the dorsal dome of the VNO is not very large (Fig. S5A–C). Moreover, at developmental stage 18, the sensory olfactory epithelium is about half as thick as the sensory epithelium of the VNO (compare Fig. 14C and Fig. 11D, E). Similarly, as at the end of the previous developmental phase, the sensory epithelium of the dorsal dome is much thicker than the non-sensory epithelium covering the mushroom body (Fig. 14A, B and C). On the histological sections of VNO from embryos of early developmental phase (stage 12) a few pigment granules in the apical part of the central layer are observed (white arrows in Fig. 14A’). At stages 17–19, the pigment granules are well visible and more frequent in the entire height of the central layer (Fig. 14B, C). At stages 18 and 19, sparsely distributed pigment can be observed also in the basal layer (Fig. 14C). At this time the cells of the vomeronasal sensory epithelium are characterized by hyperchromatic nuclei (Fig. 14C). Thus the contrast in staining between the basal layer (L1) and central layer (L2), which is well defined at the beginning of the late developmental phase (Fig. 14A), is no longer visible at stage 18. It becomes lost about stage 17 (Fig. 14B). On the histological transverse sections of embryos at stage 18, the microvilli resembling the cilia become visible on the surface of the sensory epithelium (Fig. 14C”). At developmental stage 17 the cartilage of the mushroom body is formed. It emerges from the lamina transversalis anterior as a relatively small bulge (Fig. 14B, B’). A similar condition occurs at stage 18 (Fig. 14C, C’). The mushroom body reduces the lumen of the VNO through most stages of the late developmental phase (Fig. 14A, B), but at stage 18 the lumen of the organ is increased significantly (Fig. 14C). Due to the fact that the mushroom body is relatively small and protrudes to the VNO laterally rather than ventrally, the ventral channel is weakly developed and forms the dorsolateral corner of the VNO (red asterisks in Fig. 14A, B, C and D–F). The ellipsoidal-like shape of the VNO, known from the beginning of late developmental phase (Fig. 14D), becomes gradually distorted and the ventral channel becomes more easily visible (Fig. 14E, F). The dorsomedial and dorsal surfaces of the VNO are clearly concave at stage 18 and in the transverse section the organ resembles a rhombus (Fig. 14C). Except for one specimen, the cellular plug sealing the access to the VNO was absent at stage 18 (Fig. 14C). At stage 12, the VNO duct forms a more or less tubular structure located between the anterior segment of the palate and the maxillary fold (Figs. 9H and 14D). In later development the borders of the anterior segment of the palate become obliterated (Fig. S3C’ and see Fig. 12A, A’). With the elongation of the snout and the entire VNO, two parts of the VNO duct may be distinguished: the part located just ventral to the VNO (II), which passes anteriorly into the anterolateral extension (I), well visible at stages 17–18 (Fig. 14E, F). The VNO duct and the ventral channel are located on the opposite sides of the mushroom body (Fig. 14A, B, C and D–F). From about stage 14, the vomer and septomaxilla become visible, and from about stage 17 they are well developed (Fig. 14B, C). The septomaxilla is located dorsal to the VNO, while the vomer is located ventral and ventromedial to the organ. The gap between the vomer and septomaxilla is filled by the nasal septum. The maxilla approaches the lateral side of the VNO, but the lamina transversalis anterior (and the cartilage of the mushroom body) separates these two structures (Fig. 14B, C).

## Discussion

The present study provides the first description of the development of the naso-palatal complex in a member of the Dactyloidae. At the same time it is one of the most comprehensive anatomical descriptions of the squamate naso-palatal complex, taking into account morphogenesis of both the superficial palate and internal structures of the snout, such as the nasal cavity and the VNO. For a better description of the analyzed developmental events we refer to some structures which have previously been overlooked, namely the subconchal fold and the anterior segment of the palate. They are involved in the formation of the choanal groove and the duct of the VNO respectively. Moreover, we propose new terminology for the maxillary fold and different parts of the choanal tube. The former seems to be “forgotten” in contemporary literature.

### Early developmental phase

The paired nasal pits in tetrapods, originating from the nasal placodes, are located on the lateral sides of the developing head. Each of them is bounded by the lateral and medial nasal prominences [6, 18, 21]. The results of our study showed that the nasal pit in the brown anole embryos is present very early, at stage 2. The vomeronasal pit appears at stages 3–5 (Fig. 2F, H and H’), emerging from the medial wall of the nasal pit. The primordial Stammteil, extraconchal space, and choanal tube were distinguishable in the nasal pit at the end of the early developmental phase of the naso-palatal complex. Moreover, the maxillary prominence terminates just posterior to the nasal pit at this time (Fig. 2C).

The beginning of the developmental sequence of analyzed structures in the brown anole (the origin of the vomeronasal pit from preexisting nasal pit, and the later anterior extension of the maxillary prominence) seems to be similar to that described in other Tetrapoda [6, 18, 21].

### Initial fusion and formation of the vestibulum

The medial nasal prominences fuse and form the single frontonasal mass at stage 7 of the brown anole. A similar frontonasal mass is present also in embryos of other squamates, turtles and birds [18, 66–68].

It is generally believed that the fusion of the nasal prominences result in the formation of the vestibulum of the nasal cavity and separation of the external naris from the primitive outer choana [17, 18, 69]. However, the importance of the maxillary prominence in this process may be underestimated since the studies utilizing optical projection tomography (OPT) showed that the fusion between facial prominences, which initiates the formation of the primary palate, varies across amniotes. In turtle (*Emydura subglobosa*) the medial nasal prominence fuses with the lateral nasal prominence, but in chick the initial fusion with the medial nasal prominence involves the maxillary prominence only [67]. In crocodilians and mammals the initial fusion involves all facial prominences [68]. A similar situation was found in our study in the brown anole, despite the significant differences in shape of the facial prominences. Abramyan et al. [67] reported that in lizards (bearded dragon, veiled chameleon, and whiptail lizard), the initiation of the primary palate formation does not engage the maxillary prominence as in turtle, but the external naris becomes closed by the fusion of the nasal prominences and reopens in later development. However, it seems that the complete fusion described by Abramyan et al. [67] in lizards is a combination of two events: the formation of the nasal plug (anteriorly) and the “classical” fusion (posteriorly; if present), where the epithelial seam is replaced by the mesenchymal bridge (see [68, 70]). Thus we are unable to say whether the condition observed by Abramyan et al. [67] in lizards is truly different from that observed by us in the brown anole, in which all facial prominences initiate the fusion of the primary palate. The description provided by Parsons [69] indicates that obliteration of the external naris, and thus formation of the nasal plug, may occur relatively later in development of turtle (*Chrysemys*) than in snakes and other squamates. This is consistent with our observations on the brown anole, since the formation of the nasal plug starts before the initial fusion of the facial prominences and formation of the vestibulum. In fact, at the time of separation of the external naris and the primitive outer choana, which occurs at stage 8 of the brown anole (**Movie S1**), the external naris and the vestibulum are sealed by the well developed nasal plug. Similar “delay” in formation of the nasal plug, which has been noted in turtle, may occur in birds (see [71]). These facts may explain the description of Abramyan et al. [67], who did not find the closed external naris in turtle and chick in stages at the time of initial fusion of facial prominences.

The presence of the embryonic nasal plug is a common transient embryonic feature of amniotes. It has been found in snakes [66, 69, 72], lizards [19, 73], turtle [69], *Sphenodon* [74]*,* birds [71, 72] and mammals [72, 75] including human [76, 77]. The nature of the nasal plug is poorly understood. It is described as mass of epithelial cells [75], mass of isodiametric cells [22], peridermal plug [72, 78], or structure formed by keratinocytes [77]. It has been proposed that it forms between the nasal prominences from the epithelial seam, which does not undergo disintegration [71, 72], or as a proliferation of the “invaginated” oral epithelium [78], which in fact may be considered as walls of the presumptive vestibulum. It seems to us that these explanations are not mutually exclusive. In fact, the presumptive vestibulum containing the nasal plug at stages 6 and 7 of the brown anole (Figs. 3B and S2A) resembles a multilayered medial edge epithelial seam in the human embryonic soft palate (see [79]), although in the anole the basal layer, which forms the walls of the future vestibulum, can easily be distinguished. Distribution of the nasal plug is restricted to the vestibulum in later embryonic life [22, 71, 72].

The functional significance of the nasal plug formation is unknown. Weber [72] concluded that the nasal plug is functionless and, in contrast to the auditory meatal plug, does not protect undifferentiated sensory epithelium. As he stated, the amniotic fluid may still reaches the lumen of the main nasal cavity through the open choana (see also [67]). Moreover, despite the fact that recanalization of the vestibulum takes place shortly before hatching in the brown anole (this study) and in other extant sauropsids [72], in some mammals including human, the reduction of the nasal plug occurs relatively early [71, 72, 76]. Nevertheless, we suspect that the formation of the nasal plug may be crucial for vestibulum formation.

Recent studies on chick have suggested that the canalization of the vestibulum and the external naris is caused by “combination of cellular remodeling, apoptosis, as well as non-apoptotic necrosis” [71]. In the brown anole the vestibulum takes the relatively simple form of a long tube, oriented almost horizontally at late stages (17–19; Figs. 10A, 11A). Elongated vestibulum has been found in the other studies on the brown anole [36], in *Anolis carolinensis* [34], *A. cristatellus*, and possibly in *A. pulchellus*, *A. evermanni*, and *A. stratulus* [35]. In contrast, the vestibulum of *A. roquet* (described there as *A. alligator*) has been found to be short [17]. The shortened vestibulum seems to be present also in *A. equestris* (see Fig. 12B in [6]). As was shown, the morphology of this structure may exhibit some variations in these lizards. The great variation of this structure was also found in other squamates. For instance, a shortened vestibulum occurs in snakes and *Gerrhonotus* (Anguidae), but it may be well developed and take an almost vertical orientation as in *Phrynosoma* [34] (Iguania). It has been suggested that the elongation of the vestibulum in squamates is associated with deserticolous habitats [17] and it has been suspected that such feature as well as “less direct communication with the exterior” may decrease the evaporation rate [34]. It is worth mentioning that the vestibulum of adult squamates excludes inspired foreign bodies, such as soil particles, from the respiratory tract. This is possible due to hyperplasia of its epithelium (which occurs in some forms) and mucoid secretion of the lateral nasal gland, which discharges between the vestibulum and anterior part of the main nasal cavity [5, 18]. The function of cleansing the nose may be improved by the length and shape of the vestibulum, and by the presence of a network of sinusoids associated with the reticulum of smooth muscle fibers (spongy sinusoidal tissue, cavernous tissue) surrounding this part of the nasal cavity, which probably close the vestibulum during intumescence [22, 23, 34].

### Formation of the choanal groove and homology of the choanal folds

The term choanal groove is often used to describe the groove on the palatine bone in the region of the choana [39, 80]. Here we use this term exclusively for a soft tissue structure [5, 17, 22] as defined above. The formation of the choanal groove in squamates is associated with the separation of the VNO from the nasal cavity and separation of the main nasal cavity from the oral cavity [6]. The results of our studies showed that in the brown anole embryos all three events occur due to the closure of the primitive inner choana by the fusion of the subconchal fold with the vomerine cushion. Just prior to formation of the choanal groove, the duct of the VNO can be distinguished. The choanal groove, as well the VNO duct, constitute the persistent portion of the primitive outer choanal tube. Our interpretation differs from the previous descriptions of choanal groove formation which indicated that this structure develops by the fusion of the maxillary prominences (or choanal fold) with the vomerine cushion [6, 22]. It was also suggest that the same fusion event is responsible for the obliteration of the primitive outer choanal tube just posterior to the VNO duct, resulting in the shortening of the adult choanal groove. In fact, the choanal groove is shortened anteriorly in many adult squamates and does not reach the VNO duct [5, 6].

It seems that the formation and the obliteration of the choanal groove constitutes two different processes in squamates. As we showed, in the brown anole the former involves the fusion of the vomerine cushion with the subconchal fold, which originates from the lateral nasal concha. The lateral nasal concha originates in turn from the lateral nasal prominence (see also [81]). With the formation of the upper lip, the lateral nasal prominence and the nasolacrimal groove become hardly distinguishable from the rest of the snout (Fig. 6B). This condition may explain previous misinterpretation about the participation of the maxillary prominence, rather than lateral nasal prominence, in formation of the choanal groove.

Despite the absence of the choanal groove – in most snakes [6, 37, 41] it has been shown to occur in the grass snake embryo (and probably in embryos of other snakes) and extends from the VNO duct to the outer choana [20]. Thus it is reasonable to assume that also in non-ophidian autarchoglossans, in which the choanal groove does not reach the VNO duct [37], the anterior part of the choanal groove becomes obliterated during a separate process. It seems that in contrast to the formation of the choanal groove its obliteration may indeed engage the maxillary prominence (choanal fold), as has been suggested [6, 22].

In Gekkota and Iguania, the choanal groove is confluent with the duct of the VNO. We found that in the late stages of the brown anole the choanal groove extends along most of the length of the main nasal cavity. The outer choana and the outer choanal tube are located well posteriorly (Fig. 11A and 12A, A’). Interestingly, at stage 19 most of the choanal groove was found to be separated from the oral cavity, by the fusion of the medial edge of the choanal fold with the vomerine cushion (Fig. 12A’, B). Our findings agree with the previous description of the brown anole [36]. The closed choanal groove has been noted also in *A. cristatellus* and possibly in *A. pulchellus*, *A. evermanni*, and *A. stratulus* [35]. Bellairs & Boyd [6], who based their description on *A. equestris*, *A. lineatopus* and *A. valencienni*, stated that closure of the choanal groove is restricted to the anterior part of the choanal groove, “a little behind the level of the organ of Jacobson.” As far as we know the formation of the closed channel in a place of choanal groove has never been noted in other squamates and such condition is likely a synapomorphy of Dactyloidae.

The presence of the choanal groove was considered to be a synapomorphy of squamates [41]. In fact, in *Sphenodon*, the only extant member of Rhynchocephalia, commonly considered to be a sister group to Squamata [29, 82–84], the nasal cavity widely communicates with the oral cavity through the long outer choana [5, 17, 18]. Bellairs & Boyd [6] suggested that only a short choanal groove is present anteriorly. However, a long outer choana is also present in many representatives of Iguania [22, 34].

The floor of the choanal groove and the lateral margin of the outer choana is formed by the choanal fold, which is supported by the ectochoanal cartilage [6, 22]. The squamate choanal folds (sometimes called the palatine processes) seem to be considered as a structures homologous to the crocodilian and mammalian palatal shelves (see [3–5]), which in these taxa form “true” secondary palate and well developed nasopharyngeal duct [69, 85, 86]. In mammals the palatal shelves arise as vertically-oriented structures, then become reoriented horizontally and fuse with each other over the tongue to form the secondary palate, which anteriorly fuses to the primary palate and the ventral part of the nasal septum [86, 87]. Crocodilian palatal shelves grow horizontally from the beginning (except their posteriormost parts) and they merge to the nasal septum (anteriorly) and to each other (posteriorly) [85, 88].

A review of the German literature published around 1900 indicates that at least two kinds of medial soft-tissue outgrowths of reptilian maxillary prominence can be distinguished [5, 81, 89]. This is consistent with our results, since we were able to find the maxillary fold and the choanal fold originating from it. Based on comparative embryological data, Fuchs [5] postulated that the squamate choanal folds are not homologous to the mammalian palatal shelves. On the contrary, he suggested that mammalian palatal shelves are homologous to the *mediale Seitenfalten* (here: the maxillary fold). Moreover, it was suggested that the homolog of the choanal fold is present in mammalian embryos. It is located dorsal to the (“secondary”) palatal shelve [5, 81] and may be called as “primitive palatal shelve” (*primitive Gaumenfalte*) [90]. Moreover, Fuchs [5] suggested that crocodilian secondary palate is formed by the homologs of choanal folds not the maxillary folds.

The presence of two rather than one medial soft-tissue outgrowth of the maxillary prominence in reptiles seems to be overlooked in current literature. Alternatively, the choanal fold could be considered as a part of the maxillary fold, since the former seems to emerge from the latter in the brown anole (compere Figs. 5B and 6A). However, both structures are relatively distinct from each other from the beginning of the late developmental phase of the naso-palatal complex (see Fig. 9H).

The initial vertical orientation of the mammalian palatal shelves has been discussed by Ferguson [85, 88, 91]. In fact so-called “palatal shelves” of sauropsids grow horizontally from the beginning. He stated that such condition is associated with the large muscular tongue. The alternative explanation may in fact support the view of Fuchs [5] and the elevation of maxillary folds (*mediale Seitenfalten*) to the horizontal orientation may constitute the mammalian synapomorphy.

In birds the palatal shelves approaches closely to each other, but do not fuse as in crocodilians [92]. Interestingly in turtles the similar medial outgrowths of the maxillary prominence (palatal shelves or alternatively the choanal folds) are not formed in some taxa [93], but the main nasal cavity is well separated from the oral one, probably due to posterior extension of the primary palate [22]. The homology between the amniote medial soft-tissue outgrowths of the maxillary prominence requires confirmation in future comparative studies.

### Outer choanal tube and nasopharyngeal duct

Fuchs [5] recognized two parts (limbs) of the choanal tube: horizontal (“*horizontaler Schenkel des Choanenganges*”) and descending (“*absteigender Schenkel des Choanenganges*”). A similar division was applied by Parsons [22], who distinguished a “more or less horizontal dorsal portion lying ventral to the concha” and a ventral portion which “from near the lateral end of this dorsal portion” “extends ventromedially to the choana”. At the same time, he did not consider the choanal tube as a part of the conchal zone. Bellairs [23] and Bellairs & Boyd [6] distinguished the ventral conchal zone and restricted the term “choanal tube” to the descending limb of the choanal tube sensu Fuchs [5]. In most non-ophidian squamates the horizontal limb of the choanal tube (or ventral conchal zone) leads to the descending limb of the choanal tube or choanal tube sensu Bellairs & Boyd [6] through the inner choana. The descending limb of the choanal tube runs ventromedially and opens to the oral cavity by the outer choana [6, 13, 23]. Here we employed division of the choanal tube into the inner and outer choanal tube (Fig. 1C, D), which correspondence to the two parts of this structure distinguished by Fuchs [5] and Parsons [22].

For embryonic structures we also introduced the term “primitive choanal tube” to define the structure connecting the primordial Stammteil to the oral cavity from about stage 5 of the brown anole (Fig. 2E–H). In later development of this species this structure gives rise to the vestibulum, the primitive outer choanal tube, and the primitive inner choanal tube. The primitive outer choanal tube gives rise to the primordial VNO duct, choanal groove, and the outer choanal tube. With the closure of the part of the primitive inner choana (stage 9/10) and the formation of the choanal groove, the primitive inner choanal tube may be called the inner choanal tube. Because the structure defined here as the outer choanal tube is usually recognized to be homologous to the nasopharyngeal duct of snakes and some lizards [6, 22], we consider that only the inner choanal tube belongs the main nasal cavity. In snakes the outer choanal tube forms the nasopharyngeal duct separated ventrally from the oral cavity by the fusion of the vomerine cushion and choanal fold [22].

It is worth noting that the nasopharyngeal duct of snakes and some lizards is not homologous with the nasopharyngeal duct of mammals and crocodiles [22, 94]. The ophidian nasopharyngeal ducts of both sides join together in their posterior course and enter the oral cavity into the median palatal trough (orbitonasal trough) [6, 18, 20, 22]. In this case the inner and outer choana become widely separated [6]. Some forms of the nasopharyngeal ducts (paired or single median) were also found in non-ophidian squamates (*Xantusia*, *Dibamus* chameleons, some skinks). This structure is formed by the medial extension of choanal folds or elongation of the vomerine cushion [22, 34, 40, 41]. However, in most cases the nasopharyngeal duct of lizards is incomplete ventrally and some authors did not distinguish it as a “snake-like” nasopharyngeal duct [6, 22, 34, 40, 41]. Stebbins [34] stated that in *Anolis carolinensis “*the nasopharyngeal duct situated well posteriorly” is present. It seems that Parsons [22] did not consider the condition of *Anolis* as the presence of the nasopharyngeal duct. Instead he noted that: “the choana is short and well posterior”. A similar interpretation was adopted in this study; thus the outer choanal tube was not considered to form the nasopharyngeal duct in the brown anole.

### Lateral nasal concha and main nasal cavity

The nasal concha constitutes any projection into the nasal cavity of amniotes, but definition of this structure varies among different authors [21]. In most squamates only a single concha is present, called the lateral nasal concha. It forms a space for the lateral nasal gland [18, 22]. The lateral nasal concha of squamates is homologous to the posterior concha of *Sphenodon* [94]. Due to the presence of the concha, 3 parts of the conchal zone of the nasal cavity in squamates can be distinguished: the extraconchal space, the Stammteil, and the choanal tube (the inner choanal tube). The primordium of the lateral nasal concha in the brown anole appears at the end of the early developmental phase (stage 5/6), when it emerges from the lateral nasal prominence (Fig. 2E) and thus before the separation of the external naris and the primitive outer choana at stage 8. The same developmental sequence was described in *Thamnophis* [69].

Previous studies indicated that the lateral nasal concha is absent in anoles [2, 17, 22, 34]. We found that reduction of the concha is associated with the shift of the extraconchal space to the posteriormost part of the main nasal cavity and change of its orientation to vertical position. In later development, the extraconchal space becomes dilated and incorporated into the Stammteil (Fig. 10A, B and Fig. 11A, C). However, we found that even in the latest developmental stages (18–19) of the brown anole the remnant of the concha, called here the anterior extension of the lateral nasal concha, is still noticeable in the region of the lateral nasal gland, between the Stammteil and the inner choanal tube (Fig. 11A, D). It is likely that it undergoes further reduction in later ontogenesis. The reduction or lack of the concha was also found in other members of the Iguania including Acrodonta and Pleurodonta. Within the latter, the lateral nasal concha is absent not only in anoles, but also in the taxa classified as “*Uma*-morphotype” containing: *Uma*, *Callisaurus*, *Holbrookia*, *Sceloporus*, *Uta*, and *Phrynosoma* [34], which all belong to the Phrynosomatidae [95]. Depending on a phylogenetic analysis it could be inferred that the lack of the lateral nasal concha occurred independently in the Phrynosomatidae and the Dactyloidae (see [29]) or the loss of this structure might have taken place in the common ancestor of the Phrynosomatidae and a clade containing Dactyloidae and Polychrotidae (see [96]). Still, little is known about the state of this character in other groups of Pleurodonta. In “*Dipsosaurus*-morphotype” lizards (*Dipsosaurus*, *Iguana*, *Crotaphytus*, *Sauromalus*, *Ctenosaura*) [34], some anguids and in monitor lizards the concha is attached dorsally or dorsolaterally. In such cases the extraconchal space was suggested to be absent, and instead the subconchal recess is distinguishable [22]. However, in another study these two structures were considered to be homologous [23].

### VNO

The primordium of the VNO appears as a vomeronasal pit emerging on the medial wall of the nasal pit. It appears before the fusion of the nasal prominences [18, 69]. The vomeronasal pit is also described in crocodilian and bird embryos, but eventually disappears early in development and the VNO is not present in adult specimens [18]. In crocodilians the vomeronasal pit is no longer noticeable after the fusion of the lateral and medial nasal prominences [69]. In the brown anole, the vomeronasal pit was first found at developmental stages 3–5. Considering the level of association of the VNO with the nasal cavity and degree of its development in adult specimens a few types of the VNO may be distinguished. The VNO of amphibians and turtles is a part of the nasal cavity and the non-sensory part cannot be distinguished [97–99]. The mammalian VNO is connected to the nasal cavity and in some forms also to the oral cavity, and it consists of more or less developed sensory epithelium, which is thicker than the non-sensory one [4, 97]. A non-sensory vestige of the VNO is present in humans and chimpanzees [100], but in some bats the VNO may be rudimentary or even absent [101]. In *Sphenodon* the VNO takes a lens-shape form and there is no mushroom body. The VNO lumen is connected to the anterior part of the nasal cavity near the long outer choana [69, 102]. However, some descriptions suggest that the VNO of this taxon communicates with both the oral cavity and the short choanal groove [6].

In adult squamates the VNO has no direct connection with the nasal cavity and its duct enters the oral cavity exclusively as in most squamate groups or the oral cavity and the choanal groove as in iguanians and gekkotans. In skinks and lacertids the choanal gutter is formed by the close association of the choanal groove with the lacrimal duct and is connected to the VNO duct by the lacrimal component [6]. In the brown anole separation of the VNO from the nasal cavity occurs at developmental stage 9/10. At this stage the mushroom body is well distinguishable, but relatively small (Fig. 7B).

In adult squamates the vomeronasal sensory epithelium is generally strongly developed and the non-sensory epithelium covers the mushroom body [2, 15, 103]. The VNO can be reduced in some arboreal Iguania, especially in chameleons in which the mushroom body or even the entire organ is absent [17, 40, 69]. It was suggested that the anole VNO is: “present but less well-differentiated than in most lizards” [2]. In fact we found that the VNO of the brown anole at stages 18 and 19 possesses a poorly developed mushroom body and weakly developed ventral channel (see Fig. 14**)**. Histological study revealed medial localization of the dorsal dome in relation to VNO duct. Interestingly, the adult vomeronasal sensory epithelium has well-visible pigment aggregated within its central layer (Fig. 14C). Similarly as in brown anole embryos three layers of developing vomeronasal sensory epithelium were also found in other light microscopic studies [2, 18, 20] (e.g. Fig. 14A). The ultrastructural studies indicated that this epithelium contains different populations of cells [16, 103, 104]. Based on previous data and our studies we can suppose that the basal layer (L1) is a population of developing bipolar neurons and undifferentiated cells, the central layer (L2) is composed of supporting cells while the apical one (L3) includes the dendrites of bipolar neurons and protrusions of the supporting cells.

The sensory epithelium of the ophidian VNO is characterized by a peculiar columnar organization [16, 18, 20, 103, 104]. The columns are located basal to the thin layer of the supporting cells, and contain bipolar neurons and undifferentiated cells. Columns extend through most of the height of the entire vomeronasal sensory epithelium. Such columnar compartments are separated by the invaginated basal lamina and connective tissue containing the capillary network. Similar columnar organization may be present in non-ophidian squamates, but they are not such well developed as in snakes [22]. For instance, Kratzing [15] stated that “columns of connective tissue” with a capillary network intrude the sensory epithelium of the VNO in *Tiliqua*, while in *Takydromus* only small, sparsely distributed blood vessels were found in vomeronasal sensory epithelium [105]. The penetration of vomeronasal sensory epithelium by connective tissue, without formation of columns, was found in some mammals: rodents and rabbit [97]. The columns of neurons have never been described for adult anoles. Neither columnar organization of VNO sensory epithelium nor blood vessels within bipolar and undifferentiated cells were detected in the present study.

The only indication of intrusion of connective tissue into the vomeronasal sensory epithelium might be the occurrence of the pigment granules within the layer of supporting cells, especially frequent in late stages of the brown anole (17–19), but such explanation seems to be unlikely. At stages 18 and 19 of the brown anole, fewer pigment granules occurred also within the basal layer containing bipolar neurons. The presence of connective tissue with associated pigment cells at the base of the sensory epithelium as well as between epithelial columns of bipolar neurons was found in newly hatched and adult *Thamnophis* [44]. The presence of pigment cells in the connective tissue around the base of the VNO was noted previously in anoles, as in other squamates [2, 22, 97]. Nevertheless, the lack of even sparsely distributed blood vessels within the bipolar cell layer in the VNO of the brown anoles suggests that the intrusion of connective tissue into the vomeronasal sensory epithelium does not occur. Moreover, the association of such pigmentation with the supporting cell layer suggest that pigment granules observed here are homologous to the olfactory pigment of supporting cells, which has been found in some vertebrates, but usually reported for mammals and birds [106, 107]. It has been suggested that the carotenoid component of such pigment may be involved in olfactory function in terrestrial vertebrates, in which olfactory pigment is present also in cells of Bowman’s glands [107]. Interestingly, we did not observe similar pigmentation in the sensory olfactory epithelium of the brown anole and the pigment granules have not been noted in vomeronasal sensory epithelium in other vertebrates [107].

The mushroom body in squamates [15, 16, 103, 104], the floor of the VNO in *Sphenodon* [17], and the lateral or dorsolateral wall of the organ in mammals [100, 108, 109] are covered with ciliated non-sensory epithelium. In general the luminal surface of mature vomeronasal sensory epithelium in tetrapods is covered with microvilli of dendrites and protrusions of supporting cells, and there are no cilia characteristic for the dendritic surface of olfactory sensory epithelium. We found that in the brown anole at late developmental stages (18 and 19) the apical surface of the vomeronasal sensory epithelium is covered with microvilli resembling the cilia (Fig. 14C”). Similar images from light microscopy have been obtained for *Takydromus* (see Fig. 2 in Saito et al. [105]). Our preliminary studies based on transmission electron microscopy revealed that the apical surface of the vomeronasal sensory epithelium in late stages of the brown anole is covered by long and thin microvilli and only sparsely distributed cilia can be observed (unpublished observations).

### Lacrimal duct

Most tetrapods, except turtles [6] and a few plethodontid salamanders [110], possess a lacrimal duct. In mammals, birds, crocodilians, *Sphenodon* and most amphibians, it extends from the lower eyelid and discharges into the nasal cavity near the external naris or more caudally, closer to the entrance to the VNO [21, 111–115]. In squamates the lacrimal duct is usually directly associated with the VNO [41]. Moreover, in most non-ophidian squamates it is connected with the choanal groove and, in some cases, also with the nasal cavity at the level of the outer choana [6, 22, 23]. Close association of the VNO and the lacrimal duct was also found in the Gymnophiona [21, 116]. It is widely accepted that the lacrimal duct is involved in direct or indirect delivery of secretions of the orbital gland, including the Harderian gland secretion, into the VNO lumen (e.g. [24, 42, 114]), except for crocodilians and birds, in which the VNO is transitory embryonic feature. It means that the secretion of the Harderian gland is involved in vomeronasal chemoreception, rather than eye lubrication [24, 25, 43, 117]. In fact the flow of the Harderian secretion gland or orbital fluid to the VNO was experimentally tested in snakes [118] and frogs [111, 115]. The other studies on snakes found that female pheromones in *Thamnophis* are soluble in the Harderian gland homogenate [9].

It seems that the origin of the lacrimal duct from the nasolacrimal groove (= naso-optic furrow), located between the maxillary prominence and the lateral nasal prominence, is evolutionarily conserved in tetrapods [6, 88, 94, 119]. In the brown anole, the primordium of the lacrimal duct was first present at stage 8. Interestingly, we found that only a small anteriormost part of the duct seems to originate from the nasolacrimal groove. The major part of the duct in the brown anole develops from the groove located between the primordial lower eyelid and the maxillary prominence (Fig. 8A, A’ and Movie S1). Such a peculiar condition could be explained by the fact that the eyes in this species seem to be very large in relation to the snout. In consequence, the developing eye may cover the posterior extent of the lateral nasal prominence and reduces the length of the nasolacrimal groove. This hypothesis needs to be confirmed by comparative morphometric studies, but the occurrence of the huge eyes of the brown anole in relation to the other squamates seems to be very clear (compare Fig. 1, stage 8 in [54], Fig. 4, stage 32 in [120], Fig. 2H in [121], and Fig. 2, stage 4 in [122].

The orbital end of the lacrimal duct of tetrapods usually forms two canaliculi or sometimes one [24, 113, 123]. Unfortunately, many studies concerning development of the nasolacrimal apparatus are based on relatively late developmental stages, when the lacrimal duct is well developed (e.g. [112–114]). Thus, still little is known about development of the lacrimal canaliculi. Here we show that the lacrimal canaliculi of the brown anole become distinguishable just after the formation of single lacrimal duct primordium.

The lacrimal duct of *Sphenodon* discharges exclusively into the nasal cavity [21, 124]. In contrast to that condition, the lacrimal duct of most adult non-ophidian squamates establishes a relatively extensive connection with the choanal groove [6, 35, 125]. Here we show the steps of formation of such extensive connection in the brown anole, which is established by the lacrimal duct through its rostral plate (see Fig. 13a–d). In gekkotans, except pygopodids, the connection of the lacrimal duct and the choanal groove seems to be restricted to the anterior part of the latter [22, 35]. The rostral end of the squamate lacrimal duct is usually connected to the VNO duct or enters the VNO lumen directly in some forms [6]. The lacrimal duct of monitor lizards, pygopodids, some amphisbaenids, and at least one dibamid, reaches the VNO duct directly, and there is no communication with the choanal groove [6]. The same condition occurs in adult snakes in which the choanal groove is absent [6, 20, 118]. Moreover, in pygopodids, amphisbaenids, and snakes, the lacrimal duct establishes a more intimate connection with the duct of the Harderian gland via lacrimal canaliculi or the Harderian gland discharges directly into the lacrimal duct [24, 43].

In the latest developmental stages of the brown anole (18 and 19), the lacrimal duct is not connected to the outer choanal tube, but the posterior expansion of the rostral plate of the lacrimal duct runs backward toward the outer choana (Fig. 13d). This expansion is lined with the epithelium characteristic for the choanal groove (with the presence of cilia and goblet cells) (Fig. 12B); thus it could be considered as a part of the choanal groove rather than the lacrimal duct. In fact Bellairs & Boyd [6] suggested that “transition from stratified to ciliated epithelium” may approximate the border between the fused lacrimal duct and the choanal groove. However “ciliated respiratory type” epithelium was found in the anterior part of the shorter lacrimal duct in monitor lizards [23], goblet cells in the epithelial lining of the *saccus lacrymalis* in the lacrimal duct are present in crocodilians [112], and the goblet cells in addition to cilia have been found in the human lacrimal duct [123]. Moreover, the changes in anatomy of the lacrimal duct observed in successive stages at late developmental phase suggest that the posterior part of the rostral plate of the lacrimal duct gradually grows posteriorly (see Fig. 13a–d) and thus the region under consideration does not represent the part of the choanal groove. If our interpretation is correct, then it could be assumed that the posterior expansion of the rostral plate of the lacrimal duct (white arrows in Fig. 13d) of the brown anole is involved in a different function than the rest of the lacrimal duct, and constitutes a functional extension of the choanal groove.

### Importance of the olfactory system and functional remarks

Pratt [17] suggested that reduction or loss of functionality of olfactory systems in such forms as *Anolis*, arboreal agamids and chameleons corresponds to the “massive orbital development”. The sensory part of the nasal cavity was described as “almost non-sensory”, while the VNO was described as “reduced and completely non-sensory”. The Dactyloidae originates from an arboreal ancestor [33] and most extant species contain toe pads [126] which serve an adhesive function [127]. However, many extant forms are not strictly associated with a fully arboreal lifestyle [30–33]. The brown anole occupies a “trunk-ground” niche [30, 33]. Pratt’s [17] notion about the non-sensory character of the anole VNO was based on the anole described there as *Anolis alligator* (which probably is now *A. roquet* [95] and represents a trunk-crown ecomorph [128, 129]). In fact, behavioral evidences, including that involving tongue extrusion, indicates that the accessory olfactory system may still be important in males of the brown anole for detection of female pheromones [130]. Increases in the tongue flick rates were found in *A. carolinensis* during male-male encounters and in individuals which were transferred to novel habitats and exposed to foliage or air movements [131]. The tongue extrusions were also observed in the field for *A. trinitatis* [132]. The interpretation of such behavior as evidence for the importance of the accessory olfactory system may be problematic. The role of tongue extrusion in anoles, especially substrate licking, may mediate gustation rather than vomeronasal function, since taste buds of the tongue tip are found to be generally abundant in Iguanidae (“present” in *A. carolinensis* and “abundant” in *A. bonairensis*) [133]. However, some findings suggest that lingual gustation in squamates cannot replace vomerolfaction and thus be responsible for chemical discrimination of prey or conspecifics [12].

Morphological studies also showed that the VNO is functional at least in *A. garmani*, *A. grahami* and *A. lineatopus* [2], which are classified as crown-giant, trunk-crown and trunk-ground ecomorphs respectively [33, 128, 129]. Some features of anole adult morphology may constitute adaptations to vomeronasal chemoreception. For instance, the closure of the choanal groove may efficiently deliver the secretion of the Harderian gland to the VNO duct and its vicinity. A similar condition is present in snakes in which the choanal groove is absent, but the anterior end of the lacrimal duct is exclusively connected with the VNO duct [6]. The presence of a functional VNO in anoles representing different ecomorphs (including crown-giants and trunk-crowns) may suggest that Pratt’s [17] interpretation for *A. roquet* was not correct and the arboreal lifestyle in anoles may be associated with the reduction in size of the sensory epithelium of the VNO (and accessory olfactory bulb) rather than with complete loss of its chemosensory function. Moreover, the generalization of Pratt [17] is falsified by the fact that well-developed olfactory systems in arboreal geckoes are present [37, 134].

It was suggested that reduction of olfactory organs in such representatives of Iguania as chameleons and agamas (and probably *Anolis*) is secondary, and the condition of Iguanidae, which are characterized by relatively well-developed chemosensory abilities, is ancestral [37]. In fact, our studies may indirectly confirm this notion at least for Pleurodonta, since the morphology of the adult brown anole probably does not represent the ancestral condition. The loss/significant reduction of the concha, characteristic for *Anolis* and the “*Uma*-morphotype”, but not for “*Dipsosaurus*-morphotype” including Iguanidae*,* seems to be secondary, since at the beginning of the middle developmental phase the concha and extraconchal space were easily distinguishable in the brown anole. Nevertheless, members of the Iguania in contrast to the rest of the squamates (Scleroglossa) are characterized by a less developed VNO [37], lower tongue-flick rate [135] and lower abundance of vomeronasal and olfactory receptor cells [135]. The weakly or moderately developed chemosensory abilities in Iguania are difficult to explain, since the phylogenetic position of this group is still a matter of debate [50]. Such a *Sphenodon*-like condition may be considered either as plesiomorphy according to morphological studies placing the Iguania as sister to the other squamates (e.g. [83]), or reversal according to molecular phylogeny, which suggests the nested position of Iguania (e.g. [28, 82]).

## Conclusions

The present study provides the first detailed description of the development of the naso-palatal complex in a representative of the Dactyloidae. The origin of the VNO and the nasal cavity in the brown anole is the same as in the other tetrapods, while the primordium of the lacrimal duct, at least in the major part, develops beyond the nasolacrimal groove. Despite the peculiar morphology of the adult nasal cavity of the brown anole (significant reduction of the lateral nasal concha and obliteration of the extraconchal space), the typical squamate conditions are present in the middle phase of its development. It is possible that reduction of the concha (and thus obliteration of the extraconchal space) took place in the common ancestor of the Phrynosomatidae and the clade containing the Dactyloidae and Polychrotidae. It seems that both olfactory systems are functional in the brown anole. Some features of adult morphology may even be considered as adaptations to vomeronasal chemoreception. The terminology introduced in this study and description of development provided here may be useful in future investigations of the olfactory structures in other squamates and allow determination of the homology between them and the structures of other taxa.

## Supplementary information

**Additional file 1: Table S1.** Numbers of the brown anole embryos used for light microscopy (LM) and microtomography (XRM).

**Additional file 2: Table S2.** Microtomography scanning parameters. (PDF 91 kB)

**Additional file 3: Fig. S1.** The manner in which the segmentations were performed, illustrated with stage 17 of the brown anole (**A**) and alignment (for description) of the antero-posterior axis for embryos heads (**B**). Abbreviations: *a* anterior, *cmb* cartilage of the mushroom body, *dv* duct of the VNO, *e* eye, *eves* epithelium of the vestibulum, *mes* mesencephalon, *mxp* maxillary prominence, *np* nasal plug, *oe* sensory olfactory epithelium, *re* respiratory (non-sensory) epithelium of the nasal cavity, *ul* upper lip. *White asterisk* lumen of the VNO, nasal cavity or choanal groove.

**Additional file 4: Fig. S2.** The naso-palatal complex in the brown anole at stage 7 based on transverse histological sections at levels shown in Fig. 4D’. **A** Sections at the level of the forming nasal plug. **B** Sections at the level between the nasal plug and the entrance of the VNO to the early nasal cavity. **C** Sections at the level of the VNO. Abbreviations: *ecs* extraconchal space, *ev* early VNO, *fnm* frontonasal mass, *L1***–***L3* layers of the sensory epithelia, *lnc* lateral nasal concha, *lnp* lateral nasal prominence, *mxp* maxillary prominence, *np* nasal plug, *on* olfactory nerve, *pct* primitive choanal tube, *pn* primitive naris, *Stt* Stammteil. Scale bars 50 μm.

**Additional file 5: Fig. S3.** The naso-palatal complex in the brown anole at stage 14. **A** Lateral view of the nasal cavity (shown without the lateral nasal gland). **A’** Lateral view of the nasal cavity shown with partial opacity. **B** Dorsal view of the semi-transparent snout. **C** Ventrolateral view of the palate (semi-transparent). **C’** Ventrolateral view of the palate (opaque). Abbreviations: *a* anterior, *chf* choanal fold, *dng* main duct of the lateral nasal gland, *dv* duct of the VNO, *e* eye, *ecs* extraconchal space, *en* external naris, *et* egg tooth, *ict* inner choanal tube, *lcf* lateral choanal fissure, *lnc* lateral nasal concha, *mxf* maxillary fold, *np* nasal plug, *och* outer choana, *oct* outer choanal tube, *oe* olfactory epithelium, *pp* premaxillary papilla, *scf* subconchal fold, *Stt* Stammteil, *vc* vomerine cushion, *ves* vestibulum, *vf* vomeronasal fenestra, *vvc* vestibular ventral channel. *Green star* narrowed part of the outer choanal tube, *green asterisk* anterior extension of the lateral nasal concha, *red arrow* the anterior tip of the lacrimal duct, *white arrow* antorbital space. Note: italicized labels on 3D images indicate the concavities in rendered structures. Colours of 3D structures as in Fig. 9. Scale bars 250 μm.

**Additional file 6: Fig. S4.** Transverse histological sections through the snout of the brown anole at stage 17. **A** Section at the level of the choanal groove. **B** Higher magnification of the area from the box in **A**. Abbreviations: *chf* choanal fold, *chg* choanal groove, *ecc* ectochoanal cartilage, *ict* inner choanal tube, *ld* lacrimal duct, *lng* lateral nasal gland, *mx* maxilla, *mxf* maxillary fold, *ns* nasal septum, *Stt* Stammteil, *vc* vomerine cushion. *Green asterisk* anterior extension of the lateral nasal concha. Scale bars 200 μm (**A**) and 50 μm (**B**).

**Additional file 7: Fig. S5.** Medial views of the VNO at the late developmental phase of the naso-palatal complex of the brown anole. **A** Stage 12. **B** Stage 17. **C** Stage 18. Abbreviations: *a* anterior, *dv* duct of the VNO, *e* eye, *np* nasal plug, *oe* sensory olfactory epithelium, *ves* vestibulum. Colours of 3D structures as in Fig. 9. Note that all structures (except for VNO) are rendered with partial opacity.

**Additional file 8: Movie S1.** Developmental stage 8 of the brown anole. Abbreviations: *e* eye, *el* lower eyelid, *en* external naris, *ecs* extraconchal space, *fmn* frontonasal mass, *lnp* lateral nasal prominence, *mxf* maxillary fold, *mxp* maxillary prominence, *pct* primitive choanal tube, *poch* primitive outer choana, *Stt* Stammteil, *ves* vestibulum. *Red star* primordium of the vomerine cushion, *yellow star* primordium of the anterior segment of the palate, *red arrowhead* nasolacrimal groove. Colours of 3D structures as in Fig. 5.

## Data Availability

The image data used in this study are part of an ongoing research project and therefore have not yet been made pubic but are available from the corresponding author on reasonable request.

## Abbreviations

*VNO*: Vomeronasal organ

## References

[1] Schwenk K (1994). Comparative biology and the importance of cladistic classification: a case study from the sensory biology of squamate reptiles. Biol J Linn Soc.

[2] Armstrong JA, Gamble HJ, Goldby F (1953). Observations on the olfactory apparatus and the telencephalon of *Anolis*, a microsmatic lizard. J Anat.

[3] Martínez-Marcos A, Halpern M. Evolution of olfactory and vomeronasal systems. In: Binder MD, Hirokawa N, Windhorst U, editors. Encycl Neurosci. Berlin, Heidelberg: Springer Berlin Heidelberg; 2009. p. 1264–9.

[4] Bertmar G (1981). Evolution of vomeronasal organs in vertebrates. Evolution..

[5] Fuchs H (1908). Untersuchungen über Ontogenie und Phylogenie der Gaumenbildungen bei den Wirbeltieren. Zweite Mitteilung: Über das Munddach der Rhynchocephalen, Saurier, Schlangen, Krokodile und Säuger und den Zusammenhang zwischen Mund-und Nasenhöhle bei diesen Tieren. Zeitschr für Morphol Anthropol.

[6] Bellairs AD, Boyd JD (1950). The lachrymal apparatus in lizards and snakes.-II. The anterior part of the lachrymal duct and its relationship with the palate and with the nasal and vomeronasal organs. Proc Zool Soc Lond..

[7] Young BA (1993). Evaluating hypotheses for the transfer of stimulus particles to Jacobson’s organ in snakes. Brain Behav Evol.

[8] Schwenk K (1995). Of tongues and noses: chemoreception in lizards and snakes. Trends Ecol Evol.

[9] Huang G-Z, Zhang Z, Wang D, Mason RT, Halpern M (2006). Female snake sex pheromone induces membrane responses in vomeronasal sensory neurons of male snakes. Chem Senses.

[10] Filoramo NI, Schwenk K (2009). The mechanism of chemical delivery to the vomeronasal organs in squamate reptiles: a comparative morphological approach. J Exp Zool Part Ecol Genet Physiol.

[11] Duvall D (1981). Western fence lizard (*Sceloporus occidentalis*) chemical signals. II. A replication with naturally breeding adults and a test of the Cowles and Phelan hypothesis of rattlesnake olfaction. J Exp Zool.

[12] Cooper WE (1997). Independent evolution of squamate olfaction and vomerolfaction and correlated evolution of vomerolfaction and lingual structure. Amphib-Reptil..

[13] Seydel O. Über Entwickleungsvorgänge an der Nasenhöhle und am Mundhöhlendache von Echidna nebst Beiträgen zur Morphologie des periperen Geruchsorganes und des Gaumens der Wirbeltiere. Denkschr Med-Naturw Ges Jena 6. 1899;445–532.

[14] Rehorek SJ, Firth BT, Hutchinson MN (2000). The structure of the nasal chemosensory system in squamate reptiles. 2. Lubricatory capacity of the vomeronasal organ. J Biosci.

[15] Kratzing JE. The fine structure of the olfactory and vomeronasal organs of a lizard (*Tiliqua scincoides scincoides*). Cell Tissue Res. 1975;156. 10.1007/BF00221807 1122519

[16] Wang RT, Halpern M (1980). Light and electron microscopic observations on the normal structure of the vomeronasal organ of garter snakes. J Morphol.

[17] Pratt CWM (1948). The morphology of the ethmoidal region of *Sphenodon* and lizards. Proc Zool Soc Lond.

[18] Parsons TS (1959). Nasal anatomy and the phylogeny of reptiles. Evolution..

[19] Shrivastava RK (1963). The structure and the development of the chondrocranium of *Varanus*. Okajimas Folia Anat Jpn.

[20] Kaczmarek P, Hermyt M, Rupik W. Embryology of the VNO and associated structures in the grass snake *Natrix natrix* (Squamata: Natricinae): a 3D perspective. Front Zool. 2017;14. 10.1186/s12983-017-0188-y PMC5237294 28101121

[21] Parsons TS (1967). Evolution of the nasal structure in the lower tetrapods. Am Zool.

[22] Parsons TS, Gans C (1970). The nose and Jacobson’s organ. Biology of the Reptilia.

[23] Bellairs AD (1949). Observations on the snout of *Varanus*, and a comparison with that of other lizards and snakes. J Anat.

[24] Bellairs AD, Boyd JD (1947). The lachrymal apparatus in lizards and snakes.-I. the brille, the orbital glands, lachrymal canaliculi and origin of the lachrymal duct. Proc Zool Soc Lond..

[25] Rehorek SJ (1997). Squamate Harderian gland: an overview. Anat Rec.

[26] Souza NM, Maggs DJ, Park SA, Puchalski SM, Reilly CM, Paul-Murphy J (2015). Gross, histologic, and micro-computed tomographic anatomy of the lacrimal system of snakes. Vet Ophthalmol.

[27] McDowell SB. The evolution of the tongue of snakes, and its bearing on snake origins. In: Evolutionary Biology: Volume 6. Edited by Dobzhansky T, Hecht MK, Steere WC. New York: Springer US; 1972. p. 191–273.

[28] Townsend TM, Mulcahy DG, Noonan BP, Sites JW, Kuczynski CA, Wiens JJ (2011). Phylogeny of iguanian lizards inferred from 29 nuclear loci, and a comparison of concatenated and species-tree approaches for an ancient, rapid radiation. Mol Phylogenet Evol.

[29] Pyron R, Burbrink FT, Wiens JJ (2013). A phylogeny and revised classification of Squamata, including 4161 species of lizards and snakes. BMC Evol Biol.

[30] Williams EE (1969). The ecology of colonization as seen in the zoogeography of anoline lizards on small islands. Q Rev Biol.

[31] Williams EE. The origin of faunas. Evolution of lizard congeners in a complex island fauna: a trial analysis. In: Evolutionary Biology: Volume 6. Edited by Dobzhansky T, Hecht MK, Steere WC. New York: Springer US; 1972. p. 47–89.

[32] Losos JB (1998). Contingency and determinism in replicated adaptive radiations of island lizards. Science..

[33] Nicholson KE, Crother BI, Guyer C, Savage JM. It is time for a new classification of anoles (Squamata: Dactyloidae). Zootaxa. 2012.

[34] Stebbins RC (1948). Nasal structure in lizards with reference to olfaction and conditioning of the inspired air. Am J Anat.

[35] Gabe M, Saint GH (1976). Contribution à la morphologie comparée des fosses nasales et de leurs annexes chez les Lépidosoriens.

[36] Malan ME. Contributions to the comparative anatomy of the nasal capsule and the organ of Jacobson of the Lacertilia [Doctoral dissertation]. [Stellenbosch]: Stellenbosch University; 1945.

[37] Schwenk K (1993). The evolution of chemoreception in squamate reptiles: a phylogenetic approach. Brain Behav Evol.

[38] Vitt LJ, Pianka ER (2005). Deep history impacts present-day ecology and biodiversity. Proc Natl Acad Sci.

[39] Rieppel O, Gauthier J, Maisano J (2008). Comparative morphology of the dermal palate in squamate reptiles, with comments on phylogenetic implications. Zool J Linnean Soc.

[40] Haas G (1947). Jacobson’s organ in the chameleon. J Morphol.

[41] Hallermann J (1998). The ethmoidal region of *Dibamus taylori* (Squamata: Dibamidae), with a phylogenetic hypothesis on dibamid relationships within Squamata. Zool J Linnean Soc.

[42] Bernstein P (1999). Morphology of the nasal capsule of *Heloderma suspectum* with comments on the systematic position of helodermatids (Squamata: Helodermatidae). Acta Zool.

[43] Rehorek SJ, Firth BT, Hutchinson MN (2000). Can an orbital gland function in the vomeronasal sense? A study of the pygopodid Harderian gland. Can J Zool.

[44] Holtzman DA, Halpern M (1990). Embryonic and neonatal development of the vomeronasal and olfactory systems in garter snakes (*Thamnophis* spp.). J Morphol.

[45] Holtzman DA, Halpern M (1991). Incorporation of3H-thymidine in the embryonic vomeronasal and olfactory epithelia of garter snakes. J Comp Neurol.

[46] Slabý O (1981). Morphogenesis of the nasal apparatus in sauropsida. IV. Morphogenesis of the nasal capsule, epithelial nasal tube and organ of Jacobson in a member of the family Agamidae. Folia Morphol (Warsz).

[47] Slabý O (1982). Morphogenesis of the nasal capsule, the epithelial nasal tube and the organ of Jacobson in Sauropsida. VII. Morphogenesis and phylogenetic morphology of the nasal apparatus in *Calotes jubatus* O. B. Folia Morphol (Warsz).

[48] Slabý O (1982). Morphogenesis of the nasal capsule, the epithelial nasal tube and the organ of Jacobson in Sauropsida. VI. Morphogenesis of the nasal apparatus in *Iguana iguana* Shaw and morphological interpretation of the individual structures. Folia Morphol (Warsz).

[49] Sapoznikov O, Cizek P, Tichy F (2016). Development of olfactory epithelium and associated structures in the green iguana, *Iguana iguana* —light and scanning electron microscopic study. PeerJ.

[50] Koch NM, Gauthier JA (2018). Noise and biases in genomic data may underlie radically different hypotheses for the position of Iguania within Squamata. PLoS One.

[51] Skawiński T, Borczyk B (2017). Evolution of developmental sequences in lepidosaurs. PeerJ..

[52] Shine R, Amiel J, Munn AJ, Stewart M, Vyssotski AL, Lesku JA (2015). Is “cooling then freezing” a humane way to kill amphibians and reptiles?. Biol Open.

[53] Rollings N, Friesen CR, Whittington CM, Johansson R, Shine R, Olsson M. Sex And tissue‐specific differences in telomere length in a reptile. Ecol Evol. 2019;9:6211–9. . 10.1002/ece3.5164 PMC6580261 31236215

[54] Sanger TJ, Losos JB, Gibson-Brown JJ (2008). A developmental staging series for the lizard genus *Anolis*: a new system for the integration of evolution, development, and ecology. J Morphol.

[55] Swadźba E, Maślak R, Rupik W (2009). Light and scanning microscopic studies of integument differentiation in the grass snake *Natrix natrix* L. (Lepidosauria, Serpentes) during embryogenesis. Acta Zool.

[56] Hermyt M, Kaczmarek P, Kowalska M, Rupik W (2017). Development of the egg tooth – the tool facilitating hatching of squamates: lessons from the grass snake *Natrix natrix*. Zool Anz.

[57] Schneider CA, Rasband WS, Eliceiri KW (2012). NIH image to ImageJ: 25 years of image analysis. Nat Methods.

[58] Degenhardt K, Wright AC, Horng D, Padmanabhan A, Epstein JA (2010). Rapid 3D phenotyping of cardiovascular development in mouse embryos by micro-CT with iodine staining. Circ Cardiovasc Imaging.

[59] Metscher BD (2009). MicroCT for comparative morphology: simple staining methods allow high-contrast 3D imaging of diverse non-mineralized animal tissues. BMC Physiol.

[60] Metscher BD (2011). X-ray microtomographic imaging of intact vertebrate embryos. Cold Spring Harb Protoc.

[61] Metscher BD (2009). MicroCT for developmental biology: a versatile tool for high-contrast 3D imaging at histological resolutions. Dev Dyn.

[62] Rupik W (2012). Hollowing or cavitation during follicular lumen formation in the differentiating thyroid of grass snake *Natrix natrix* L. (Lepidosauria, Serpentes) embryos? An ultrastructural study. Zoology..

[63] Swadźba E, Rupik W (2012). Cross-immunoreactivity between the LH1 antibody and cytokeratin epitopes in the differentiating epidermis of embryos of the grass snake *Natrix natrix* L. during the end stages of embryogenesis. Protoplasma..

[64] Limaye A. Drishti: a volume exploration and presentation tool. Proc. SPIE 8506, Developments in X-Ray Tomography VIII; 2012. p. 85060X.

[65] Beecker A (1903). Vergleichende Stilistik der Nasenregion bei den Sauriern. Vögeln und Säugethieren Gegenbaurs Morphol Jahrb.

[66] Buchtová M, Boughner JC, Fu K, Diewert VM, Richman JM (2007). Embryonic development of *Python sebae* – II: craniofacial microscopic anatomy, cell proliferation and apoptosis. Zoology..

[67] Abramyan J, Thivichon-Prince B, Richman JM (2015). Diversity in primary palate ontogeny of amniotes revealed with 3D imaging. J Anat.

[68] Abramyan J, Richman JM (2015). Recent insights into the morphological diversity in the amniote primary and secondary palates. Dev Dyn.

[69] Parsons TS. Studies on the comparative embryology of the reptilian nose. Bull Mus Comp Zool Harv 1959 120;101–277.

[70] Nawshad A (2008). Palatal seam disintegration: to die or not to die? That is no longer the question. Dev Dyn.

[71] Albawaneh Z, Ali R, Abramyan J. Novel insights into the development of the avian nasal cavity. Anat Rec. 2020. 10.1002/ar.24349 PMC7436334 31872940

[72] Weber R (1950). Transitorische Verschlüsse von Fernsinnesorganen in der Embryonalperiode bei Amnioten. Recl Zool Suisse.

[73] Rudin W (1974). Untersuchungen am olfaktorischen System der Reptilien. Cells Tissues Organs.

[74] Dendy A (1899). Memoirs: outlines of the development of the tuatara, Sphenodon (Hatteria) punctatus. J Cell Sci.

[75] Masumoto H, Katori Y, Kawase T, Cho BH, Murakami G, Shibata S (2010). False positive reactivity of a substance P-antibody in the ectodermal/epithelial plug of the nose, ear, eye and perineum of the human and mouse fetuses. Okajimas Folia Anat Jpn.

[76] Kumoi T, Nishimura Y, Shiota K (1993). The embryologic development of the human anterior nasal aperture. Acta Otolaryngol (Stockh).

[77] Kim JH, Jin ZW, Murakami G, Cho BH (2016). Characterization of mesenchymal cells beneath cornification of the fetal epithelium and epidermis at the face: an immunohistochemical study using human fetal specimens. Anat Cell Biol.

[78] Glücksmann A (1951). Cell deaths in normal vertebrate ontogeny. Biol Rev.

[79] Danescu A, Mattson M, Dool C, Diewert VM, Richman JM (2015). Analysis of human soft palate morphogenesis supports regional regulation of palatal fusion. J Anat.

[80] Bourke JM, Porter WR, Witmer LM (2018). Convoluted nasal passages function as efficient heat exchangers in ankylosaurs (Dinosauria: Ornithischia: Thyreophora). PLoS One.

[81] Haller G (1921). Über den Gaumen der amnioten Wirbeltiere: I. Teil. Über den Gaumen der Reptilien. Z Für Anat Entwicklungsgeschichte..

[82] Vidal N, Hedges SB (2009). The molecular evolutionary tree of lizards, snakes, and amphisbaenians. C R Biol.

[83] Gauthier JA, Kearney M, Maisano JA, Rieppel O, Behlke ADB (2012). Assembling the squamate tree of life: perspectives from the phenotype and the fossil record. Bull Peabody Mus Nat Hist.

[84] Simões TR, Caldwell MW, Tałanda M, Bernardi M, Palci A, Vernygora O (2018). The origin of squamates revealed by a middle Triassic lizard from the Italian Alps. Nature..

[85] Ferguson MWJ (1981). The structure and development of the palate in *Alligator mississippiensis*. Arch Oral Biol.

[86] Ferguson MWJ. Palatal shelf elevation in the Wistar rat fetus. J Anat. 1978;125:555–7. . PMC1235623 640958

[87] Bush JO, Jiang R (2012). Palatogenesis: morphogenetic and molecular mechanisms of secondary palate development. Development..

[88] Ferguson M (1985). Reproductive biology and embryology of the crocodilians. In: Gans C, Billett F, Maderson P, editors. Biology of the Reptilia. Volume 14: development.

[89] Göppert E (1903). Die Bedeutung der Zunge für den sekundären Gaumen und den Ductus nasopharyngeus. Morphol Jahrb.

[90] Haller G (1922). Über den Gaumen der amnioten Wirbeltiere: II. Teil. Über den Gaumen der Säugetiere. Z Für Anat Entwicklungsgeschichte..

[91] Ferguson MWJ (1988). Palate development. Development..

[92] Shah RM, Cheng KM, MacKay RA, Wong A (1987). Secondary palate development in the domestic duck (khaki Campbell). An electron microscopic, histochemical, autoradiographic and biochemical study. J Anat.

[93] Abramyan J, Leung KJ-M, Richman JM (2014). Divergent palate morphology in turtles and birds correlates with differences in proliferation and *BMP2* expression during embryonic development. J Exp Zoolog B Mol Dev Evol.

[94] Witmer LM (1995). Homology of facial structures in extant archosaurs (birds and crocodilians), with special reference to paranasal pneumaticity and nasal conchae. J Morphol.

[95] Uetz P. The reptile database. 2019 [Accessed 31 May 2019]. Available from: http://www.reptile-database.org.

[96] Zheng Y, Wiens JJ (2016). Combining phylogenomic and supermatrix approaches, and a time-calibrated phylogeny for squamate reptiles (lizards and snakes) based on 52 genes and 4162 species. Mol Phylogenet Evol.

[97] Takami S (2002). Recent progress in the neurobiology of the vomeronasal organ. Microsc Res Tech.

[98] Dawley EM (2017). Comparative morphology of plethodontid olfactory and vomeronasal organs: how snouts are packed. Herpetol Monogr.

[99] Quinzio SI, Reiss JO (2018). The ontogeny of the olfactory system in ceratophryid frogs (Anura, Ceratophryidae). J Morphol.

[100] Smith TD, Bhatnagar KP, Squire L (2009). Vomeronasal system evolution. New encyclopedia of neuroscience.

[101] Yohe LR, Hoffmann S, Curtis A. Vomeronasal and olfactory structures in bats revealed by DiceCT clarify genetic evidence of function. Front Neuroanat. 2018;12. 10.3389/fnana.2018.00032 PMC5953337 29867373

[102] Broom R (1906). On the organ of Jacobson in *Sphenodon*. J Linn Soc Lond Zool.

[103] Takami S, Hirosawa K (1990). Electron microscopic observations on the vomeronasal sensory epithelium of a crotaline snake, *Trimeresurus flavoviridis*. J Morphol.

[104] Wang RT, Halpern M (1980). Scanning electron microscopic studies of the surface morphology of the vomeronasal epithelium and olfactory epithelium of garter snakes. Am J Anat.

[105] Saito S, Oikawa T, Taniguchi K, Taniguchi K (2010). Fine structure of the vomeronasal organ in the grass lizard, *Takydromus tachydromoides*. Tissue Cell.

[106] Allison AC (1953). The morphology of the olfactory system in the vertebrates. Biol Rev.

[107] Moulton DG. The olfactory pigment. In: Beidler L.M. (eds) Olfaction. Handbook of Sensory Physiology, vol 4/1. Springer, Berlin, Heidelberg; 1971. p. 59–74.

[108] Mendoza AS, Szabó K (1988). Developmental studies on the rat vomeronasal organ: vascular pattern and neuroepithelial differentiation. II Electron microscopy. Dev Brain Res.

[109] Garrosa M, Coca S (1991). Postnatal development of the vomeronasal epithelium in the rat: an ultrastructural study. J Morphol.

[110] Siegel DS, Taylor MS, Sever DM, Trauth SE (2018). The lack of nasolacrimal ducts in plethodontid salamanders?. Anat Rec.

[111] Hillenius WJ, Watrobski LK, Rehorek SJ (2001). Passage of tear duct fluids through the nasal cavity of frogs. J Herpetol.

[112] Rehorek SJ, Legenzoff EJ, Carmody K, Smith TD, Sedlmayr JC (2005). Alligator tears: a reevaluation of the lacrimal apparatus of the crocodilians. J Morphol.

[113] Rehorek SJ, Holland JR, Johnson JL, Caprez JM, Cray J, Mooney MP (2011). Development of the lacrimal spparatus in the rabbit (*Oryctolagus cuniculus*) and its potential role as an animal model for humans. Anat Res Int.

[114] Rossie JB, Smith TD (2007). Ontogeny of the nasolacrimal duct in primates: functional and phylogenetic implications. J Anat.

[115] Nowack C, Wöhrmann-Repenning A (2010). The nasolacrimal duct of anuran amphibians: suggestions on its functional role in vomeronasal perception. J Anat.

[116] Schmidt A, Wake MH (1990). Olfactory and vomeronasal systems of caecilians (Amphibia: Gymnophiona). J Morphol.

[117] Halpern M, Martínez-Marcos M (2003). Structure and function of the vomeronasal system: an update. Prog Neurobiol.

[118] Rehorek SJ, Hillenius WJ, Quan W, Halpern M (2000). Passage of Harderian gland secretions to the vomeronasal organ of *Thamnophis sirtalis* (Serpentes: Colubridae). Can J Zool.

[119] de la Cuadra-Blanco C, Peces-Peña MD, Jáñez-Escalada L, Mérida-Velasco JR (2006). Morphogenesis of the human excretory lacrimal system. J Anat.

[120] Wise PAD, Vickaryous MK, Russell AP (2009). An embryonic staging table for *in ovo* development of *Eublepharis macularius* , the leopard gecko. Anat Rec Adv Integr Anat Evol Biol.

[121] Boback SM, Dichter EK, Mistry HL (2012). A developmental staging series for the African house snake, *Boaedon* (*Lamprophis*) *fuliginosus*. Zoology..

[122] Roscito JG, Rodrigues MT (2012). Embryonic development of the fossorial gymnophthalmid lizards *Nothobachia ablephara* and *Calyptommatus sinebrachiatus*. Zoology..

[123] Paulsen F, Thale A, Kohla G, Schauer R, Rochels R, Parwaresch R (1998). Functional anatomy of human lacrimal duct epithelium. Anat Embryol (Berl).

[124] Hoppe G (1934). Das Geruchsorgan von Hatteria punctata. Z Für Anat Entwicklungsgeschichte.

[125] Lemire M (1985). Contribution à l’étude des fosses nasales des sauriens: anatomie fonctionnelle de la glande “à sels” des lézards déserticoles.

[126] Nicholson KE, Mijares-Urrutia A, Larson A (2006). Molecular phylogenetics of the *Anolis onca* series: a case history in retrograde evolution revisited. J Exp Zoolog B Mol Dev Evol..

[127] Irschick DJ, Austin CC, Petren K, Fisher RN, Losos JB, Ellers O (1996). A comparative analysis of clinging ability among pad-bearing lizards. Biol J Linn Soc.

[128] Losos JB, Queiroz KD (1997). Evolutionary consequences of ecological release in Caribbean *Anolis* lizards. Biol J Linn Soc.

[129] Beuttell K, Losos JB (1999). Ecological morphology of Caribbean anoles. Herpetol Monogr.

[130] Baeckens S, Driessens T, Van Damme R (2016). Intersexual chemo-sensation in a “visually-oriented” lizard, *Anolis sagrei*. PeerJ.

[131] Greenberg N (1993). Central and endocrine aspects of tongue-flicking and exploratory behavior in *Anolis carolinensis*. Brain Behav Evol.

[132] Gravelle K, Simon CA. Field observations on the use of the tongue-Jacobson’s organ system in two iguanid lizards, *Sceloporus jarrovi* and *Anolis trinitatis*. Copeia 1980;1980:356.

[133] Schwenk K (1985). Occurrence, distribution and functional significance of taste buds in lizards. Copeia..

[134] Schwenk K (1993). Are geckos olfactory specialists?. J Zool.

[135] Baeckens S, Van Damme R, Cooper WE (2017). How phylogeny and foraging ecology drive the level of chemosensory exploration in lizards and snakes. J Evol Biol.

