# Supplementary material for: Development of the squamate naso-palatal complex: detailed 3D analysis of the vomeronasal organ and nasal cavity in the brown anole Anolis sagrei (Squamata: Iguania)
Source: Front Zool. 2020 Sep 22;17:28. doi: 10.1186/s12983-020-00369-7 (PMC7507828; doi:10.1186/s12983-020-00369-7)
Supplement: Supplementary file 1 — Additional file 1: Table S1. Numbers of the brown anole embryos used for light microscopy (LM) and microtomography (XRM). [file 12983_2020_369_MOESM1_ESM.pdf]

Table S1. Numbers of the brown anole embryos used for light microscopy (LM) and microtomography (XRM).

Developmental phase of the naso-palatal complex	Stage	LM	mCT
**Early**	2	1	0
	3	3	1
	4	3	0
	5–5/6*	1	1
**Middle**	6	2	1
	7	3	1
	8	2	1
	9	3	1
	9/10	2	0
	10	1	0
**Late**	11–12*	2	1
	13	2	0
	14	1	1
	16	1	1
	17	1	1
	18	2	1
	19	1	1
Total		31	12

* no significant changes between developmental stages
