# Supplementary material for: Development of the squamate naso-palatal complex: detailed 3D analysis of the vomeronasal organ and nasal cavity in the brown anole Anolis sagrei (Squamata: Iguania)
Source: Front Zool. 2020 Sep 22;17:28. doi: 10.1186/s12983-020-00369-7 (PMC7507828; doi:10.1186/s12983-020-00369-7)
Supplement: Supplementary file 2 — Additional file 2: Table S2. Microtomography scanning parameters. (PDF 91 kB) [file 12983_2020_369_MOESM2_ESM.pdf]

Table S2. Microtomography scanning parameters.

No	Sample	Stage	Scanner	Voltage, current	Voxel size	Number of images	Exposure time
**1**	**As3**	**3**	Xradia MicroXCT-200*	20 kV, 200 µA	1.89 µm	1601	9.0 s
**2**	**As5**	**5**		20 kV, 160 µA	2.09 µm	1601	12 s
**3**	**As6**	**6**	Xradia MicroXCT-200**	40 kV, 125 µA	2.00 µm	931	20.0 s
**4**	**As7**	**7**	Xradia MicroXCT-200*	25 kV, 160 µA	2.20 µm	1601	15.0 s
**5**	**As8**	**8**	Xradia MicroXCT-200**	60 kV, 83 µA	2.10 µm	931	20.0 s
**6**	**As9**	**9**	Xradia MicroXCT-400***	40 kV, 250 µA	2.37 µm	985	2.37 s
**7**	**As12**	**12**	Xradia MicroXCT-200*	25 kV, 160 µA	2.09 µm	1601	15.0 s
**8**	**As14**	**14**		25 kV, 160 µA	2.16 µm	1601	18.0 s
**9**	**As16**	**16**		30 kV, 133 µA	5.24 µm	1201	4.50 s
**10**	**As17**	**17**		25 kV, 160 µA	2.08 µm	1601	13.0 s
**11**	**As18**	**18**	Xradia MicroXCT-400***	40 kV, 250 µA	2.37 µm	985	4.4 s
**12**	**As19**	**19**	Xradia MicroXCT-200**	40 kV, 125 µA	2.60 µm	961	20.0 s

* Laboratory of Microtomography, Institute of Paleobiology, Polish Academy of Sciences, Warsaw

** MicroCT Imaging Lab, Department of Evolutionary Biology, University of Vienna, Vienna

*** University Research Centre Functional Materials, Warsaw University of Technology, Warsaw
