# Supplementary figures and images for: Development of the squamate naso-palatal complex: detailed 3D analysis of the vomeronasal organ and nasal cavity in the brown anole Anolis sagrei (Squamata: Iguania)
Source: Front Zool. 2020 Sep 22;17:28. doi: 10.1186/s12983-020-00369-7 (PMC7507828; doi:10.1186/s12983-020-00369-7)

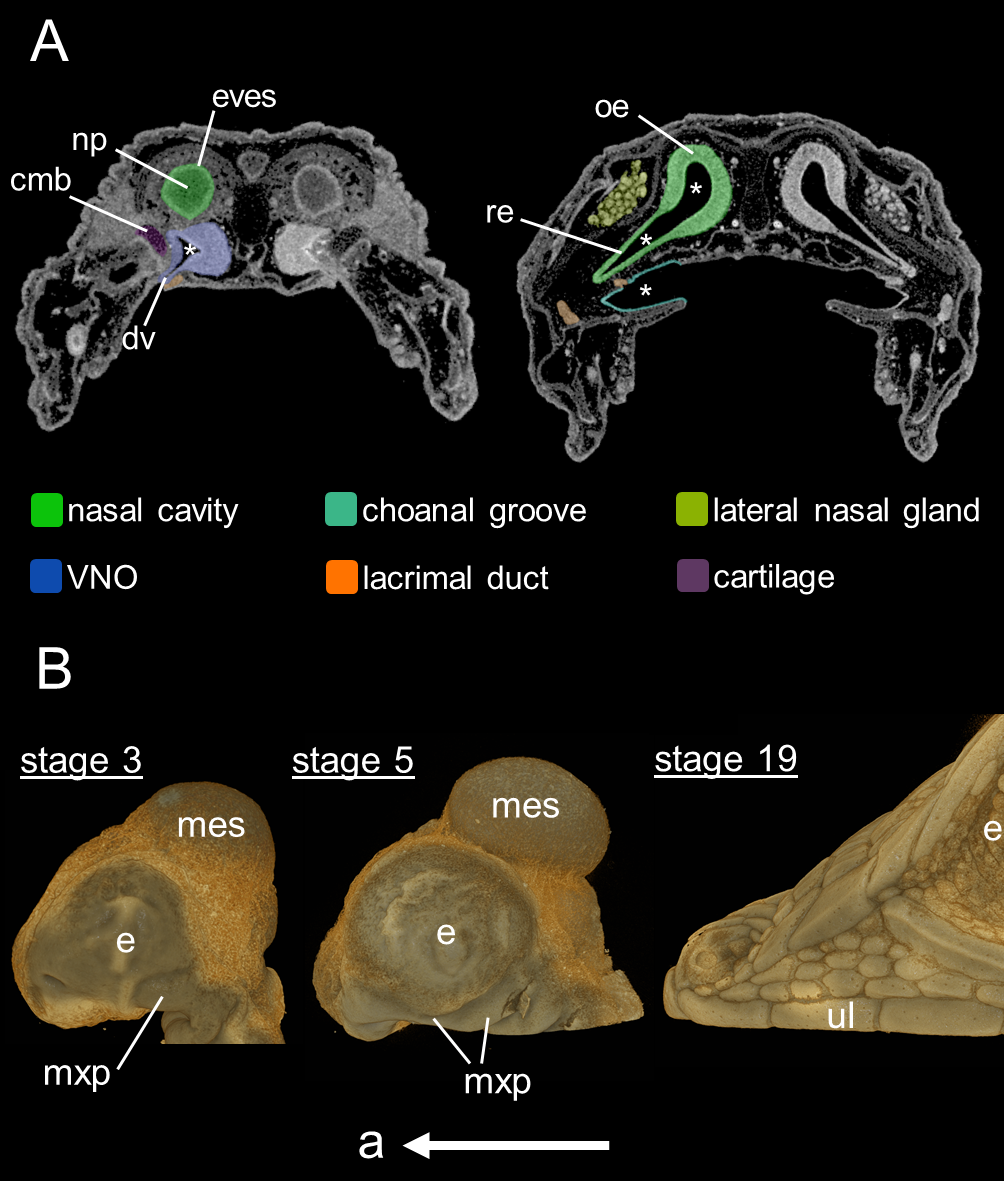

Supplement: Supplementary file 3 — Additional file 3: Fig. S1. The manner in which the segmentations were performed, illustrated with stage 17 of the brown anole (A) and alignment (for description) of the antero-posterior axis for embryos heads (B). Abbreviations: a anterior, cmb cartilage of the mushroom body, dv duct of the VNO, e eye, eves epithelium of the vestibulum, mes mesencephalon, mxp maxillary prominence, np nasal plug, oe sensory olfactory epithelium, re respiratory (non-sensory) epithelium of the nasal cavity, ul upper lip. White asterisk lumen of the VNO, nasal cavity or choanal groove. [file 12983_2020_369_MOESM3_ESM.tif]

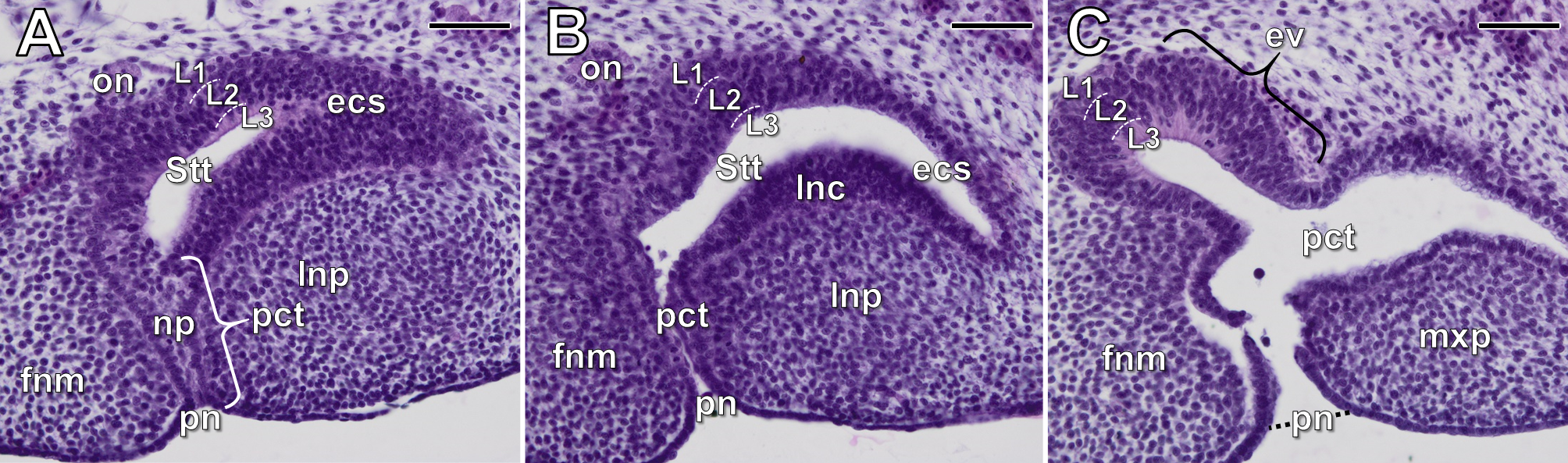

Supplement: Supplementary file 4 — Additional file 4: Fig. S2. The naso-palatal complex in the brown anole at stage 7 based on transverse histological sections at levels shown in Fig. 4D’. A Sections at the level of the forming nasal plug. B Sections at the level between the nasal plug and the entrance of the VNO to the early nasal cavity. C Sections at the level of the VNO. Abbreviations: ecs extraconchal space, ev early VNO, fnm frontonasal mass, L1–L3 layers of the sensory epithelia, lnc lateral nasal concha, lnp lateral nasal prominence, mxp maxillary prominence, np nasal plug, on olfactory nerve, pct primitive choanal tube, pn primitive naris, Stt Stammteil. Scale bars 50 μm. [file 12983_2020_369_MOESM4_ESM.tif]

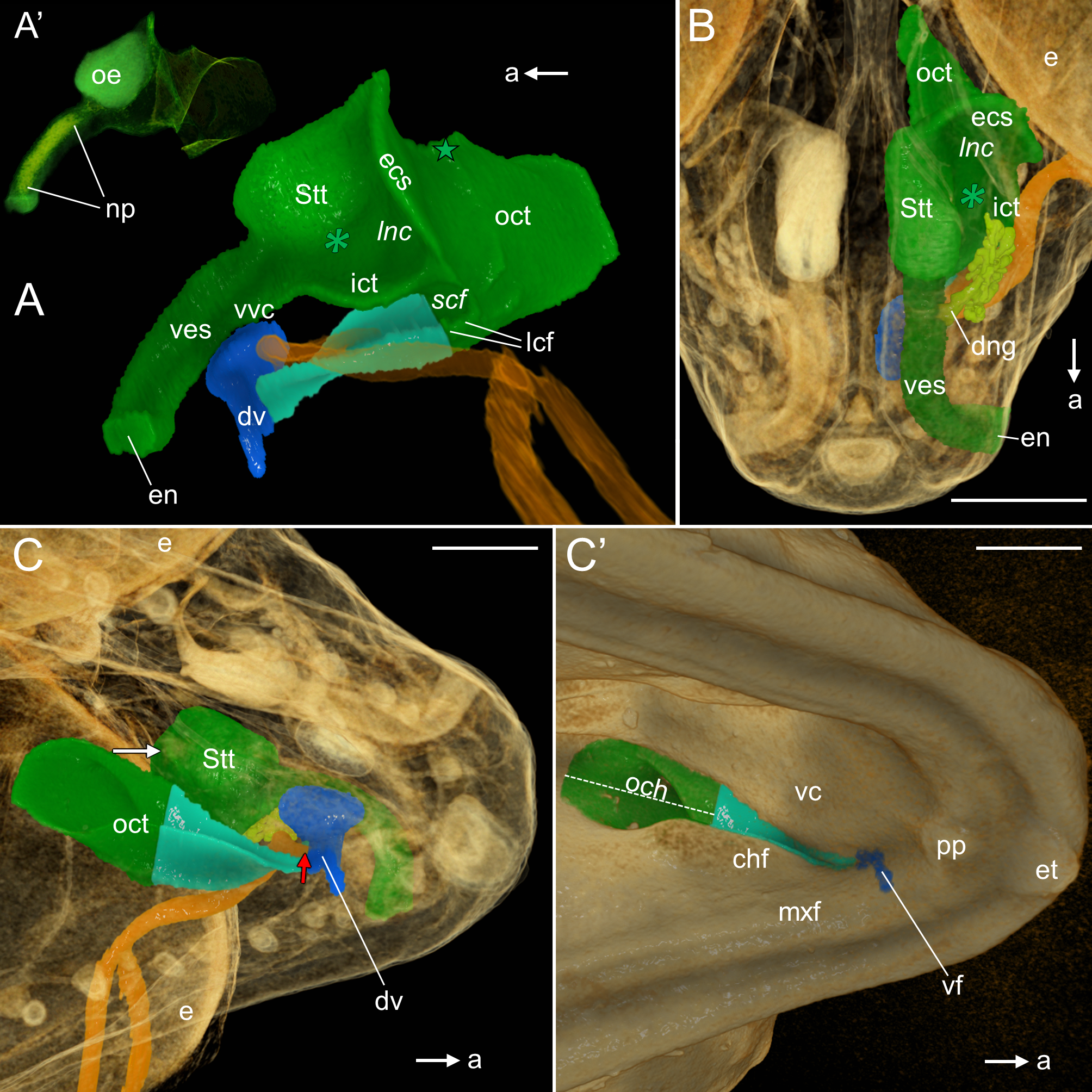

Supplement: Supplementary file 5 — Additional file 5: Fig. S3. The naso-palatal complex in the brown anole at stage 14. A Lateral view of the nasal cavity (shown without the lateral nasal gland). A’ Lateral view of the nasal cavity shown with partial opacity. B Dorsal view of the semi-transparent snout. C Ventrolateral view of the palate (semi-transparent). C’ Ventrolateral view of the palate (opaque). Abbreviations: a anterior, chf choanal fold, dng main duct of the lateral nasal gland, dv duct of the VNO, e eye, ecs extraconchal space, en external naris, et egg tooth, ict inner choanal tube, lcf lateral choanal fissure, lnc lateral nasal concha, mxf maxillary fold, np nasal plug, och outer choana, oct outer choanal tube, oe olfactory epithelium, pp premaxillary papilla, scf subconchal fold, Stt Stammteil, vc vomerine cushion, ves vestibulum, vf vomeronasal fenestra, vvc vestibular ventral channel. Green star narrowed part of the outer choanal tube, green asterisk anterior extension of the lateral nasal concha, red arrow the anterior tip of the lacrimal duct, white arrow antorbital space. Note: italicized labels on 3D images indicate the concavities in rendered structures. Colours of 3D structures as in Fig. 9. Scale bars 250 μm. [file 12983_2020_369_MOESM5_ESM.tif]

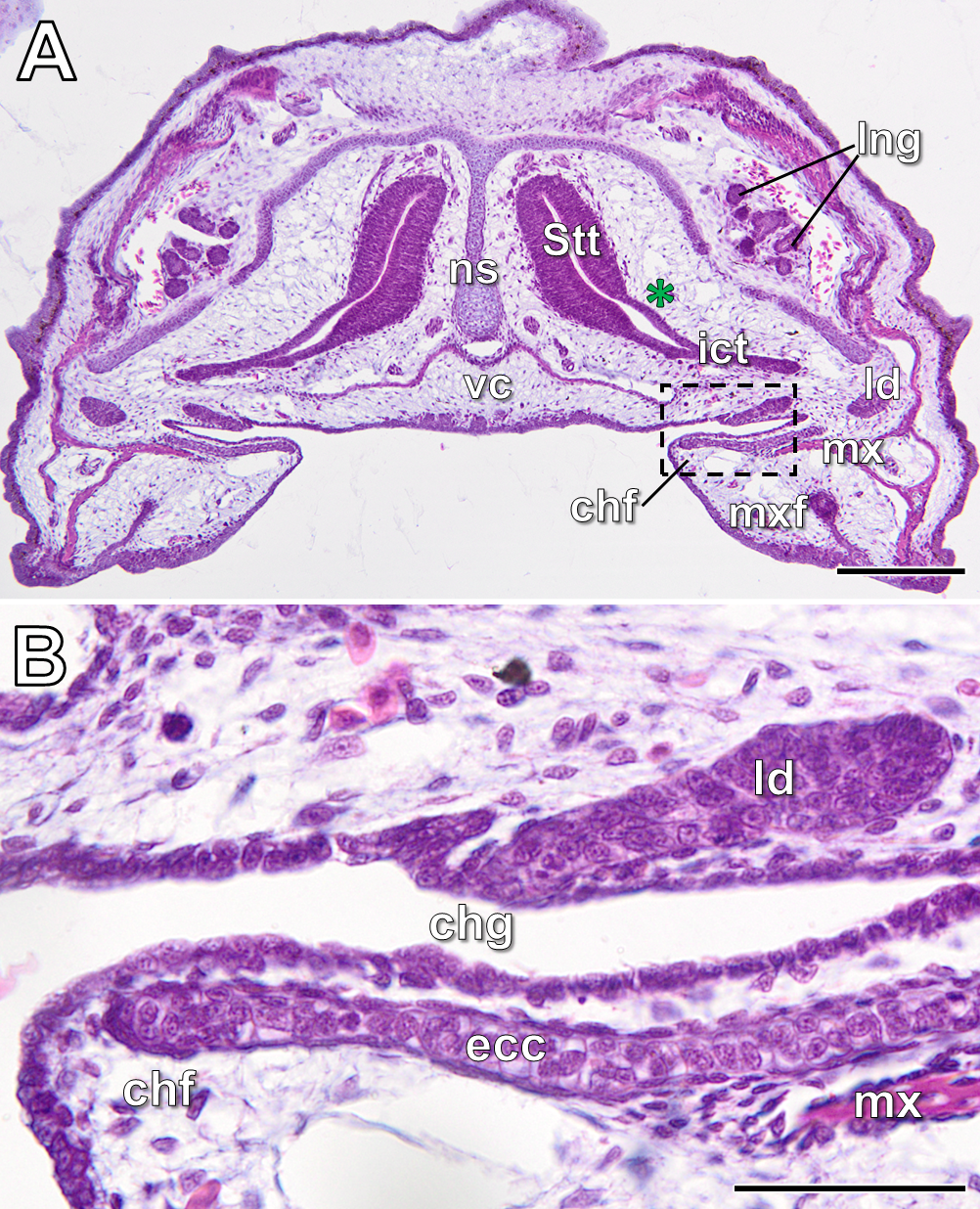

Supplement: Supplementary file 6 — Additional file 6: Fig. S4. Transverse histological sections through the snout of the brown anole at stage 17. A Section at the level of the choanal groove. B Higher magnification of the area from the box in A. Abbreviations: chf choanal fold, chg choanal groove, ecc ectochoanal cartilage, ict inner choanal tube, ld lacrimal duct, lng lateral nasal gland, mx maxilla, mxf maxillary fold, ns nasal septum, Stt Stammteil, vc vomerine cushion. Green asterisk anterior extension of the lateral nasal concha. Scale bars 200 μm (A) and 50 μm (B). [file 12983_2020_369_MOESM6_ESM.tif]

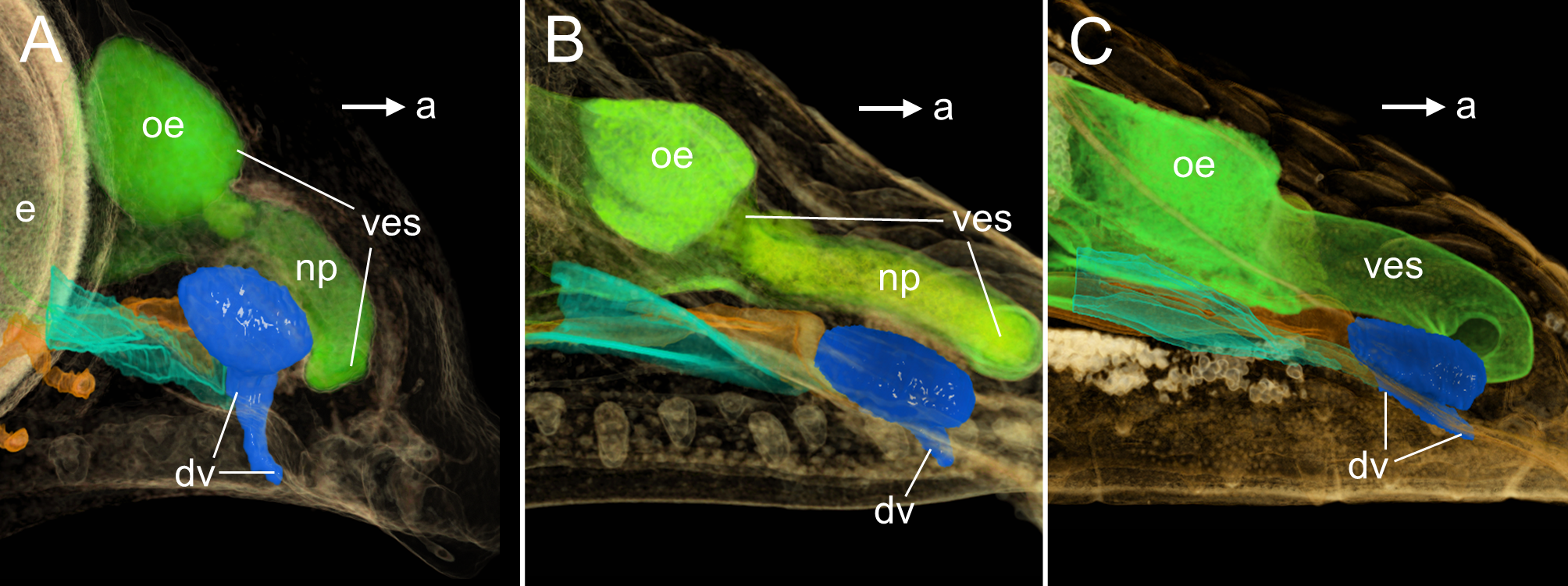

Supplement: Supplementary file 7 — Additional file 7: Fig. S5. Medial views of the VNO at the late developmental phase of the naso-palatal complex of the brown anole. A Stage 12. B Stage 17. C Stage 18. Abbreviations: a anterior, dv duct of the VNO, e eye, np nasal plug, oe sensory olfactory epithelium, ves vestibulum. Colours of 3D structures as in Fig. 9. Note that all structures (except for VNO) are rendered with partial opacity. [file 12983_2020_369_MOESM7_ESM.tif]
